# Novel *Bis*-Thiazole Derivatives: Synthesis and Potential Cytotoxic Activity Through Apoptosis With Molecular Docking Approaches

**DOI:** 10.3389/fchem.2021.694870

**Published:** 2021-08-12

**Authors:** Kamal M. Dawood, Mohamed A. Raslan, Ashraf A. Abbas, Belal E. Mohamed, Magda H. Abdellattif, Mohamed S. Nafie, Mohamed K. Hassan

**Affiliations:** ^1^ Department of Chemistry, Faculty of Science, Cairo University, Giza, Egypt; ^2^ Department of Chemistry, Faculty of Science, Aswan University, Aswan, Egypt; ^3^ Department of Chemistry, College of Science, Taif, Saudi Arabia; ^4^ Department of Chemistry, Faculty of Science, Suez Canal University, Ismailia, Egypt; ^5^ Biotechnology Program, Department of Zoology, Faculty of Science, Port Said University, Port Said, Egypt; ^6^ Center for Genomics, Helmy Institute, Zewail City for Science and Technology, Giza, Egypt

**Keywords:** docking, apoptosis, cytotoxic, bis-thiazoles, hydrazonoyl chlorides, Pim-1 kinase

## Abstract

A series of *bis*-thiazoles 5a–g were synthesized from *bis*-thiosemicarbazone 3 with hydrazonoyl chlorides 4a–g. Reaction of 3 with two equivalents of α-halocarbonyl compounds 6–8, 10, and 12a–d afforded the corresponding *bis*-thiazolidines 9, 11, and 13a–d, respectively. Condensation of *bis*-thiazolidin-4-one 9 with different aromatic aldehydes furnished *bis*-thiazolidin-4-ones 14a–d. Compounds 5a–g, 9, and 13a,c,d were screened *in vitro* for their cytotoxic activities in a panel of cancer cell lines. Compounds 5a–c, 5f–g, and 9 exhibited remarkable cytotoxic activities, especially compound 5c with potent IC_50_ value 0.6 nM (against cervical cancer, Hela cell line) and compound 5f with high IC_50_ value 6 nM (against ovarian cancer, KF-28 cell line). Compound 5f–induced appreciated apoptotic cell death was measured as 82.76% associated with cell cycle arrest at the G1 phase. The apoptotic pathways activated in KF-28 cells treated with 5a, 5b, and 5f were further investigated. The upregulation of some pro-apoptotic genes, bax and puma, and the downregulation of some anti-apoptotic genes including the Bcl-2 gene were observed, indicating activation of the mitochondrial-dependent apoptosis. Together with the molecular docking studies of compounds 5a and 5b, our data revealed potential Pim-1 kinase inhibition through their high binding affinities indicated by inhibition of phosphorylated C-myc as a downstream target for Pim-1 kinase. Our study introduces a set of *bis*-thiazoles with potent anti-cancer activities, *in vitro.*

## Introduction

**Chart 1:**
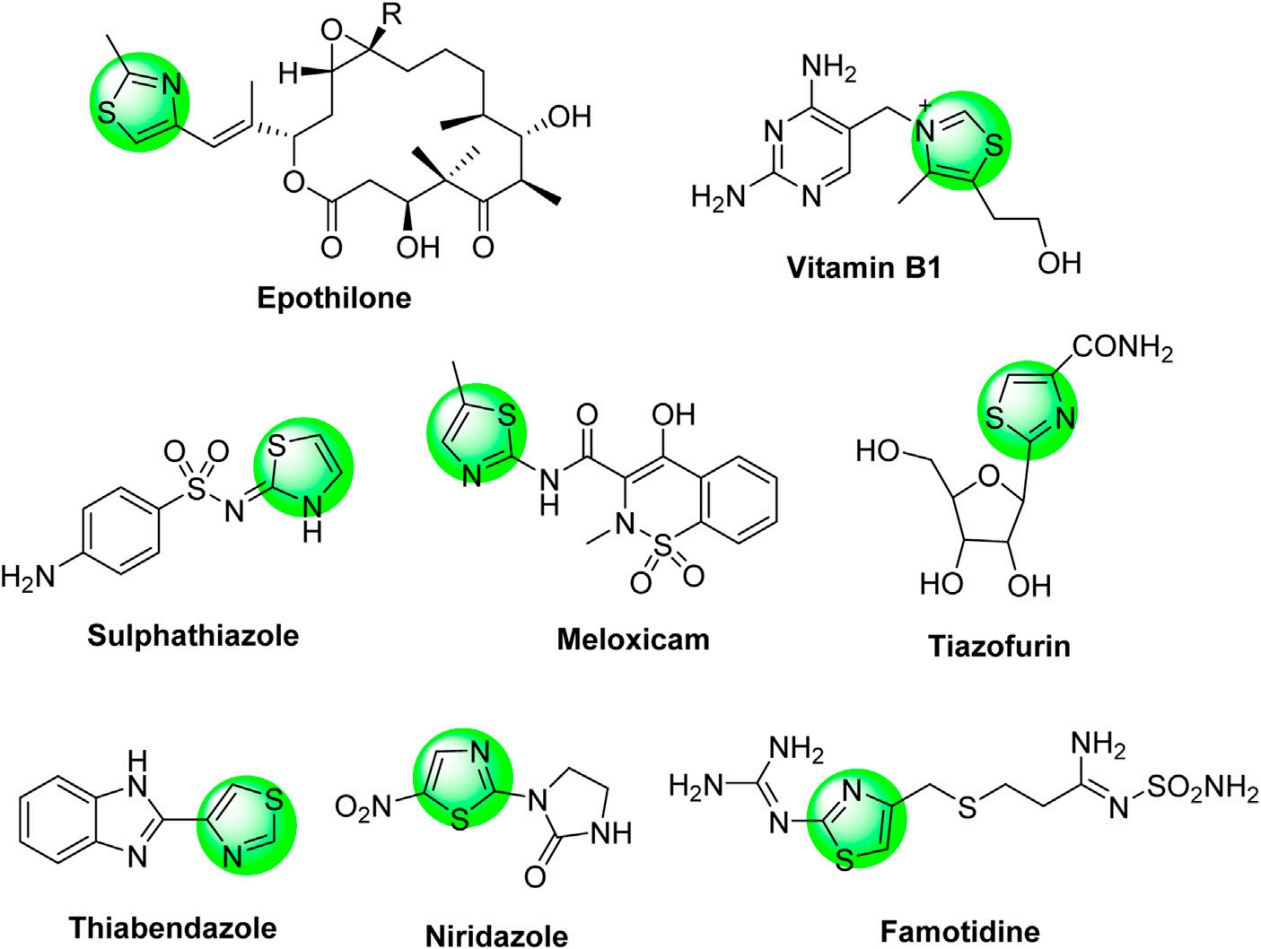
Some thiazole-based synthetic commercial drugs in the market.

Thiazole-based heterocycles are highly versatile scaffolds, characterized by pronounced pharmacological importance and widely used by medicinal chemistry researchers for drug design purposes. For example, thiazoles demonstrated antimicrobial, antiviral, anti-diabetic, diuretic, anticonvulsant, antioxidant, anti-HIV, analgesic, anti-inflammatory, neuroprotective, and antitumor activities. These wide and potent activities were extensively published in several review articles (Siddiqui et al., 2011; Leoni et al., 2014; Ayati et al., 2015; Morigi et al., 2015; Rouf and Tanyeli, 2015; Chhabria et al., 2016; Das et al., 2016; Kumar et al., 2016; Agarwal et al., 2018; Scarim et al., 2019; de Oliveira et al., 2020). The anti-cancer activities of thiazole derivatives were also intensively reviewed (Ayati et al., 2019; Sharma et al., 2020). In addition, the thiazole moiety is incorporated in several natural products such as thiamine (vitamin B1), epothilone (anti-cancer drug), bacitracin, and penicillin (antibiotic drugs). Furthermore, the thiazole moiety is an essential component of a wide range of synthetic commercial drugs in the market, such as *sulfathiazole* (sulfa-drug), *niridazole* (schistosomicide drug), *thiabendazole* (antifungal drug), *tiazofurin* (anti-cancer drug), *famotidine* (stomach’s anti-ulcer drug), and *meloxicam* (non-steroidal anti-inflammatory drug), as depicted in Chart 1.

**Chart 2:**
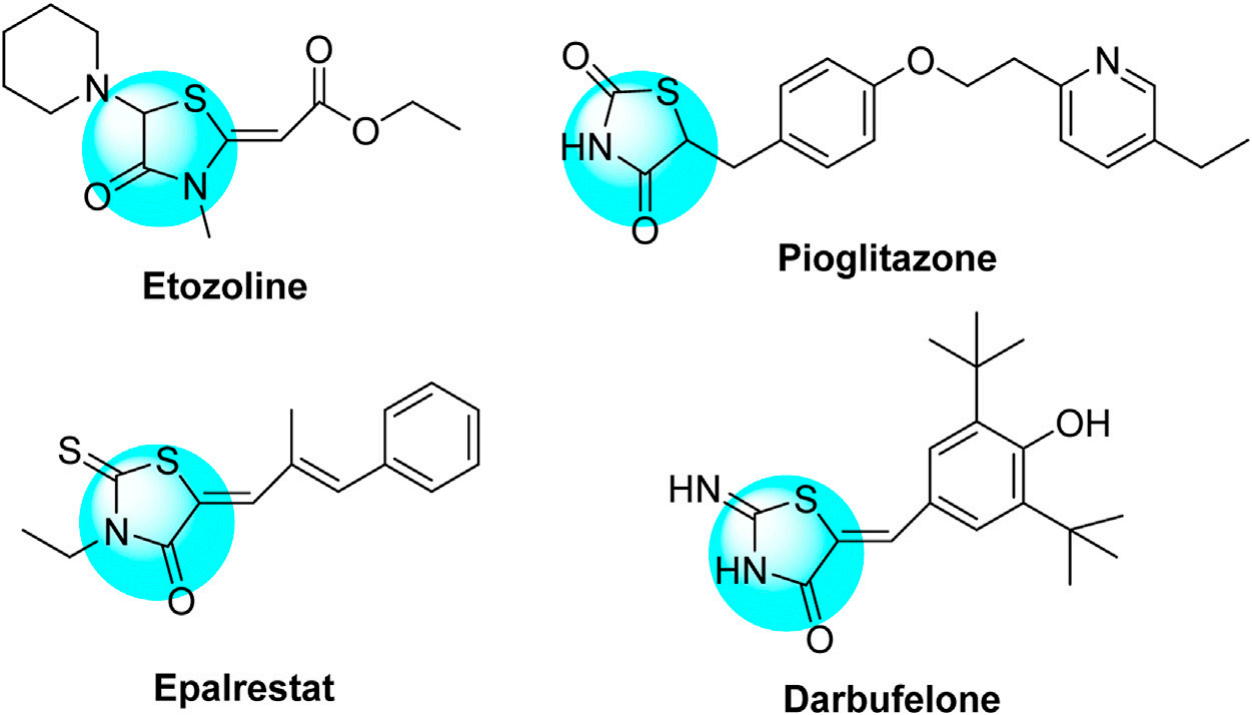
Some 4-thiazolidinone–based synthetic commercial drugs.

On the contrary, 4-thiazolidinone derivatives were also highly active heterocyclic substrates and have featured potent therapeutic inhibitory activities as published in several reviews and research articles (Jain et al., 2012; Tripathi et al., 2014; Kaminsky et al., 2017; Manjal et al., 2017; de Siqueira et al., 2019; Nirwan et al., 2019). Their biological potencies are exemplified as antibacterial, antifungal, antitubercular, anti-cancer, anti-inflammatory, analgesic, anticonvulsant, antibiofilm, antidepressant, antiviral, anti-diabetic, and antiarrhythmic activities. 4-Thiazolidinones were also reported as ingredients of several synthetic drugs such as ***pioglitazone*** and ***epalrestat*** (anti-diabetic drugs), ***etozoline*** (diuretic drug), and ***darbufelone*** (anti-inflammatory drug) (Chart 2).

Multi-component reaction (MCR) is attracting the attention of chemists for the construction of diverse heterocyclic scaffolds. MCR is a powerful synthetic methodology in which three or more reactive substrates are mixed in one reaction flask to form a new product employing all substrates with minimized by-products and good yields (Paprocki et al., 2018; de Marigorta et al., 2019; Zhi et al., 2019; Kappe, 2020).

Our research interest is focused on the synthesis and biological evaluation of thiazole-based heterocycles (Abdel-Gawad et al., 2010; Dawood and EI-Deftar, 2010; Hegazi et al., 2010; Dawood et al., 2011; Dawood et al., 2013; Dawood and Abu-Deif, 2014; Dawood et al., 2015; Dawood et al., 2015; Dawood, 2019; Mahmoud et al., 2020). Therefore, keeping the above facts in mind and inspired by the reported extraordinary bioactivity behaviors of thiazole and 4-thiazolidinone derivatives, the objective of the current study is to design and synthesize new *bis*-thiazole structures for examining their potency against several of human cancer cell lines as well as investigating their effects as an inducer of apoptosis on the tested cancer cells.

## Results and Discussion

### Chemistry

**Scheme 1:**
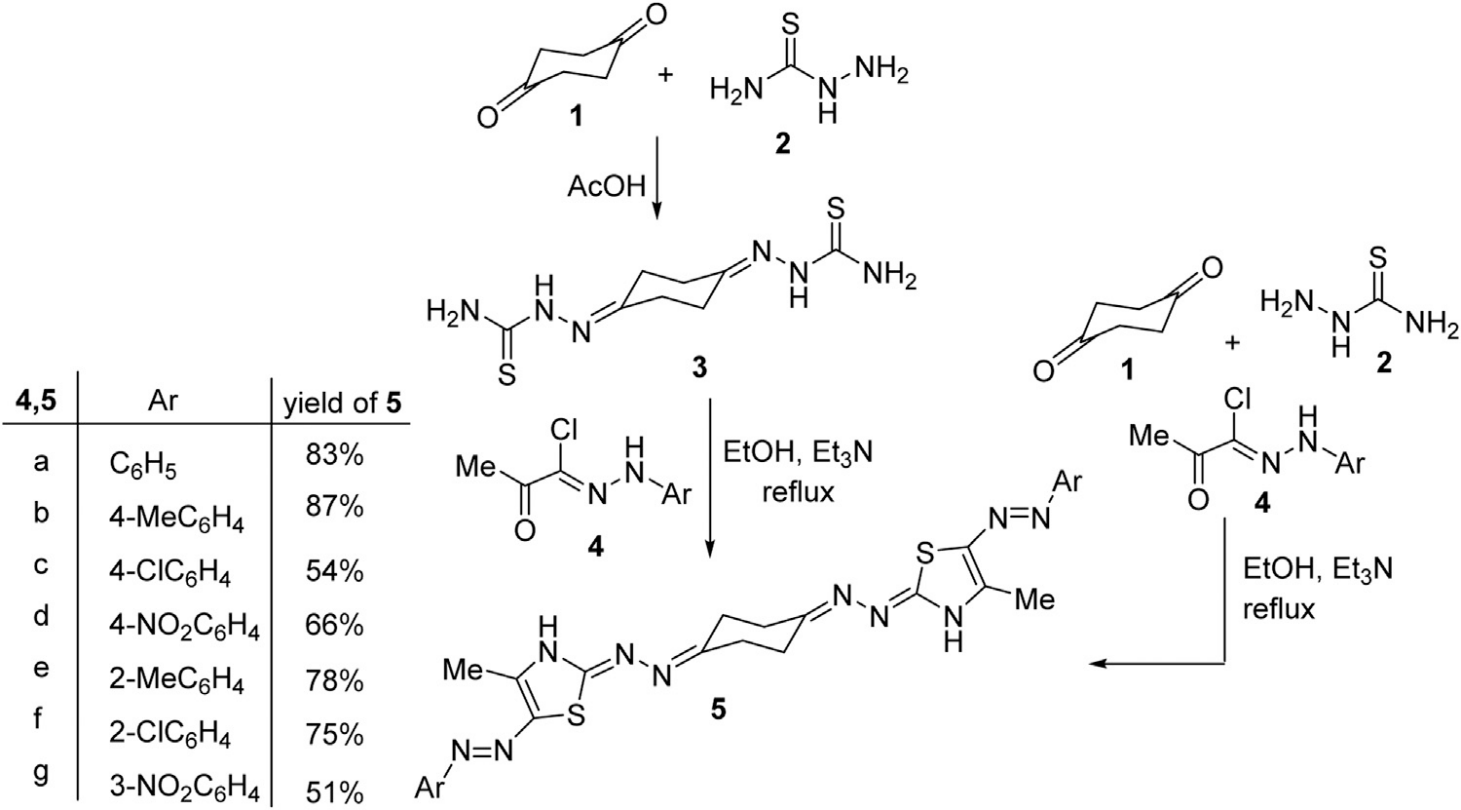
Synthesis of 1,4-bis((5-(arylazo)thiazol-2-ylidene)hydrazono)cyclohexane 5.

1,4-Cyclohexanedione-*bis*-thiosemicarbazone 3 was reported once in the literature in 1982 as an analytical reagent for spectrophotometric estimation of copper, but no more applications of this substrate in organic synthesis were published so far (Ceba et al., 1982). Thus, when 1,4-cyclohexanedione-*bis*-thiosemicarbazone 3 was treated with a number of hydrazonoyl chlorides 4a–g in ethanol at refluxing temperature using triethylamine as a base, a single product was isolated, in each case, as detected by TLC analysis. Spectral data (^1^H and ^13^C NMR, MS and IR) as well as elemental analyses confirmed the assigned *bis*-thiazole structures 5a–g, Scheme 1. The IR spectra of structures 5a–g showed an NH-stretching absorption at around 3,230–3,300 cm^−1^, and their mass spectra revealed, in each case, a peak due to their molecular ions (M^+^). The ^1^H NMR spectrum of compound 5b, for example, revealed three singlet signals at *δ* 2.25, 2.51, and 10.41 (D_2_O-exchangeable) due to the thiazole-CH_3_, *p*-tolyl, and thiazole-NH protons, respectively, and two multiplets in the regions 2.73–2.74 and 2.89–2.90 assigned to cyclohexane protons plus two doublet peaks at 7.12 and 7.23 (*J* = 8.4 Hz) corresponding to the *para*-substituted aromatic protons. The ^13^C NMR spectrum of 5b exhibited 14 peaks corresponding to six aliphatic carbon atoms and eight aromatic carbon atoms.

Interestingly, a multi-component one-pot procedure was investigated for the construction of the products 5a–c as outlined in Scheme 1. Thus, when 1,4-cyclohexanedione 1 was treated with two equivalents of both thiosemicarbazide 2 and hydrazonoyl chlorides 4a–c (as representative examples) in a one-pot flask and then dissolved in ethanol and heated at reflux temperature using two equivalents of triethylamine, to give a single product in each case, that was in complete accordance with compounds 5a–c in almost similar yields as the stepwise procedure discussed above.

**Scheme 2:**
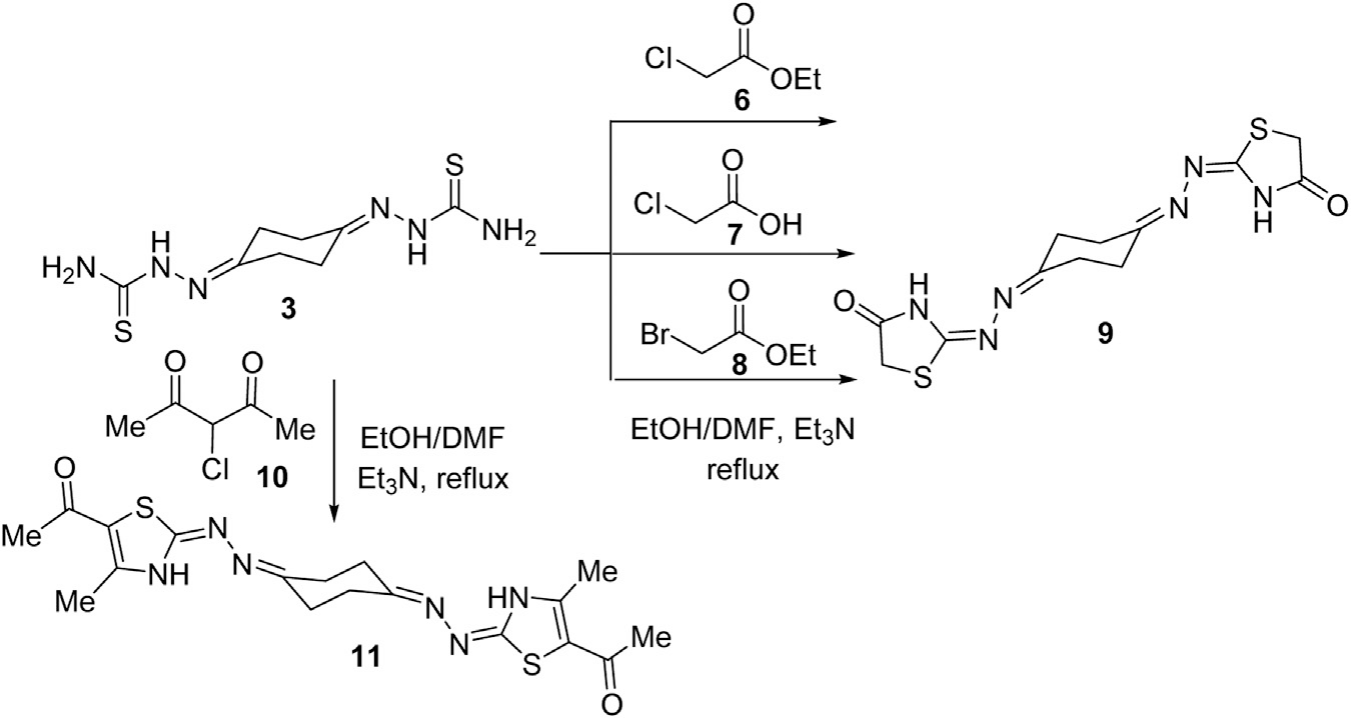
Synthesis of cyclohexane-1,4-diylidene-*bis*-thiazolidine derivatives 9 and 11.

In the same way, treatment of *bis*-thiosemicarbazone 3 with ethyl chloroacetate (6) in 1:2 M ratios in refluxing ethanol using triethylamine as a base led to production of a compound that was established as the *bis*-4-thiazolidinone derivative (9) in 73% yield (Scheme 2) based on its spectral analyses. The IR spectrum of 9 showed two peaks at 3320 and 1712 cm^−1^ assignable for NH and C=O functions, respectively. Moreover, a peak at *m/z* 338 due to the molecular ion (M^+^) appeared in its mass spectrum. The ^1^H NMR spectrum of compound **9** revealed two multiplet peaks in the regions *δ* 2.53–2.57 and 2.68–2.73 assignable to cyclohexane protons and a singlet peak at *δ* 3.81 for thiazolidinone-CH_2_ protons besides a broad peak at *δ* 11.72 (D_2_O-exchangeable) due to thiazolidinone-NH protons. ^13^C NMR of compound 9 exhibited five peaks at *δ* 25.35, 32.23, 32.72, 166, and 173.93. Two alternative methods for synthesis of the *bis*-thiazolidinone derivative 9 were conducted as shown in Scheme 2. Thus, treatment of *bis*-thiosemicarbazone 3 with chloroacetic acid (7) in ethanol solvent at reflux temperature employing sodium acetate as a base or with ethyl bromoacetate (8) in dimethylformamide and triethylamine as a base yielded the same product in 62 and 68% yields, respectively, which was identical in all aspects to compound 9 obtained above. Similarly, treatment of compound **3** with two equivalents of α-chloroacetylacetone (10), under a typical reaction procedure above, furnished the 1,4-diylidene-*bis*-thiazolidine derivative 11 in 71% yield.

**Scheme 3:**
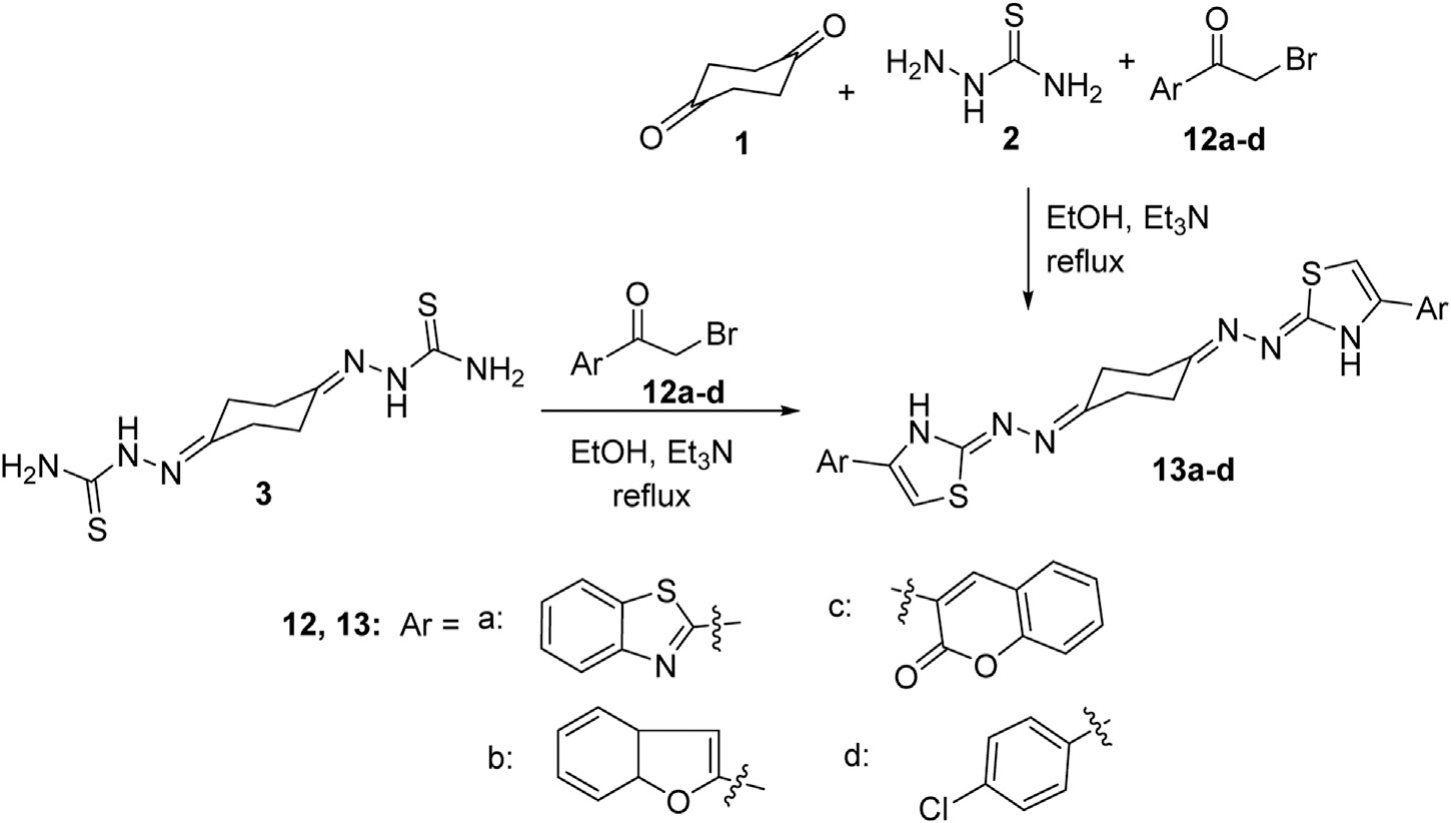
Synthesis of 1,4-*bis*((4-arylthiazol-2-ylidene)hydrazono)cyclohexanes 13a–d.

The scope of our strategy was extended to encompass synthesis of the new 1,4-*bis*((4-arylthiazol-2-ylidene)hydrazono)cyclohexane derivative 13. Thus, when *bis*-thiosemicarbazone **3** was allowed to react with 2-(bromoacetyl)benzothiazole (12a) in a 1:2 M ratio in ethanol at reflux conditions using triethylamine as a base, it furnished a single product that was established as 1,4-*bis*((4-(2-benzothiazolyl)thiazol-2-ylidene)hydrazono)-cyclohexane (13a) in 72% yield (Scheme 3) on the basis of its elemental analyses and spectroscopic data. The same product was obtained alternatively, in 77% yield, *via* a one-pot three-component procedure by adding the three substrates: 1,4-cyclohexanedione (1), thiosemicarbazide (2), and 2-(bromoacetyl)benzothiazole (12a) in 1:2:2 M ratios in one flask, dissolved in ethanol and then heated at reflux temperature and catalyzed by triethylamine. The ^1^H-NMR spectrum of compound 13a exhibited three multiplet peaks in the regions *δ* 2.58–2.71, 7.43–7.53, and 8.01–8.09 corresponding to the cyclohexane and benzothiazole protons, respectively, in addition to two singlets at *δ* 7.73 and 11.03 assignable to thiazole-5-CH and thiazole-3-NH protons, respectively. ^13^C NMR revealed four aliphatic carbon atoms at *δ* 23.94, 26.18, 29.29, and 31.50 and nine aromatic carbon atoms at 109.32, 122.34, 122.54, 125.15, 126.52, 134.54, 144.65, 153.59, and 154.11 in addition to 162.79 (C=N) and 170.37 (C=O). Similarly, reaction of *bis*-thiosemicarbazone 3 with three extra examples of α-bromoketones, namely, 2-(bromoacetyl)benzofuran (12b), 3-(bromoacetyl)coumarin-2-one (12c), and 4-chlorophenacyl bromide (12d), under a typical reaction procedure described above, gave the corresponding 1,4-*bis*((4-arylthiazol-2-ylidene)hydrazono)cyclohexane derivatives 13b–d in high yields as shown in Scheme 3.

**Scheme 4:**
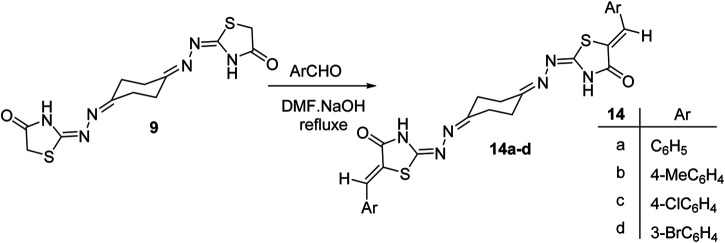
Synthesis of 1,4-bis((5-arylidene-thiazolidin-2-ylidene)hydrazono)cyclohexanes 14a–d.

Finally, synthesis of the new *bis*(thiazolidin-4-one) derivatives 14a–d was also carried out. Thus, condensation reaction of the *bis*-thiazolidin-4-one derivative 9 with a number of aromatic aldehydes in ethanol heated at reflux conditions, using piperidine as a base, afforded the 1,4-*bis*((5-arylidene)-4-oxo-thiazolidin-2-ylidene)hydrazono-cyclohexane derivatives 14a–d (Scheme 4). Structures of the obtained products were elucidated from their elemental and spectral data. The ^1^H NMR spectra of 14a–d were free of the thiazolidinone-5-CH_2_ protons of 9 at *δ* 3.81 and exhibited a singlet peak around *δ* 7.7 due to the methine = *CH* proton. The stereochemical *Z*-configuration of the exocyclic C=CH bonds in 14a–d was assigned based on their ^1^H NMR spectral data where the methine C=CH proton near *δ* 7.7 is closer to the analogously reported (*Z*)-5-arylidene-thiazolidin-4-one derivatives (Momose et al., 1991; Paprocki et al., 2018) instead of the *E*-configuration of the methine C=CH proton that resonates at *δ* < 7.5 (Karigar et al., 2011).

### Biological Activity

#### Cytotoxic Activity Against Panel of Cancer Cell Lines

**FIGURE 1:**
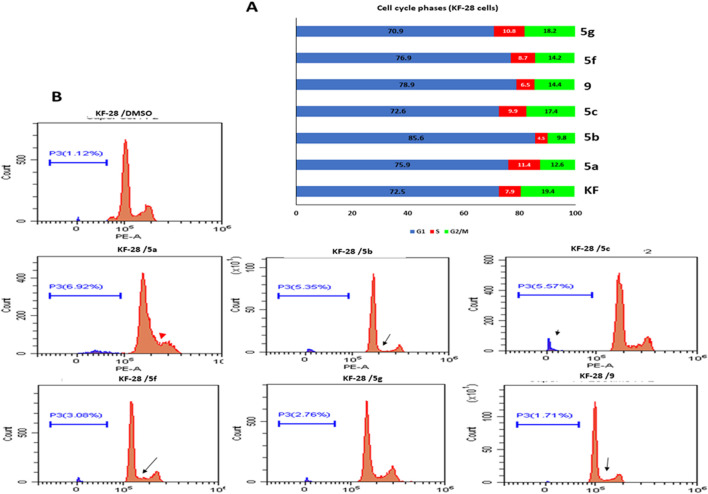
Effect of selected compounds on cell cycle phases in KF-28 cell lines. **(A)** Representative cytographs show the effect of vehicle and compounds 5a–c, 5f, 5g, and 9 on the different cell cycle phases of KF-28 cells when treated with each compound for two days. **(B)** Collected results show the effect of each compound on different populations at each cell cycle phase.

The synthesized derivatives were screened for their cytotoxic activities against five cancer cell lines (two breast cancer cell lines: MCF-7 and MDA-MB-231, two ovarian cancer cell lines: A2780 and KF-28, and one cervical cancer cell line: Hela). This study was performed using serial concentrations of 25, 50, 100, 150, and 200 µM of each compound using the MTT assay. Percentages of cell viability of the tested compounds with serial concentration against the tested panel of cell lines are depicted in Figure 1, in which there is a decrease in the cell viability with increasing concentrations in a dose–response pattern. Additionally, as seen in Table 1 and Figure 1, the MTT assay results for ovarian cancer cell lines for compound 5f exhibited a pronounced cytotoxic effect with an IC_50_ value of 0.0061 µM. In contrast, compounds 5a and 5b were notably cytotoxic with IC_50_ values of 0.718 and 3.374 µM, respectively, against the KF-28 cell line when treated for 72 h. Additionally, compounds 5f and 5g showed a remarkable cytotoxic activity with IC_50_ values 2.34 and 7.45 µM, respectively, against the A2780 cell line, when treated for 72 h. Regarding breast cancer cells, compound 5e showed a potent cytotoxic effect against MCF-7 breast cancer cells with an IC_50_ value of 0.6648 µM (72 h), while compound 5a displayed a notable cytotoxic effect with an IC_50_ value of 1.51 µM against the MDA-MB-231 cells (treated for 72 h). Finally, the results from cervical cancer, Hela, cells showed a pronounced response when treated with compound **5c** with the most significant inhibition potency having IC_50_ value 0.00065 µM (72 h treatment). Therefore, compounds 5a, 5b, and 5f were thought worthwhile to further investigate their effects as either cytotoxic or antiproliferative effects and whether any of them is an inducer of apoptotic cell death in the representative cancer cell lines KF-28 and A2780.

**TABLE 1:** Summarized IC_50_ values (µM) of the tested derivatives against MCF-7, Hela, A2780, KF-28, and MDA-MB-231 cancer cell lines.

Comp.	IC_50_ ^a^ ^,#^ (µM)					
	Breast		Cervical	Ovarian		Normal mouse embryonic fibroblast
	MCF-7	MDA-MB-231	Hela	A2780	KF-28	MEF
5a	NA	ND	NA	77.87	0.718	7.08
5b	1,014.49	NA	202.47	94.35	3.374	2.34
5c	ND	115.51	0.00065	ND	ND	NA
5d	894.86	NA	NA	NA	NA	ND
5e	0.66248	NA	248.70	70.61	NA	ND
5f	NA	753.72	1,587.91	2.34	0.0061457	NA
5g	580.96	610.18	363.0	7.45	257.34	0.167
9	306.78	779.99	111.05	103.37	86.52	1.059
13a	1,384.83	NA	1801.80	27.23	212.48	ND
13c	177.68	ND	NA	33.52	85.72	ND
13d	284.23	ND	353.1	ND	ND	ND
Cisplatin	1.207	ND	ND	1.559	9.289	ND

a Values are expressed as the average of three independent trials (*n* = 3). ND is not determined, while NA is not active with higher IC_50_ values. ^**#**^IC_50_ was calculated using the linear curve fit of log[conc.] vs. % of cell viability in Excel.

Our results agree with those of several previous studies (Turan-Zitouni et al., 2016; Kumar and Umadevi, 2018; Borcea et al., 2021), which investigated the cytotoxic activities of analogous *bis*-thiazole derivatives against various cancer cell lines with promising IC_50_ values. However, the MTT technique used just indicates the viability under different treatments; therefore, this encouraged us to further investigate the cellular and molecular mechanisms behind such cytotoxicity.

#### Apoptosis Investigation

##### DNA Content and Cell Cycle Analysis

Studying the cell cycle phases by measuring DNA content is a crucial test that helps in evaluating the impact of a given drug(s) on dividing cells and may reflect the mechanism through which this drug(s) affects those cells. Hence, we used the ovarian cancer cells (KF-28) as a representative cellular model to study the effect of the synthesized compounds on the cell cycle. Compounds 5a, 5b, 5c, 5f, 5g, and 9 were added to similar numbers of cells at 200 µM for 48 h, and then, the different cell cycle phases were assessed by flow cytometric analysis.

DNA content analysis of KF-28 cells treated with compounds 5a, 5b, 5c, 5f, 5g, and 9 showed that compound 5a significantly accumulated the cells at the S phase (red arrowhead; 11.4%), while other compounds such as 5b and 9 showed appreciated reduction in the S phase population of the cells tested measured as 4.5 and 6.5%, respectively (indicated by black arrows; Figure 1). Moreover, treating cells with 5a, 5b, 5f, and 9 compounds was associated with relative accumulation at the G1 phase indicated by the elevated percentage of G1 populations measured as 75.9, 85.6, 76.9, and 78%, respectively, compared with 72% at the G1 phase in DMSO-treated control cells. Subsequently, this effect was associated with observable relative reduction in the percentage of G2/M phase populations. Similar results were observed in the A2780 cell line when treated with the same compounds 5a, 5b, 5c, 5f, 5g, and 9 for 48 h even at lower concentrations (100 µM) (Supplementary Figure 2).

Cellular toxicity by specific molecules causing cellular arrest usually ends with cellular death in cancer cells. For instance, taxane family drugs target the microtubules causing arrest at the G2/M phase which ends up with apoptotic cell death (Hassan et al., 2011), while cisplatin causes DNA crosslinking and then arrests dividing cancer cells at G1 to end with apoptotic cell death (Galluzzi et al., 2012). Here, our data revealed that the cytotoxicity indicated by MTT might be due to arresting the cells at a certain cell cycle phase like G1 under 5a, 5b, and 5f compounds. Therefore, to further investigate whether this arrest causes cell death or just reduces cellular proliferation, we moved to studying the possible apoptotic signaling by *bis*-thiazole derivatives used in this study.

##### Annexin V-FITC/PI Differential Apoptosis Assessment

**FIGURE 2:**
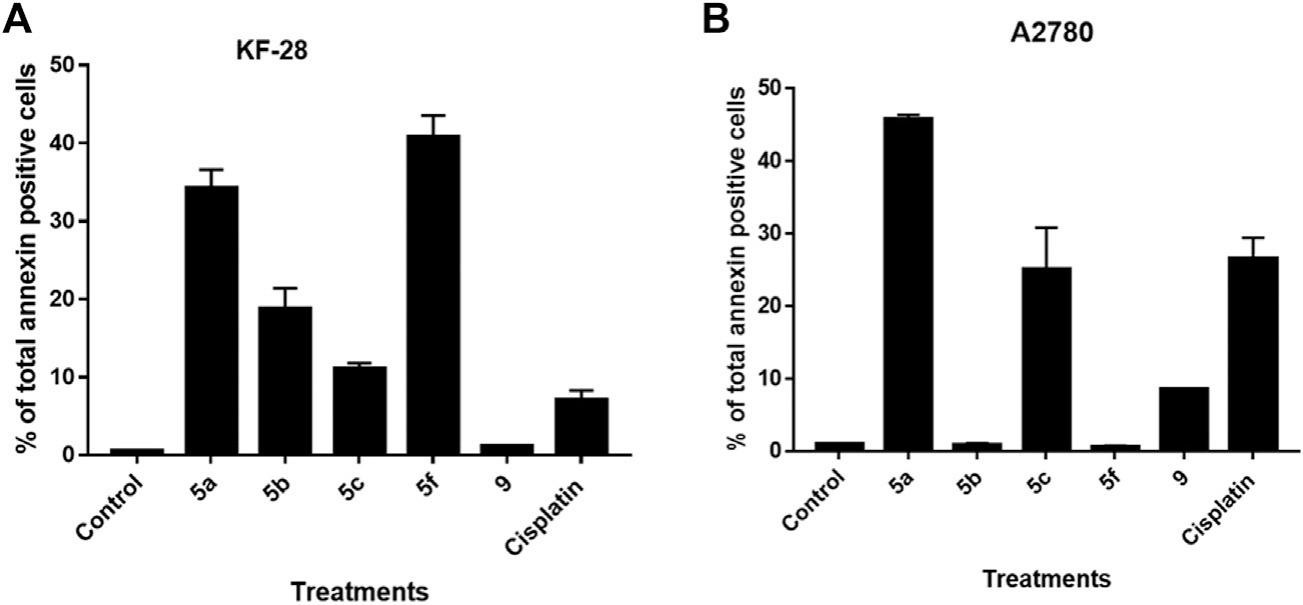
Some compounds induce apoptosis in KF-28 and A2780 cells. Average percentage of annexin-positive cells from KF-28 **(A)** and A2780 **(B)** cells treated with DMSO and different compounds, 5a, 5b, 5c, 5f, and 9, in parallel with the standard drug, cisplatin (10 μM), treatment for 48 h. Values are presented as mean ± SE.

Although the DNA content data showed sub-G0 populations indicating debris as dead cells under treatment with different compounds, such an experiment cannot determine whether this cell death is apoptosis or necrosis. Notably, annexin V/PI staining is an important technique to discriminate the apoptotic cell death from the necrotic one. Therefore, we decided to use such technique to investigate if the cytotoxic effect of the compounds indicated by MTT and the cell cycle experiments is apoptotic cell death or not. For that purpose, two cell lines, KF-28 and A2780, were stained by annexin V/PI after treatment with different compounds vs. DMSO for two days in parallel with cisplatin as a reference drug. Results from annexin staining analysis show appreciated annexin V–stained populations when cells are treated with compounds 5a–c, 5f, and 9. As shown in Figure 2A, the tested compounds, especially 5a, 5b, and 5f, significantly stimulated total apoptotic cell death valued as 38.21, 18.77, and 40.74%, respectively, compared to 0.45% for DMSO-treated controls, in parallel with cisplatin which induced total apoptosis of 25% in KF-28 cells. Similar results were obtained from the other cell line, A2780, treated with the same compounds (Figure 2B).

Our results of the apoptosis induction in the treated KF-28 cells were in accordance with previous research data (Bachmann et al., 2004; Bachmann et al., 2006) which proved the apoptotic activity of the *bis*-thiazole derivatives in some tested cell lines and confirmed apoptosis as the mechanism of action. Moreover, our data revealed that compound 9 did not induce appreciated apoptosis. Together, this observation with the MTT results indicates that compound 9 is affecting the cellular proliferation rather than killing the cells by apoptosis or necrosis.

##### Gene Expression Analysis of Apoptosis-Related Genes

Next, we investigated the pathway through which apoptosis is induced by compounds 5a, 5b, and 5f in KF-28 cells. For that purpose, after treating cells with each compound, RNA was extracted, cDNA was synthesized, and RT-PCR was performed to quantify the mRNA expression of pro-apoptotic genes (p53, bax, puma), caspases 3, 8 9, anti-apoptotic gene (Bcl-2), and Pim-1 kinase genes in KF-28 cells.

**FIGURE 3:**
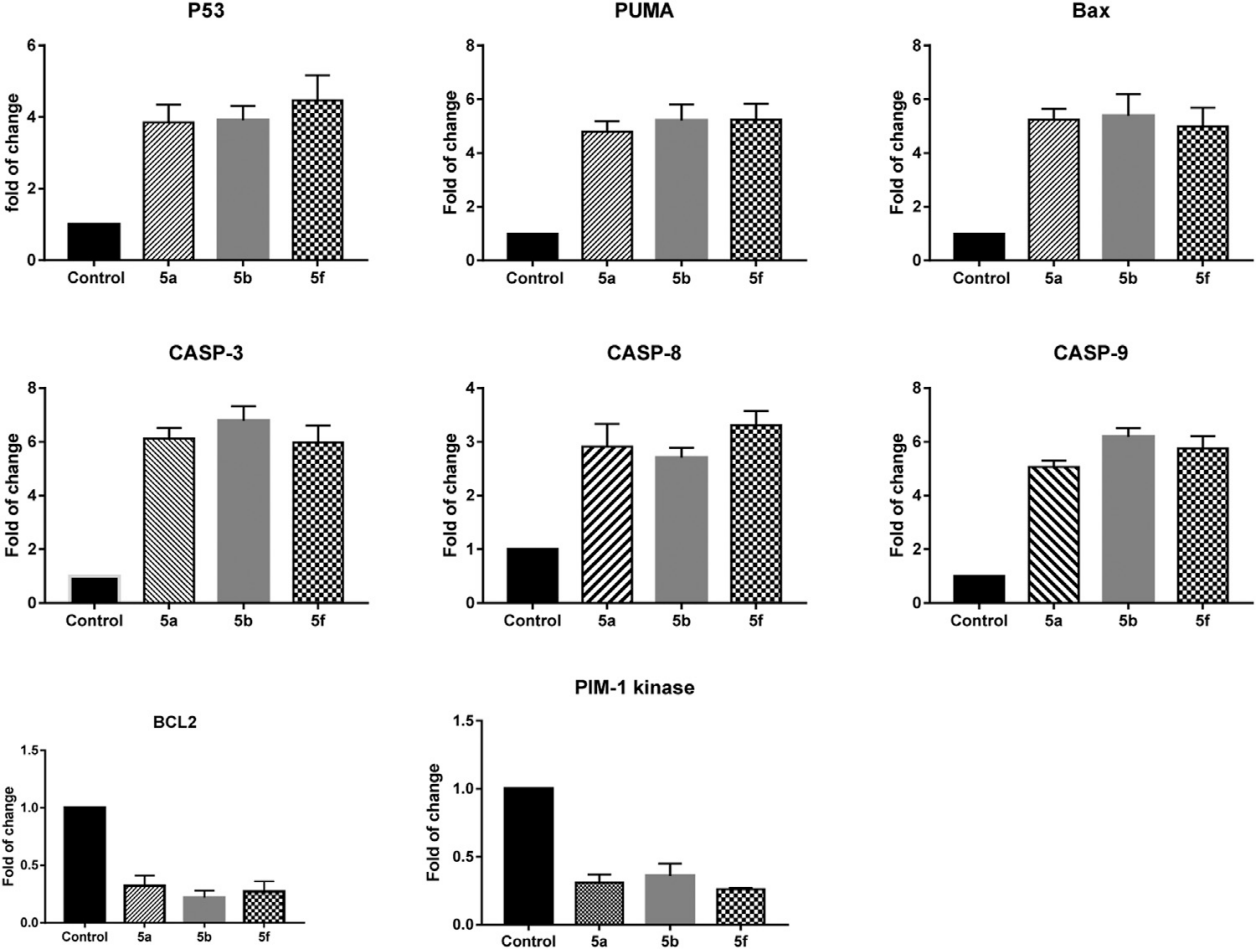
Quantitative RT-PCR result analysis of the apoptosis-related genes: p53, bax, puma, caspases 3, 8, and 9, Bcl-2, and Pim-1 kinase, respectively, in KF-28 cells treated with some selected compounds 5a, 5b, and 5f for 48 h of incubation.

As shown in Figure 3, treatment of KF-28 cells with compound **5a** resulted in a remarkable activation of p53 mRNA relative expression (≈4.07-fold) with a concomitant activation of the puma and bax mRNA levels with a maximum increase of ≈5.08-fold and 5.2-fold, respectively. Compound 5a was able to significantly increase the mRNA levels of caspase 3, 8, 9 genes with a maximum increase of ≈6.2-fold, 2.9-fold, and 5.6-fold, respectively, while it exceptionally inhibited the anti-apoptotic Bcl-2 expression (maximum decrease of ≈0.27-fold) and the Pim-1 kinase expression (maximum decrease of ≈0.31-fold). Additionally, treatment of KF-28 cells with compound 5b significantly activated the level of p53 expression (≈3.41-fold) with consequent activation of the puma and bax expression levels with a maximum increase of ≈4.14-fold and 3.33-fold, respectively, as shown in Figure 3. The *bis*-thiazole derivative 5a was also able to significantly increase the mRNA levels of caspase 3, 8, 9 genes with a maximum increase of ≈4.2-fold, 2.9-fold, and 4.7-fold, respectively. At the same time, it significantly inhibited the anti-apoptotic Bcl-2 expression (maximum decrease of ≈0.5-fold) and the Pim-1 kinase expression (maximum decrease of ≈0.48-fold). Finally, in the same Figure 3, treatment of KF-28 cells with compound 5f significantly activated the level of p53 gene (≈3.81-fold) with concomitant activation of the puma and bax expression levels with a maximum increase of ≈3.14-fold and 2.33-fold, respectively. Compound 5f was able to effectively increase the mRNA levels of caspase 3, 8, 9 gene expression with a maximum increase of ≈6.9-fold, 5.5-fold, and 6.0-fold, respectively, while it notably inhibited the anti-apoptotic Bcl-2 gene (maximum decrease of ≈0.76-fold) and the Pim-1 kinase mRNA expression (maximum decrease of ≈0.86-fold).

Based on the fact that cancer is known as one of the scenarios where too little apoptosis occurs (Wong, 2011), testing the ability of novel drugs for their anti-cancer potency depends mainly on their ability to induce apoptosis. Our data revealed that some of the tested *bis*-thiazole derivatives used here (5a, 5b, and 5f) were able to induce apoptosis in the representative cancer cell lines. This apoptosis is mainly a mitochondrial-dependent one as indicated by the gene expression experiment. These compounds were able to enhance the pro-apoptotic genes and inhibit the anti-apoptotic ones, transcriptionally.

Pim-1 kinase has been observed to be highly expressed in some solid tumors (Dhanasekaran et al., 2001; Li et al., 2006). Therefore, targeting such molecules may associate with tumor growth inhibition. Our data revealed that the investigated compounds, here, were able to, transcriptionally, inhibit Pim-1. Together, our data are in accordance with those from other research teams (Xia et al., 2009; Zhao et al., 2016; Bataille et al., 2017), who reported that thiazole compounds can target Pim-1 kinase.

#### Molecular Docking Simulation

There is a lot of research highlighting molecular docking simulations for the anti-cancer activity of thiazole-based derivatives through Pim-1 kinase (Xia et al., 2009; Zhao et al., 2016; Bataille et al., 2017). Therefore, the binding affinities of the tested compounds were screened toward Pim-1 kinase proteins in terms of binding energies and ligand–receptor interactions. Thus, molecular docking simulation of the tested *bis*-thiazole derivatives toward Pim-1 kinase (PDB = 2OBJ) was investigated at the gene expression level using the RT-PCR in the treated FK-28 cells with compounds 5a, 5b, and 5f as mentioned above (Figure 3).

**FIGURE 4:**
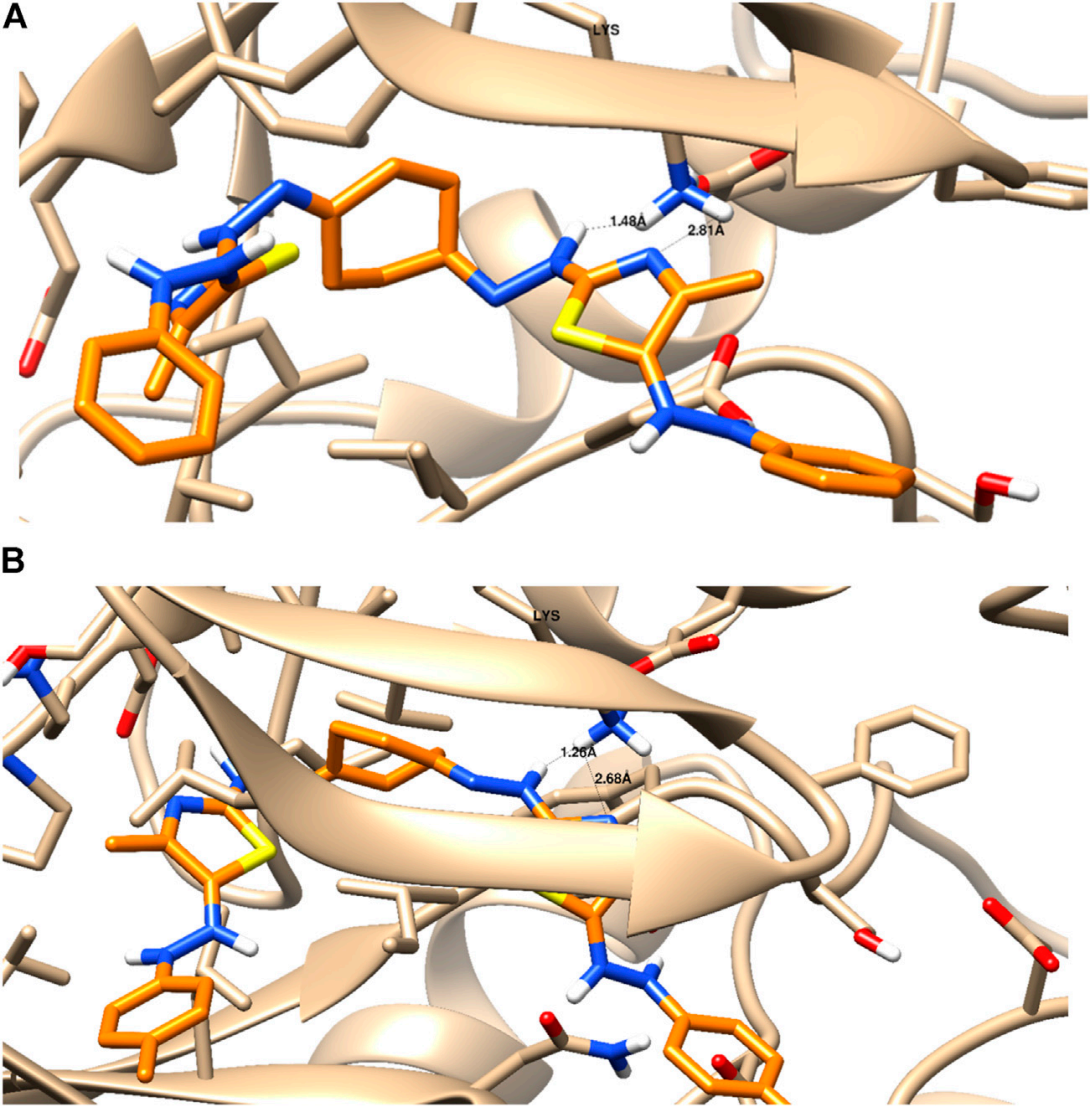
Binding disposition and interaction analysis of the docked compounds 5a **(A)** and 5b **(B)**, inside the Pim-1 kinase protein.

As seen in Table 2 and Figure 4, compounds 5a and 5b were docked to form two hydrogen bonds (HBs) with the interactive key amino acid residue (Lys 67) with binding energies −11.46 and −12.66 kcal/mol, respectively, compared to the standard Pim-1 kinase inhibitor (AZD-1208), which formed only one HB with the same amino acid Lys 67. All HBs were formed through their nitrogen as HBAs with Lys 67, which is the important amino acid for the biological activity of Pim-1 kinase protein. Other interactions of the two compounds 5a and 5b with the receptor-binding site are arene–arene interactions or HBs with amino acid residues other than Lys 67 which are not important for biological activity but are important for fitting the ligands inside the receptor pocket. The docking studies indicated that both compounds 5a and 5b showed promising binding activity as Pim-1 kinase inhibitors following the RT-PCR results in the treated KF-28 cells in which gene expression levels of Pim-1 kinase were inhibited. This may be the proposed anti-breast cancer mode of action.

**TABLE 2:** Ligand–receptor interactions with the binding energy of the docked derivatives^**#**^ inside Pim-1 kinase (PDB = 2OBJ) using MOE 2015^a^.

Docked compound	Binding energy (kcal/mol)	Binding interactions			
		H-bonding with key amino acid (Lys 67)			Other interactions
		No.	Interactive moiety	Length (^◦^A)	
5a	−11.46	2	-N- as HBA	1.43	One arene–cation interaction with Lys 169
			-N- as HBA	1.26	One HB as HBD with Asp 186
					Lipophilic interactions with lipophilic (greasy) amino acids
5b	−12.66	2	-N- as HBA	2.01	One arene–cation interaction with Lys 169 One HB as HBD with Asp 186 Lipophilic interactions with lipophilic (greasy) amino acids
			-N- as HBA	1.76	
AZD-1208 (CAS Number:	−13.42	1	-N- as HBA	1.78	One hydrogen bond donor with Asp 186
1204144-28-4)					Lipophilic interactions with lipophilic (greasy) amino acids

Two-dimensional interactions of docked compounds **5a** (**A**), **5b** (**B**), and standard Pim-1 inhibitor AZD-1208 (**C**).

a RMDS value for self-docking = 0.73 (lower than 2), which validates the MOE docking calculation for the tested compounds. ^#^The docking results for the other tested compounds are supported as supplementary.

#### Structure–Activity Relationship

**FIGURE 5:**
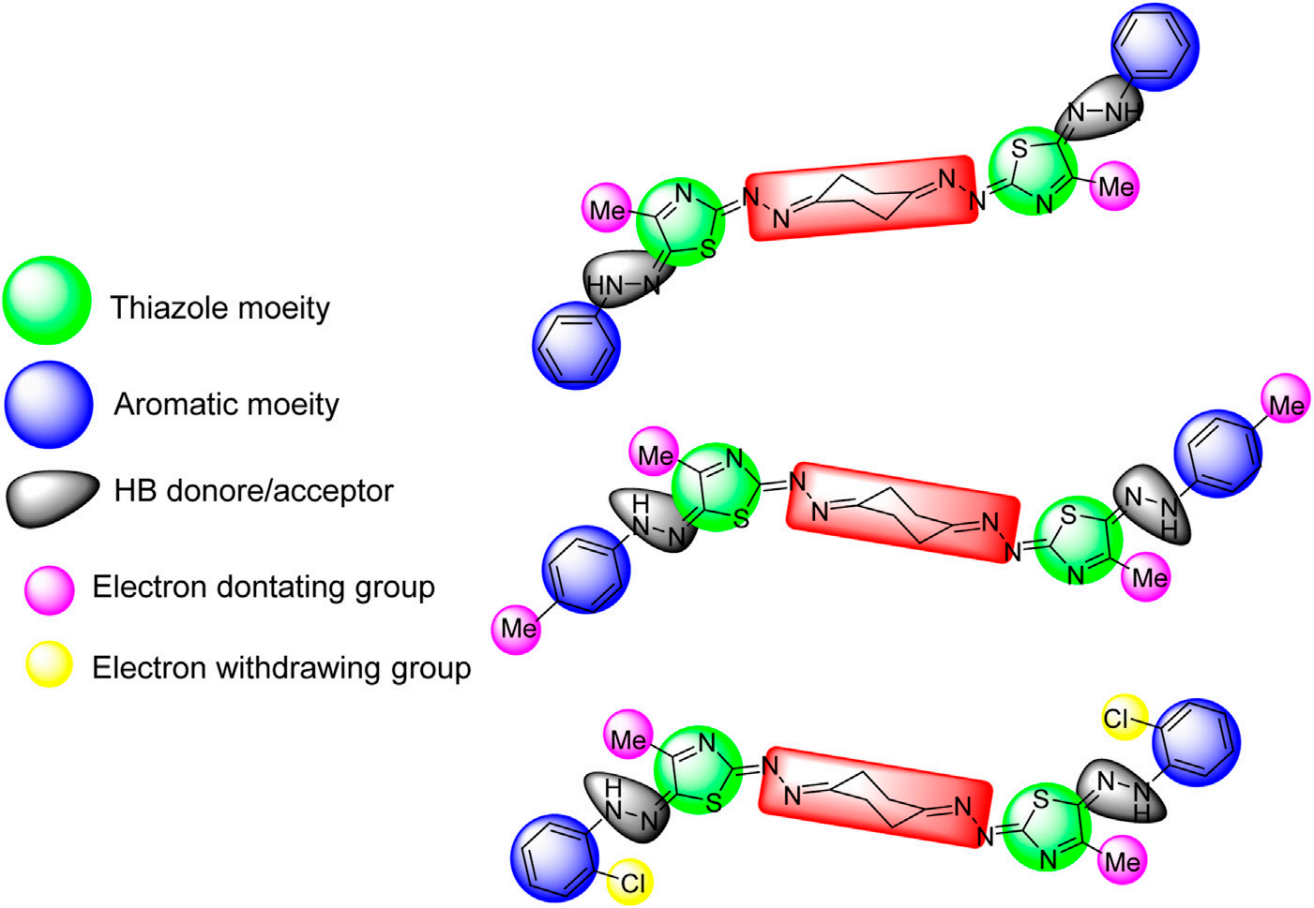
Highlighted pharmacophoric regions for the active compounds 5a, 5b, and 5f, respectively (top to down).

The three active compounds 5a, 5b, and 5f against KF-28 with IC_50_ values 0.718, 3.37, and 0.006 μM, respectively, were highlighted with the common pharmacophoric regions including the thiazole moiety, aromatic moiety, and hydrogen bond donor/acceptor. Interestingly, compound 5f was found to be the most cytotoxic one with IC_50_ value 6 nM. This may be greatly affected by the presence of two withdrawing groups (Cl), as shown in Figure 5. The highlighted pharmacophoric regions to which the biological activity may be incorporated are the thiazole moiety, aromatic (phenyl) moiety, HB donor/acceptor, and electron-donating/withdrawing groups.

#### C-myc Is Negatively Affected by Some Substrates

**FIGURE 6:**
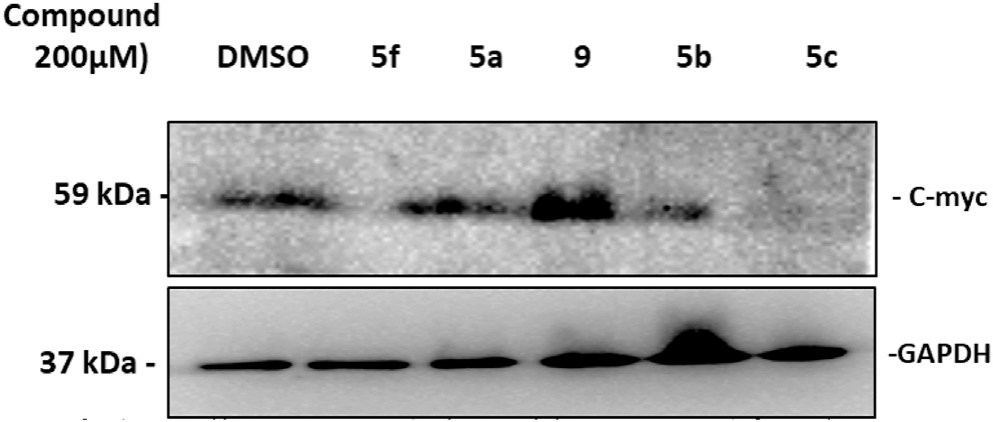
Western blot of C-myc expression in KF-28 cells treated either with DMSO or compounds 5a–c, 5f, and 9 for two days. The cell lysates were developed and fractionated by 15% SDS-PAGE, blotted, and probed with anti-C-myc Ab to show its relative expression. Appreciated downregulation of C-myc expression under treatment with 5f, 5b, and 5c is shown. The lower band is GAPDH which was used as a loading control.

Pim-1 has been classified as a weak oncogene. However, a very strong synergism with regard to tumorigenicity occurs between Pim-1 and C-myc. This synergism is observed with bitransgenic mice overexpressing C-myc and Pim-1 in lymphoid tissue (Möröy et al., 1991). To further validate the simulation and docking data which introduced Pim-1 kinase as a potential target molecule for some of the tested compounds, we decided to check the effect of treatment with the selected compounds on the C-myc protein expression as a downstream target molecule known to be phosphorylated and stabilized by Pim-1 as a kinase (Zhang et al., 2008). Western blotting data show that treating KF-28 cells with the compounds 5a–c, 5f, and 9 for two days reflected observable downregulation of the overall C-myc protein under such treatment with 5a, 5b, 5c, and 5f (Figure 6). Taking into consideration the fact that Pim-1 phosphorylates C-myc and controls its stability (Zhang et al., 2008) may conclude that the reduction of C-myc expression under the indicated compounds is due to the inhibition of Pim-1 activity (Figure 3). Therefore, the speculated inhibition of Pim-1 is supported by the above-mentioned qPCR data (Figure 3) and further supported by C-myc downregulation. Our results agree with the reported one which states that some thiazole derivatives are potent inhibitors against protein kinases including Pim-1 kinase (Mohareb et al., 2019).

## Materials and Methods

### Chemistry

#### General Methods

All melting points are uncorrected. Compounds prepared by different procedures were characterized by mixed melting points, TLC, and IR. IR spectra (KBr) were recorded on a Fourier transform infrared spectrophotometer (IRAffinity-1S from Shimadzu Corporation). NMR spectra were measured with a Varian Gemini 300 spectrometer (300 MHz ^1^H NMR), and chemical shifts are given in ppm from TMS. ^13^C NMR spectra were recorded with a Varian Mercury 300 spectrometer (300 MHz ^1^H NMR, 75 MHz ^13^C NMR). The ^1^H NMR samples were measured in 5 mm NMR tubes with a concentration of about 20 mg of the product in 0.6 ml of solvent (DMSO-*d*_*6*_) and about 75–100 mg of the product in about 0.6 ml of solvent for ^13^C NMR. Mass spectra were recorded on DI Analysis Shimadzu QP-2010 Plus. Elemental analyses were carried out at the Microanalytical Center, Cairo University. 1,4-Cyclohexanedione, ethyl chloroacetate, chloroacetic acid, ethyl bromoacetate, α-chloroacetylacetone, and thiosemicarbazide were used as purchased from Aldrich. The starting materials 4a–g were prepared as reported in the literature (Eweiss and Osman, 1980; Farag and Algarib, 1988).

#### Synthesis of 1,4-Cyclohexane-bis-thiosemicarbazone (3)

To a solution of 1,4-cyclohexanedione (1) (0.11 g, 1 mmol) in absolute ethanol (20 ml), thiosemicarbazide (2) (0.182 g, 2 mmol) was added, and then the reaction mixture was heated under reflux for 10 min with stirring. The reaction mixture was then left to cool to room temperature, and the solid product was collected by filtration, washed with ethanol, dried, and finally recrystallized from ethanol/DMF to give compound 3 as colorless crystals, 0.18 g (65%), mp. 213–214°C [Lit. mp. 213–214°C (Ceba et al., 1982)]; IR (KBr) υ 3,255, 3,201, 3,185 (NH, NH_2_), 1,597 (C=N) cm^−1^; ^1^H NMR (DMSO-*d*_*6*_) *δ* 2.42–2.50 (m, 4H, cyclohexane-H’s), 2.54–2.74 (m, 4H, cyclohexane-H’s), 7.59 (br. s, 2H, NH_2_), 8.05 (br. s, 2H, NH_2_), 9.9–10.11 (br. s, 2H, NH).

#### Synthesis of 1,4-Bis((5-arylazo-4-methylthiazol-2-ylidene)hydrazono)cyclohexanes (5a–g) (Method A): General Procedure

To a mixture of 1,4-cyclohexane-*bis*-thiosemicarbazone (3) (0.258 g, 1 mmol) and the appropriate hydrazonoyl chlorides 4a–g (2 mmol) in absolute ethanol (10 ml), triethylamine (0.1 ml) was added, and the resulting mixture was refluxed for 6–8 h and then left to cool to room temperature. The solid product formed was collected by filtration, washed with ethanol, dried, and finally recrystallized from the ethanol/DMF mixture to give the corresponding *bis*-thiazole derivatives 5a–g.

#### Method B (for Compounds 5a–c)

To a mixture of 1,4-cyclohexadione (1) (0.11 g, 1 mmol), thiosemicarbazide (2) (0.182 g, 2 mmol), and the appropriate hydrazonoyl chlorides 4a–c (2 mmol) in absolute ethanol (10 ml), triethylamine (0.1 ml) was added, and then the reaction mixture was heated under reflux for 8 h. The reaction mixture was then left to cool to room temperature for complete precipitation. The solid product was collected by filtration, washed with ethanol, dried, and finally recrystallized from the ethanol/DMF mixture to afford the *bis*-thiazole derivatives 5a, 5b, and 5c in 77, 76, and 60% yields, respectively.

*1,4-Bis*((*5-phenylazo-4-methylthiazol-2-ylidene*)*hydrazono*)*cyclohexane* (5a)*:* Brown crystals, yield (0.45 g, 83%), mp. 211–212 °C; IR (KBr) ν 3,229 (NH), 1,600 (C=N) cm^−1^; MS *m/z* (%): 77 (100%), 93 (71.2%), 108 (31%), 218 (40%), 542 (M^+^, 1.4%); ^1^H NMR (DMSO-d_6_) *δ* 2.56 (s, 6H, 2CH_3_), 2.73–2.75 (m, 4H, cyclohexane-H’s), 2.89–2.91 (m, 4H, cyclohexane-H’s), 6.9–7.33 (m, 10H, ArH), 10.44 (s, 2H, 2NH). Anal. calcd. for C_26_H_26_N_10_S_2_ (542.68): C, 57.54; H, 4.83; N, 25.81%. Found: C, 57.24; H, 4.64; N, 25.59%.

*1,4-Bis*((*5-(4-tolylazo)-4-methylthiazol-2-ylidene*)*hydrazono*)*cyclohexane* (5b)*:* Brown crystals, yield (0.25 g, 87%), mp. 221–222 °C; IR (KBr) ν 3,297 (NH), 1,615 (C=N) cm^−1^; MS m/z (%): 91 (100%), 106 (53%), 232 (28%), 570 (M^+^, 1%); ^1^H NMR (DMSO-d_6_) *δ* 2.25 (s, 6H, 2CH_3_-Ar), 2.51 (s, 6H, 2CH_3_-thiazole), 2.73–2.74 (m, 4H, cyclohexane-H’s), 2.89–2.90 (m, 4H, cyclohexane-H’s), 7.12 (d, 4H, *J* = 8.4 Hz, ArH), 7.23 (d, 4H, *J* = 8.1 Hz, ArH), 10.41 (s, 2H, 2NH); ^13^C NMR (DMSO-d_6_) *δ* 16.53, 20.49, 25.96, 30.88, 32.25, 35.89, 114.25, 120.70, 129.83, 131.19, 141.25, 162.42, 171.01, 177.69. Anal. calcd. for C_28_H_30_N_10_S_2_ (570.74): C, 58.93; H, 5.30; N, 24.54%. Found: C, 58.72; H, 5.10; N, 24.34%.

*1,4-Bis*((*5-(4-chlorophenylazo)-4-methylthiazol-2-ylidene*)*hydrazono*)*cyclohexane* (5c)*:* Brown crystals, yield (0.33 g, 54%), mp. 269–270 °C; IR (KBr) ν 3,250 (NH), 1,620 (C=N) cm^−1^; MS m/z (%): 92 (82%), 111 (100%), 252 (42%), 611 (M^+^, 2%); ^1^H NMR (DMSO-d_6_) *δ* 2.5 (s, 6H, 2CH_3_), 2.73–2.75 (m, 4H, cyclohexane-H’s), 2.9 (m, 4H, cyclohexane-H’s), 7.30–7.37 (m, 8H, ArH), 10.50 (s, 2H, 2NH). Anal. calcd. for C_26_H_24_Cl_2_N_10_S_2_ (611.57): C, 51.06; H, 3.96; N, 22.90%. Found: C, 50.89; H, 3.76; N, 22.65%.

*1,4-Bis*((*5-(4-nitrophenylazo)-4-methylthiazol-2-ylidene*)*hydrazono*)*cyclohexane* (5d)*:* Green crystals, yield (0.42 g, 66%), mp. >300 °C; IR (KBr) ν 3,271 (NH), 1,627 (C=N) cm^−1^; MS m/z (%): 55 (100%), 71 (64%), 263 (19.4%), 632 (M^+^, 5%); ^1^H NMR (DMSO-d_6_) *δ* 2.59 (s, 6H, 2CH_3_), 2.77 (s, 4H, cyclohexane-H’s), 2.92 (s, 4H, cyclohexane-H’s), 7.44 (d, 4H, J = 7.8 Hz, ArH), 8.19 (d, 4H, J = 8.1 Hz, ArH), 10.98 (s, 2H, 2NH). Anal. calcd. for C_26_H_24_N_12_O_4_S_2_ (632.68): C, 49.36; H, 3.82; N, 26.57%. Found: C, 49.06; H, 3.62; N, 26.36%.

*1,4-Bis*((*5-(2-tolylazo)-4-methylthiazol-2-ylidene*)*hydrazono*)*cyclohexane* (5e)*:* Brown crystals, yield (0.45 g, 78%), mp. 224–225 °C; IR (KBr) ν 3,200 (NH), 1,624 (C=N) cm^−1^; MS m/z (%): 65 (33%), 77 (19.4%), 91 (100%), 106 (42%), 232 (4%), 339 (4%), 452 (2%), 542 (3%), 570 (M^+^, 1%); ^1^H NMR (DMSO-d_6_) *δ* 2.34 (s, 6H, 2CH_3_-Ar), 2.55 (s, 6H, 2CH_3_-thiazole), 2.75 (s, 4H, cyclohexane-H’s), 2.9 (m, 4H, cyclohexane-H’s), 6.94–7.43 (m, 8H, ArH), 9.39 (s, 2H, 2NH). Anal. calcd. for C_28_H_30_N_10_S_2_ (570.74): C, 58.93; H, 5.30; N, 24.54%. Found: C, 58.63; H, 5.27; N, 24.34%.

*1,4-Bis*((*5-(2-chlorophenylazo)-4-methylthiazol-2-ylidene*)*hydrazono*)*cyclohexane* (5f)*:* Brown crystals, yield (0.3 g, 75%), mp. >300 °C; IR (KBr) ν 3,270 (NH), 1,620 (C=N) cm^−1^; MS m/z (%): 92 (54%), 127 (100%), 217 (12%), 359 (1.3%), 611 (M^+^, 1.5%); ^1^H NMR (DMSO-d_6_) *δ* 2.58 (s, 6H, 2CH_3_), 2.73–2.77 (m, 4H, cyclohexane-H’s), 2.91 (m, 4H, cyclohexane-H’s), 7.06 (t, 2H, J = 6.6 Hz, ArH), 7.37 (t, 2H, J = 7.51 Hz, ArH), 7.45 (d, 2H, J = 7.2 Hz, ArH), 7.59 (d, 2H, J = 8.1 Hz, ArH), 9.33 (s, 2H, 2NH). Anal. calcd. for C_26_H_24_Cl_2_N_10_S_2_ (611.57): C, 51.06; H, 3.96; N, 22.90%. Found: C, 50.98; H, 3.76; N, 22.70%.

*1,4-Bis*((*5-(2-nitrophenylazo)-4-methylthiazol-2-ylidene*)*hydrazono*)*cyclohexane* (5g): Brown crystals, yield (0.20 g, 51%), mp. 220–221 °C; IR (KBr) ν 3,379 (NH), 1,651 (C=N) (cm^−1^); MS m/z (%): 71 (100%), 92 (57%), 230 (19.4%), 263 (49%), 372 (3%), 539 (5%), 630 (M^+^-2, 3%); ^1^H NMR (DMSO-d_6_) *δ* 2.58 (s, 6H, 2CH_3_), 2.73–2.76 (m, 4H, cyclohexane-H’s), 2.88–2.91 (m, 4H, cyclohexane-H’s), 7.55–8.05 (m, 8H, ArH), 10.73 (s, 2H, 2NH). Anal. calcd. for C_26_H_24_N_12_O_4_S_2_ (632.68): C, 49.36; H, 3.82; N, 26.57%. Found: C, 49.16; H, 3.63; N, 26.38%.

#### Reaction of Bis-thiosemicarbazone 3 With α-Chlorocarbonyl Compounds 6–8 and 10: General Procedure

(A) To a mixture of 1,4-cyclohexane-*bis*-thiosemicarbazone (3) (0.26 g, 1 mmol) and 2 mmol of the appropriate α-halocarbonyl derivatives [ethyl chloroacetate (6), chloroacetic acid (7), and ethyl bromoacetate (8)] in a 1:1 DMF/ethanol mixture (10 ml), triethylamine (0.1 ml) was added, and the resulting mixture was heated at reflux temperature for 8 h and then left to cool to room temperature. The solid product obtained was collected by filtration, washed with ethanol, dried, and finally recrystallized from the ethanol/DMF mixture to give compound 9. Yields of compound 9 obtained from α-chlorocarbonyls 6, 7, and 8 were (0.25 g, 73%), (0.21 g, 62%), and (0.23 g, 68%), respectively.
(B) Reaction of 1,4-cyclohexane-bis-thiosemicarbazone (3) (0.26 g, 1 mmol) and α-chloroacetylacetone (10) (2 mmol) was carried out under typical reaction conditions as in procedure (A) to give compound 11 in 71% yield (0.15 g).

*1,4-Bis*((*4-oxo-thiazolidin-2-ylidene*)*hydrazono*)*cyclohexane (*9*):* Yellow crystals, yield (0.25 g, 73%), mp. 279–280 °C; IR (KBr) ν 3,320 (NH), 1712 (C=O), 1,638 (C=N) cm^−1^; MS m/z (%): 87 (40%), 107 (100%), 223 (11%), 338 (M^+^, 2%); ^1^H NMR (DMSO-d_6_) *δ* 2.53–2.57 (m, 4H, cyclohexane-H’s), 2.68–2.73 (m, 4H, cyclohexane-H’s), 3.81 (s, 4H, 2CH_2_CO), 11.72 (s, 2H, 2NH). ^13^C NMR (DMSO-d_6_) *δ* 25.35, 32.23, 32.72, 166, 173.93. Anal. calcd. for C_12_H_14_N_6_O_2_S_2_ (338.40): C, 42.59; H, 4.17; N, 24.83%. Found: C, 42.30; H, 4.42; N, 24.63%.

*1,4-Bis*((*5-acetyl-4-methylthiazol-2-ylidene*)*hydrazono*)*cyclohexane* (***11***)*:* Yellow crystals, yield (0.15 g, 71%), mp. 280–281 °C; IR (KBr) ν 3,194 (NH), 1,695 (C=O), 1,597 (C=N) cm^−1^; MS m/z (%): 107 (83%), 113 (77%), 141 (100%), 156 (49%), 233 (24%), 263 (90%), 418 (M^+^, 8%); ^1^H NMR (DMSO-d_6_) *δ* 3.36 (s, 6H, 2CH_3_), 4.47 (s, 6H, CH_3_CO), 2.55–2.65 (m, 8H, cyclohexane-H’s), 11.4 (br. s, 2H, NH). Anal. calcd. for C_18_H_22_N_6_O_2_S_2_ (418.53): C, 51.66; H, 5.30; N, 20.8%. Found: C, 51.89; H, 5.22; N, 21.03%.

#### Reaction of Cyclohexane-1,4-bis-thiosemicarbazone (3) With α-Bromoketones 12a–d: General Procedure

To a mixture of cyclohexane-1,4-*bis*-thiosemicarbazone (3) (0.26 g, 1 mmol) and the appropriate α-bromoketone derivatives 12a–d (2 mmol) in absolute ethanol (10 ml), triethylamine (0.1 ml) was added, and the resulting mixture was refluxed for 8 h and then left to cool to room temperature. The solid product was collected by filtration, washed with ethanol, dried, and finally recrystallized from the ethanol/DMF mixture to give the corresponding *bis*-thiazoles 13a–d.

*1,4-Bis*((*4-*(*2-benzothiazolyl*)*thiazol-2-ylidene*)*hydrazono*)*cyclohexane* (13a)*: Green solid, yield (0.31* *g, 72%), mp. >300 °C; IR (KBr) ν 3,250 (NH), 1,635* (C=N) cm^−1^; ^1^H NMR (DMSO-d_6_) *δ* 2.58–2.71 (m, 8H, cyclohexane-H's), 7.43 (t, 2H, J = 7.2 Hz, ArH), 7.53 (t, 2H, *J* = 7.5 Hz, ArH), 7.73 (s, 2H, thiazole-2-CH), 8.01 (d, 2H, *J* = 8.1 Hz, ArH’s), 8.09 (d, 2H, *J* = 7.8 Hz, ArH’s), 11.03 (s, 2H, 2NH) ppm. ^13^C NMR (DMSO-d_6_) *δ* 23.94, 26.18, 29.29, 31.50, 109.32, 122.34, 122.54, 125.15, 126.52, 134.54, 144.65, 153.59, 154.11, 162.79, 170.37 ppm. Anal. calcd. for C_26_H_20_N_8_S_4_ (572.07): C, 54.52; H, 3.52; N, 19.56%. Found: C, 54.42; H, 3.46; N, 19.46%.

*1,4-Bis*((*4-*(*2-benzofuryl*)*thiazol-2-ylidene*)*hydrazono*)*cyclohexane* (13b): Brown powder, yield (0.40 g, 74%), mp. 269–270 °C; IR (KBr) ν 3,325 (NH), 1,612 (C=N) cm^−1^; MS m/z (%): 102 (48%), 145 (25.6%), 216 (100%), 305 (14%), 419 (2%), 461 (4.3%), 538 (M^+^, 4.4%); ^1^H NMR (DMSO-d_6_) *δ* 2.55–2.72 (m, 8H, cyclohexane-H’s), 7.03 (s, 2H, ArH), 7.23–7.32 (m, 6H, ArH), 7.55 (d, 2H, J = 8.1 Hz, ArH), 7.64 (d, 2H, J = 7.8 Hz, ArH), 10.79 (br. s, 2H, 2NH). Anal. calcd. for C_28_H_22_N_6_O_4_S_2_ (538.64): C, 62.44; H, 4.12; N, 15.60%. Found: C, 62.50; H, 4.25; N, 15.33%.

*1,4-Bis*((*4-*(*3-coumarinyl*)*thiazol-2-ylidene*)*hydrazono*)*cyclohexane* (13c): Yellow solid, yield (0.25 g, 83%), mp. 241–242 °C; IR (KBr) ν 3,250 (NH), 1,697 (C=O), 1,620 (C=N) cm^−1^; MS m/z (%): 102 (47%), 244 (100%), 452 (0.7%), 487 (3%), 523 (3%), 593 (M^+^-1, 4%); ^1^H NMR (DMSO-d_6_) *δ* 2.57–2.73 (m, 8H, cyclohexane-H’s), 7.35–7.45 (m, 4H, ArH), 7.62 (t, 2H, J = 7.2 Hz, ArH), 7.71 (s, 2H, CH-thiazole), 7.81 (d, 2H, J = 7.8 Hz, ArH), 8.54 (s, 2H, chromenone-H), 10.75 (s, 2H, 2NH). Anal. calcd. for C_30_H_22_N_6_O_4_S_2_ (594.66): C, 60.59; H, 3.73; N, 14.13%. Found: C, 60.87; H, 3.63; N, 14.08%.

*1,4-Bis*((*4-*(*4-chlorophenyl*)*thiazol-2-ylidene*)*hydrazono*)*cyclohexane* (13d): Green solid, yield (0.4 g, 76%), mp. 230–231 °C; IR (KBr) ν 3,327 (NH), 1,609 (C=N) cm^−1^; MS m/z (%): 89 (48%), 168 (52%), 210 (100%), 408 (2%), 433 (3%), 482 (2%), 527 (M^+^, 1%); ^1^H NMR (DMSO-d_6_) *δ* 2.73 (s, 4H, cyclohexane-H’s), 2.88 (s, 4H, cyclohexane-H’s), 7.29 (s, 2H, thiazole-CH), 7.44 (d, 4H, J = 8.4 Hz, ArH), 7.86 (d, 4H, J = 8.4 Hz, ArH), 10.65 (br. s, 2H, 2NH). Anal. calcd. for C_24_H_20_Cl_2_N_6_S_2_ (527.49): C, 54.65; H, 3.82; N, 15.93%. Found: C, 54.55; H, 3.75; N, 15.89%.

#### Synthesis of 1,4-Bis((5-arylidene-4-oxo-thiazolidin-2-ylidene)hydrazono)cyclohexanes 14a–d: General Procedure

To a mixture of the *bis*-thiazolidinone derivative 9 (0.34 g, 1 mmol) and the appropriate aromatic aldehyde derivatives (2 mmol) in DMF/EtOH (10 ml, 1:1), sodium hydroxide (0.08 g) was added, and the resulting mixture was refluxed for 8 h and then left to cool to room temperature. The solid product obtained was collected by filtration, washed with ethanol, dried, and finally recrystallized from the ethanol/DMF mixture to give 14a–d.

*1,4-Bis*((*5-benzylidene-4-oxo-thiazolidin-2-ylidene*)*hydrazono*)*cyclohexane* (14a): Yellowish-red crystals, yield (0.2 g, 77%), mp. >300 °C; IR (KBr) ν 3,124 (NH), 1,697 (C=O), 1,597 (C=N) cm^−1^; ^1^H NMR (DMSO-d_6_) *δ* 2.66–2.73 (m, 4H, cyclohexane-H’s), 2.79–2.88 (m, 4H, cyclohexane-H’s), 7.07–7.66 (m, 10H, ArH& CH = Ar), 12.2 (br. s, 2H, 2NH). Anal. calcd. for C_26_H_22_N_6_O_2_S_2_ (514.62): C, 60.68; H, 4.31; N, 16.33%. Found: C, 60.59; H, 4.52; N, 16.30%.

*1,4-Bis*((*5-(4-methylbenzylidene)-4-oxo-thiazolidin-2-ylidene*)*hydrazono*)*cyclohexane* (14b): Yellow crystals, yield (0.16 g, 58%), mp. 269–270 °C; IR (KBr) ν 3,232 (NH), 1,690 (C=O), 1,630 (C=N) cm^−1^; MS m/z (%): 107 (44.6%), 148 (100%), 218 (13%), 325 (19%), 514 (2%), 542 (M^+^, 6%); ^1^H NMR (DMSO-d_6_) *δ* 2.36 (s, 6H, CH_3_-Ar), 2.25–2.63 (m, 4H, cyclohexane-H’s), 2.65–2.81 (m, 4H, cyclohexane-H’s), 7.35 (d, 4H, J = 7.5 Hz, ArH), 7.57–7.76 (m, 6H, ArH& CH = Ar), 12.45 (br. s, 2H, 2NH). Anal. calcd. for C_28_H_26_N_6_O_2_S_2_ (542.68): C, 61.97; H, 4.83; N, 15.49%. Found: C, 61.91; H, 4.75; N, 15.39%.

*1,4-Bis*((*5-(4-chlorobenzylidene)-4-oxo-thiazolidin-2-ylidene*)*hydrazono*)*cyclohexane* (14c): *Orange-red crystals, yield (0.15* *g, 51%), mp. > 300°C; IR (KBr)* ν 3,124 (NH), 1,697 (C=O), 1,635 (C=N) cm^−1^; MS m/z (%): 55 (100%), 71 (44%), 139 (32%), 278 (13.4%), 435 (1.5%), 581 (M^+^-2, 2%), 587 (M^+^+4, 2%); ^1^H NMR (DMSO-d_6_) *δ* 2.66 (m, 4H, cyclohexane-H’s), 2.82 (m, 4H, cyclohexane-H’s), 7.56–7.66 (m, 10H, ArH& CH = Ar), 12.5 (br. s, 2H, 2NH). Anal. calcd. for C_26_H_20_Cl_2_N_6_O_2_S_2_ (583.51): C, 53.52; H, 3.45; N, 14.4%. Found: C, 53.45; H, 3.41; N, 14.31%.

*1,4-Bis*((*5-(4-bromobenzylidene)-4-oxo-thiazolidin-2-ylidene*)*hydrazono*)*cyclohexane* (14d): *Orange-red crystals, yield (0.*15 m*, 45%), mp. 290–291°C; IR (KBr) ν 3,210 (NH), 1,697 (C=O), 1,643 (C=N) cm* ^−1^ *; MS m/z (%): 89 (100%), 133 (39%), 214 (8%), 284 (4%), 481 (0.5%), 566 (6%), 673 (M++1, 5%);* ^*1*^ *H NMR (DMSO-d*_*6*_ *) δ* 2.50–2.72 (m, 4H, cyclohexane-H’s), 2.79–2.87 (m, 4H, cyclohexane-H’s), 7.47–7.81 (m, 10H, ArH and CH = Ar), 12.55 (s, 2H, 2NH). Anal. calcd. for C_26_H_20_Br_2_N_6_O_2_S_2_ (672.41): C, 46.44; H, 3.00; N, 12.50%. Found: C, 46.30; H, 3.25; N, 12.25%.

### Biological Part

#### General Part

All biological assays were carried out at Faculty of Science, Port Said University, Egypt, as well as at Department of Chemistry, Faculty of Science, Suez Canal University, Ismailia, Egypt**.**

#### Cell Culture, Treatment, and MTT Assay

The human ovarian serous carcinoma cell line KF-28 was kindly provided by Prof. Yoshihiro Kikuchi (Department of Obstetrics and Gynecology, National Defense Medical College, Saitama, Japan), and A2780 cell line was purchased from the ECACC (ECACC 93112519). The cells were maintained in RPMI-1640 (Sigma-Aldrich, United States). The cervical cancer cell line, Hela cells, breast cancer cell line, MCF-7, and MDA-MB-231 were purchased from the ATCC and cultured in DMEM. Both types of media were supplemented with 2 mM L-glutamine (Lonza, Belgium), 10% FBS (Sigma, St. Louis, MO, United States), and 1% penicillin/streptomycin (Lonza, Belgium). All cells were incubated at 37°C in 5% CO_2_ atmosphere (NuAire). All cancer cell lines were cultured according to the routine tissue culture work as discussed by the Freshney group (Freshney, 2010). The cells were plated at a density of 5,000 cells in triplicates in a 96-well plate. On the next day, the cells were treated with the indicated compound(s) at the indicated concentrations in a final volume of 100 μL media. Cell viability was assessed after 72 h using MTT solution (Promega) (Mosmann, 1983). 20 μL of the reagent was added to each well, the plate was incubated for 3 h, fluorescence was subsequently measured (570 nm) using a plate reader, and then the IC_50_ values were calculated using Excel (Tantawy et al., 2020).

#### Cell Death Investigation

##### Flow Cytometric Analysis of Annexin V/PI Staining and Cell Cycle

The cells were seeded into six-well culture plates (3–5 × 10^5^ cells/well) and incubated overnight at 37°C, under 5% CO_2_. The cells were then treated with indicated compounds for 48 h. Next, media supernatants and the cells were collected and washed with ice-cold PBS. Next, the cells were stained with PI (10 mg/ml) for 30 min and analyzed for DNA contents using flow cytometry analysis using an FL2 (λex/em 535/617 nm) signal detector (ACEA Novocyte™ flow cytometer, ACEA Biosciences, Inc., San Diego, CA, United States). For each sample, 12,000 events are acquired. Cell cycle distribution is calculated using a CytoFLex machine and analyzed by CytExpert software. Flow cytometric methodologies (annexin V/PI staining with cell cycle analyses) are carried out as previously described (Nafie et al., 2020a; Nafie et al., 2020b; Gad et al., 2020).

##### RNA Extraction and Gene Expression

The cells were grown in 10 cm^2^ culture dishes to a monolayer with a confluence of 90% in triplicates. After different treatments, the cells were washed twice with PBS and then pelleted by centrifugation for 5 min at 1800 rpm at 4°C. The cells were lysed by Trizol reagent (Invitrogen), and then total RNA was purified with the phenol/chloroform extraction method. Concentration and purity of RNA samples were measured using NanoDrop. The RNA purity was assessed as OD260/OD230 (≥1.5) using NanoDrop. gDNA was eliminated by DNase. The RNA from three biological samples was then pooled for mRNA analysis, cDNA was synthesized using the *i*Script kit (Bio-Rad, United States) according to the manufacturer’s recommendation, and RT-PCRs consisted of 8.34 µL Fluocycle^®^ II SYBR^®^ (Euroclone, Milan, Italy), 0.5 µL of both 10 µM forward and reverse primers, 1 µL cDNA, and 6.34 µL of H_2_O. All reactions were performed for 35 cycles using the following temperature profile: 95°C for 5 min (initial denaturation), 95°C for 15 min (denaturation), 55°C for 30 min (annealing), and 72°C for 30 min (extension) (Nafie et al., 2020a; Sarhan et al., 2020). Then, the Ct values were collected, with the folds of changes between all the samples. The primers used are listed in Table 3.

**TABLE 3:** Primer sequences in forward and reverse.

Primer	Sequence	
β-Actin	FOR: 5′-GCA​CTC​TTC​CAG​CCT​TCC​TTC​C-3′	REV: 5′-GAG​CCG​CCG​ATC​CAC​ACG-3′
p53	FOR: 5′-CTT​TGA​GGT​GCG​TGT​TTG​TG-3′	REV: 5′-GTG​GTT​TCT​TCT​TTG​GCT​GG-3′
Bcl-2	FOR: 5′-GAG​GAT​TGT​GGC​CTT​CTT​TG-3′	REV: 5′-ACA​GTT​CCA​CAA​AGG​CAT​CC-3′
puma	FOR: 5′-GAG​GAG​GAA​CAG​TGG​GC-3′	REV: 5′-CTA​ATT​GGG​CTC​CAT​CTC​GG-3′
bax	FOR: 5′-TTT​GCT​TCA​GGG​TTT​CAT​CC-3′	REV: 5′-CAG​TTG​AAG​TTG​CCG​TCA​GA-3′
Pim-1 kinase	FOR: 5′-GCA​AAT​AGC​AGC​CTT​TCT​GG-3′	REV: 5′-CCT​AGG​ACC​CCT​GGA​GAG​TC-3′
Caspase 3	FOR: 5′-TGG​CCC​TGA​AAT​ACG​AAG​TC-3′	REV: 5′-GGC​AGT​AGT​CGA​CTC​TGA​AG-3′
Caspase 8	FOR: 5′-AAT​GTT​GGA​GGA​AAG​CAA​T-3′	REV: 5′-CAT​AGT​CGT​TGA​TTA​TCT​TCA​GC-3′
Caspase 9	FOR: 5′-CGA​ACT​AAC​AGG​CAA​GCA​GC-3′	REV: 5′-ACC​TCA​CCA​AAT​CCT​CCA​GAA​C-3′

##### Western Blot

Cell lysates were prepared by lysing cells in RIPA buffer (10 mM Tris (pH 7.4), 150 mM NaCl, 1% Triton X-100, 1% Na deoxycholate), supplemented with protease inhibitor cocktail (Sigma). Protein concentration of whole cell lysates was determined by the BSA assay using the BSA kit (Pierce), and then equal protein amounts were heated to 95 °C for 5 min with sodium dodecyl sulfate (SDS) sample buffer (25 ml glycerol, 31.2 ml Tris buffer, 7.5 ml SDS, a dash of bromophenol blue/100 ml) and run on 15% SDS polyacrylamide gel electrophoresis (SDS-PAGE). Protein samples were then blotted onto PVDF membranes (Immobilon P, Watford, United Kingdom). The membranes were incubated in blocking solution (5% non-fat milk in PBS) for 1 h and then in primary antibody (anti-human C-myc pAb (Abcam; at dilution of 2:1,000) or GAPDH mAb (Santa Cruz; at dilution of 20:1,000)) overnight. After 3 × 10 min washes in TBS (0.1% Tween-20 in PBS), the membrane was incubated for 1 h at room temperature with horseradish-peroxidase–linked (HRP) secondary anti-rabbit or anti-mouse Ab (1:1,000 dilution in PBS). Signals on the membrane were developed using ECL reagent (Amersham, CA, United States) and then were imaged with the Chemidoc system (Bio-Rad, United States).

#### Molecular Docking Studies

For elucidation of the virtual mechanism of binding, the molecular docking study toward the Pim-1 kinase active site was carried out. All synthesized derivatives were chemically and energetically optimized. Additionally, the protein structure (PDB = 2OBJ, https://www.rcsb.org/structure/2OBJ) was also optimized following the routine work as discussed by Poli *et al.* (Poli et al., 2016). MOE 2014 was used as the validated molecular docking calculation, and Chimera software was finally used as the visualized software for the analysis of drug–target interactions.

## Conclusion

In this work, we successfully prepared 1,4-cyclohexanedione-*bis*-thiosemicarbazone and studied its synthetic utility as a key compound for preparing four different series of new 1,4-cyclohexane–based *bis*-thiazole derivatives *via* either stepwise reaction or the one-pot multi-component procedure. In addition, most of the obtained compounds were screened *in vitro* for their cytotoxic activities in five cancer cell lines. Two of the tested compounds (5c and 5f) exhibited pronounced cytotoxic activities with high IC_50_ values 0.6 and 6 nM against the Hela and KF-28 cell lines, respectively. Taken together, the tested compounds exhibited remarkable cytotoxic activities against the tested cancer cell lines with apoptosis induction through inhibition of Pim-1 kinase. Therefore, compounds 5c and 5f are worthy to be further tested *in vivo* for developing chemotherapeutic anti-cancer agents.

## Data Availability

The original contributions presented in the study are included in the article/Supplementary Material, and further inquiries can be directed to the corresponding author.
