# Supplementary material for: Novel Bis-Thiazole Derivatives: Synthesis and Potential Cytotoxic Activity Through Apoptosis With Molecular Docking Approaches
Source: Front Chem. 2021 Aug 12;9:694870. doi: 10.3389/fchem.2021.694870 (PMC8397418; doi:10.3389/fchem.2021.694870)

Supplementary file

**Novel** ***Bis*-Thiazole Derivatives: Synthesis and Potential Cytotoxic Activity Through Apoptosis with Molecular Docking Approaches**

**Kamal M. Dawood,^a,^* Mohamed A. Raslan,^b^ Ashraf A. Abbas,^a^ Belal E. Mohamed,^b^ Magda H. Abdellattif,^c^ Mohamed S. Nafie,^d^ Mohamed K. Hassan,^e,f^**


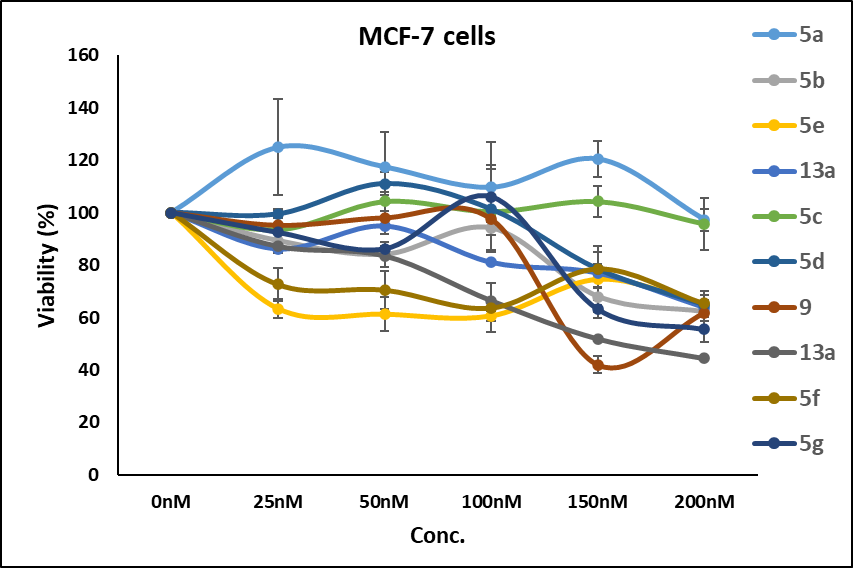

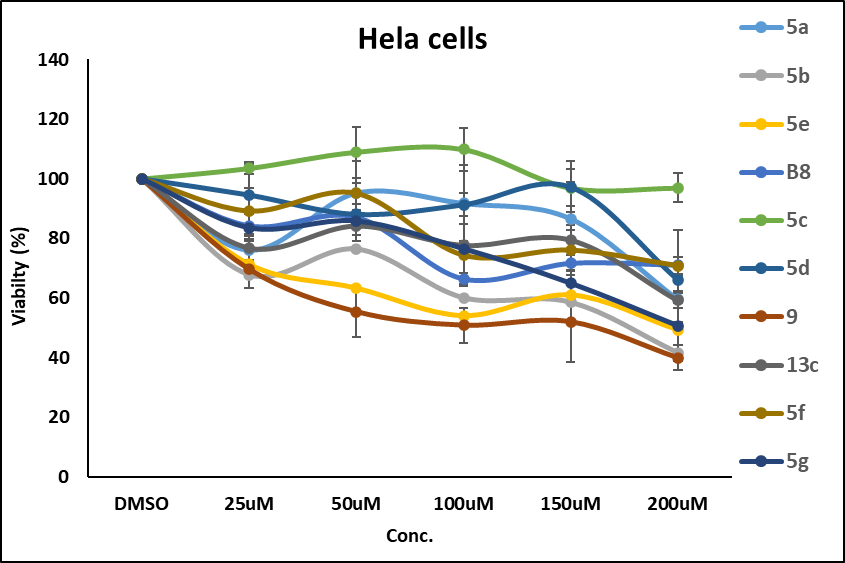


**A**

**B**


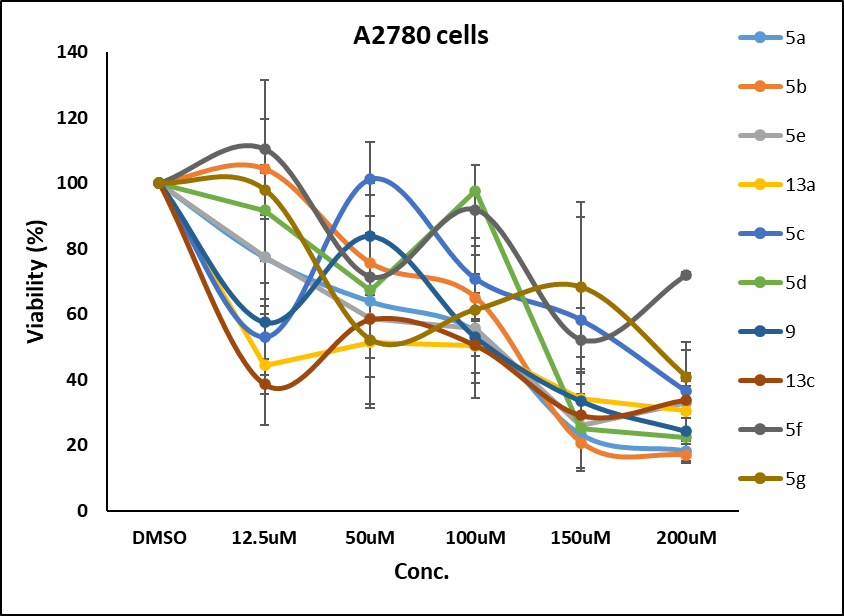


**C**


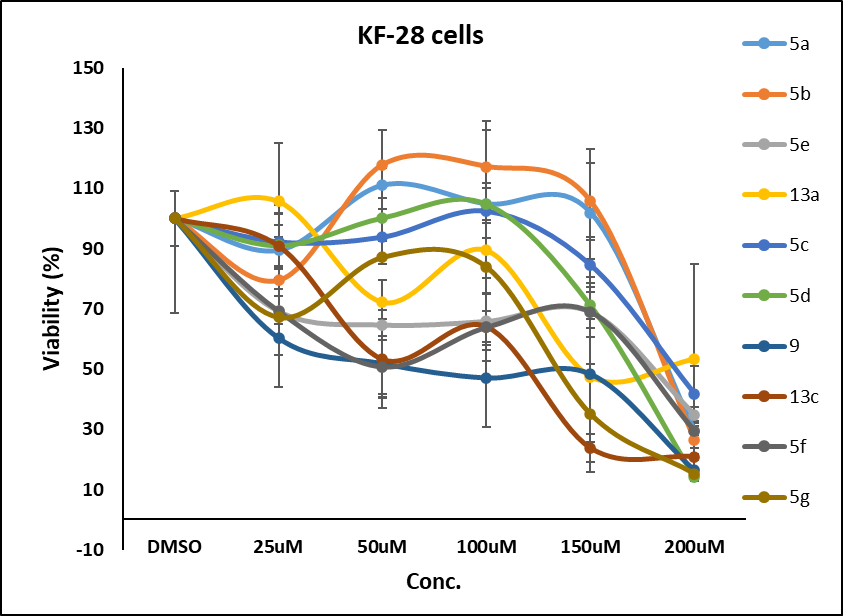


**D**


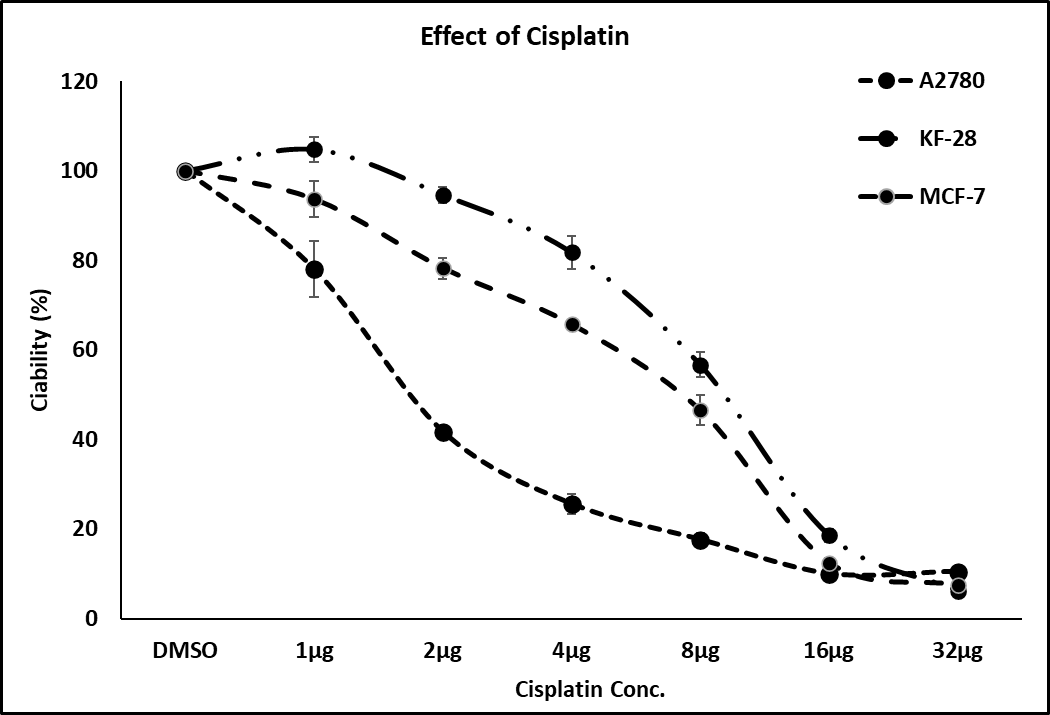

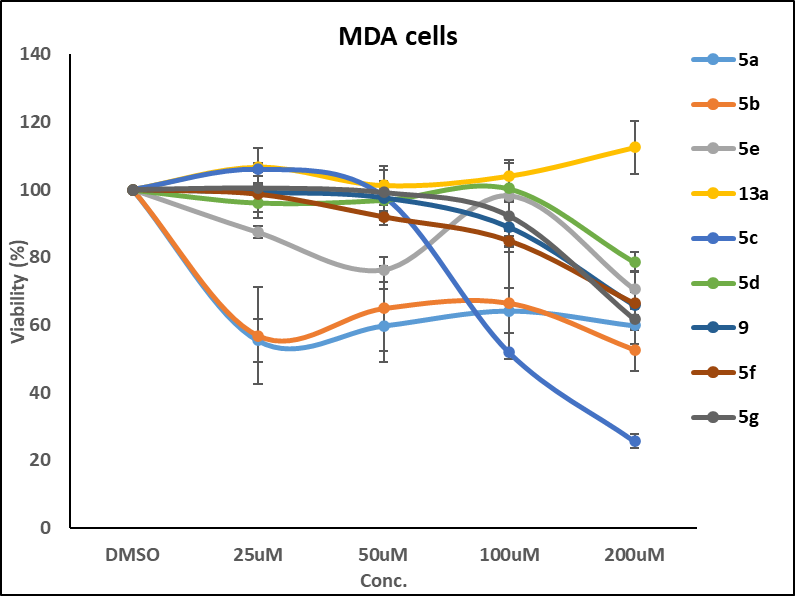


**E**

Figure S1. Percentages of viability for the tested compounds **5a-g, 9** and **13a** against **A**: Hela, **B**: MCF, **C**: A2780, **D**: KF-28, **E**: MDA, and **F**: combined grapgh for the effect of cisplatin as standard drug against the tesetd cell lines

**F**


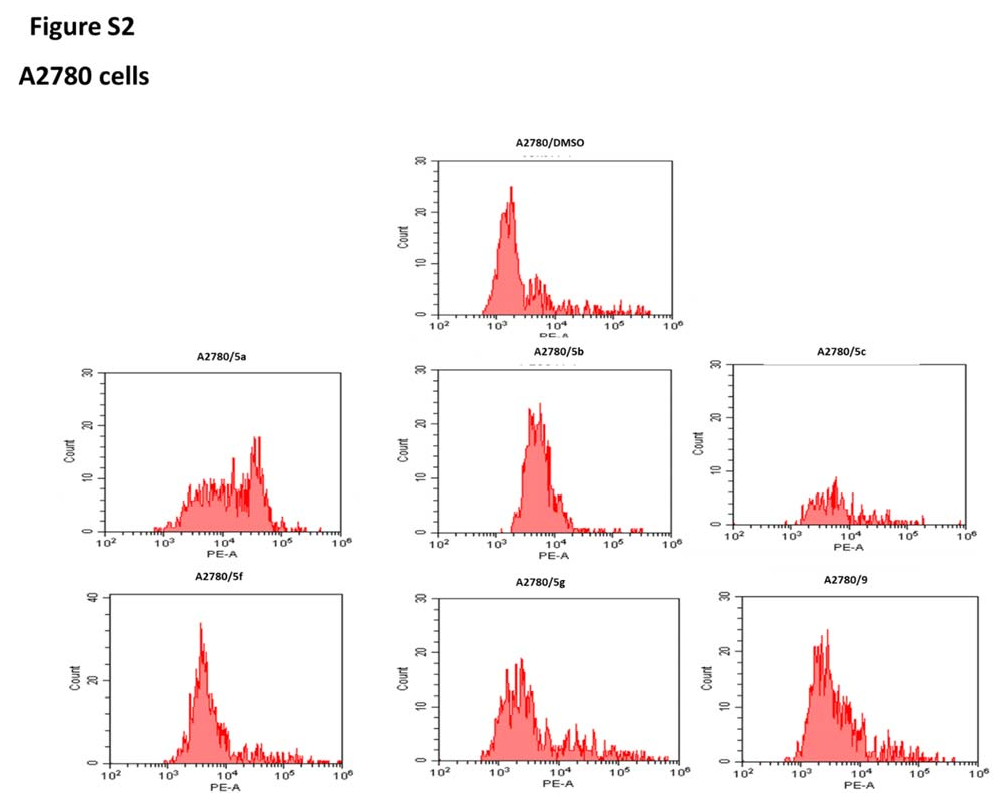


**Fig.(S2):** Cell cycle analysis for the tested compounds against A2780 cell line


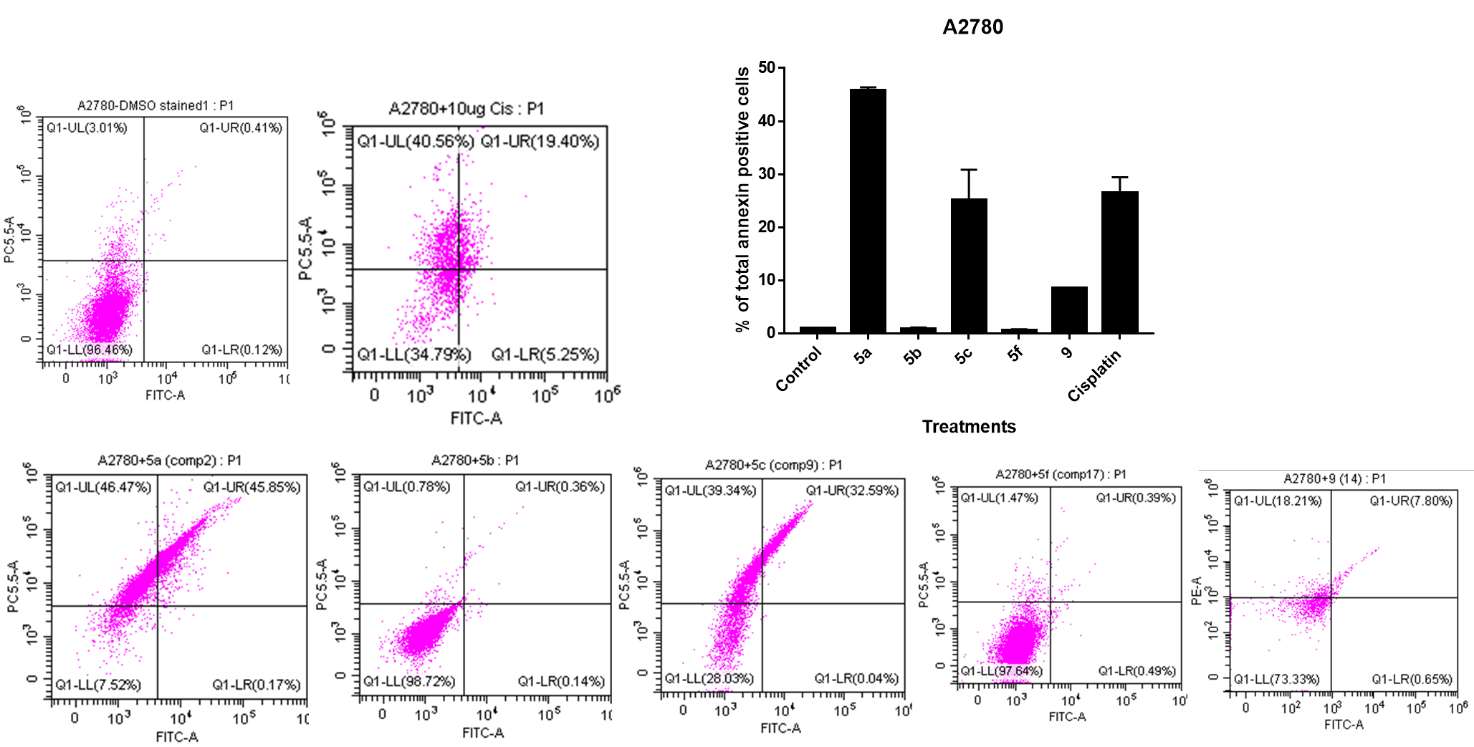


**Fig. (S2):** AnnexinV/PI staining for the tested compounds against A2780 cell line in triplicate

| **Table (): Summary of docking results of the tested compounds** | | | | | |
| --- | --- | --- | --- | --- | --- |
| **Docked compound** | **Binding energy (Kcal/mol)** | **Binding interactions** | | | |
|  |  | **H-bonding with key amino acids (Lys 67)** | | | **Other interactions** |
|  |  | **No** | **Interactive moiety** | **Length (^◦^A)** |  |
| **5a** | -11.46 | **2** | **-N-** as HBA  **-N-** as HBA | 1.43  1.26 | One arene-cation interaction with Lys 169 One HB as HBD with Asp 186 |
|  |  |  |  |  | Lipophilic interactions with lipophilic (greasy) amino acids |
| 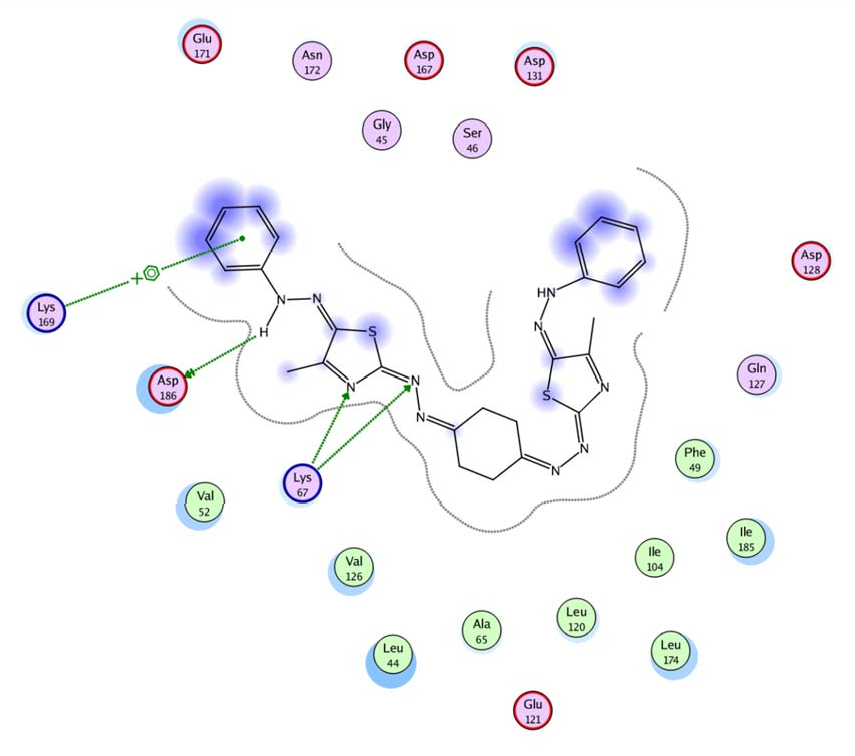 | | | | | |
| **5b** | -12.66 | **2** | **-N-** as HBA  **-N-** as HBA | 2.01 | One arene-cation interaction with Lys 169 One HB as HBD with Asp 186 |

|  |  |  |  | 1.76 | Lipophilic interactions with lipophilic (greasy) amino acids |
| --- | --- | --- | --- | --- | --- |
| 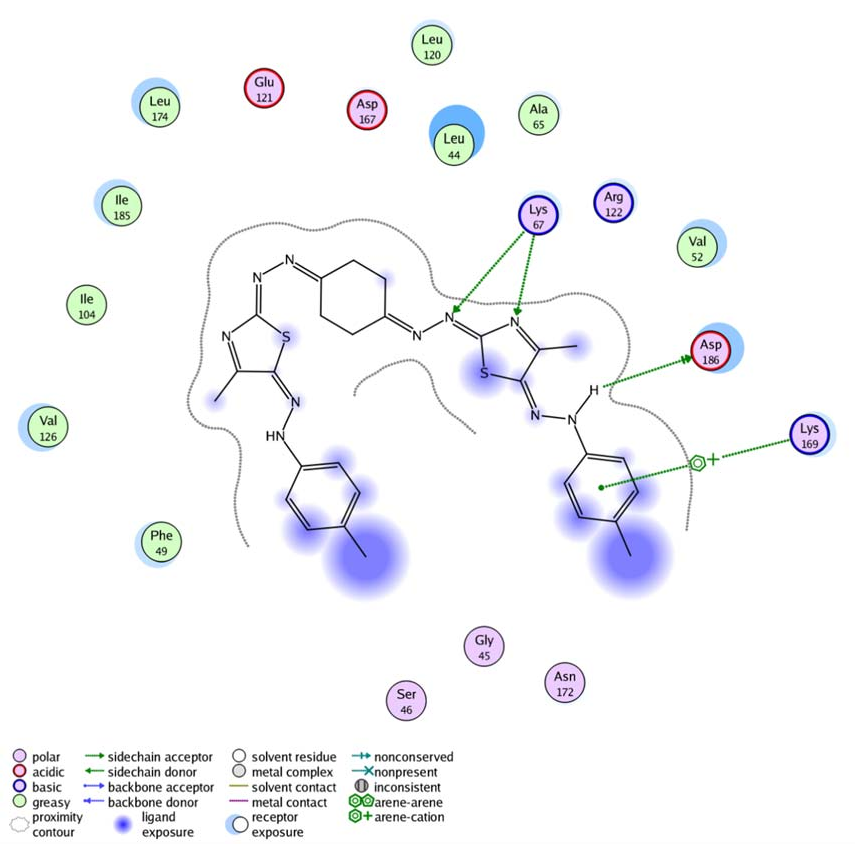 | | | | | |
| **5d** | -13.08 | **2** | **-N-** as HBA  **-N-** as HBA | 1.86  1.65 | One HB as HBD with Asp 186  Lipophilic interactions with lipophilic (greasy) amino acids |

| 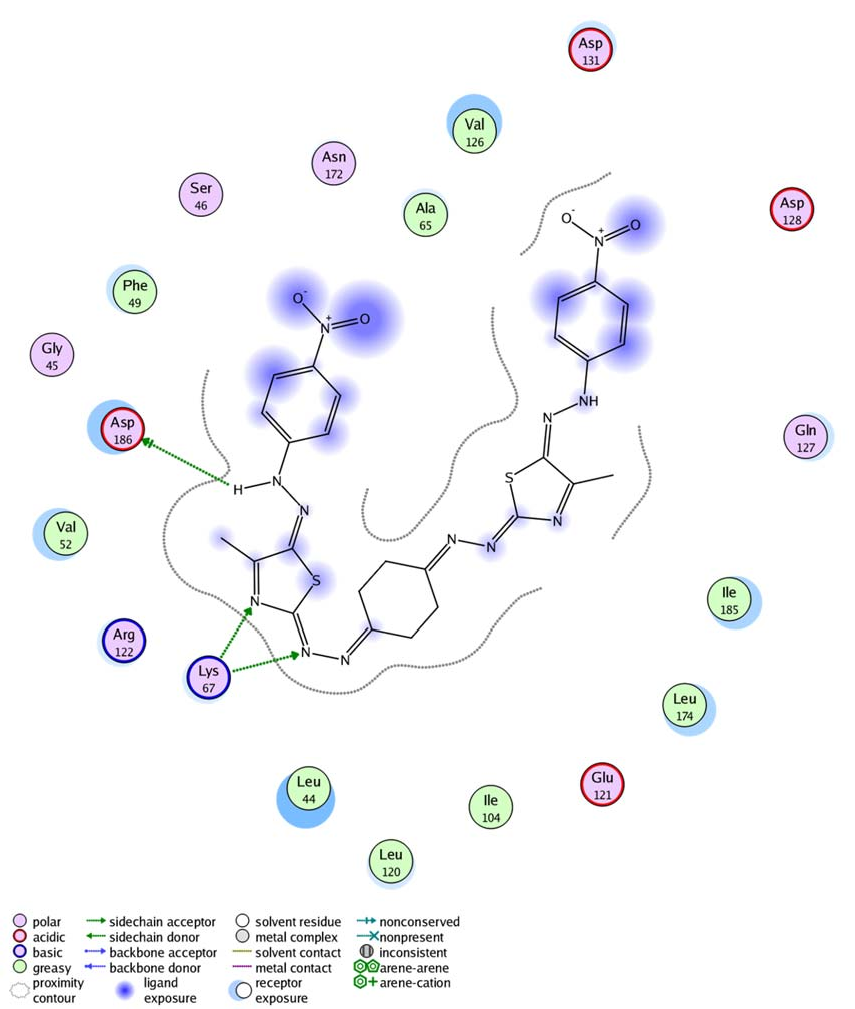 | | | | | |
| --- | --- | --- | --- | --- | --- |
| **13a** | -14.24 | **1** | **-N-** as HBA | 2.12 | One HB as HBD with Asp 186  Lipophilic interactions with lipophilic (greasy) amino acids |

| 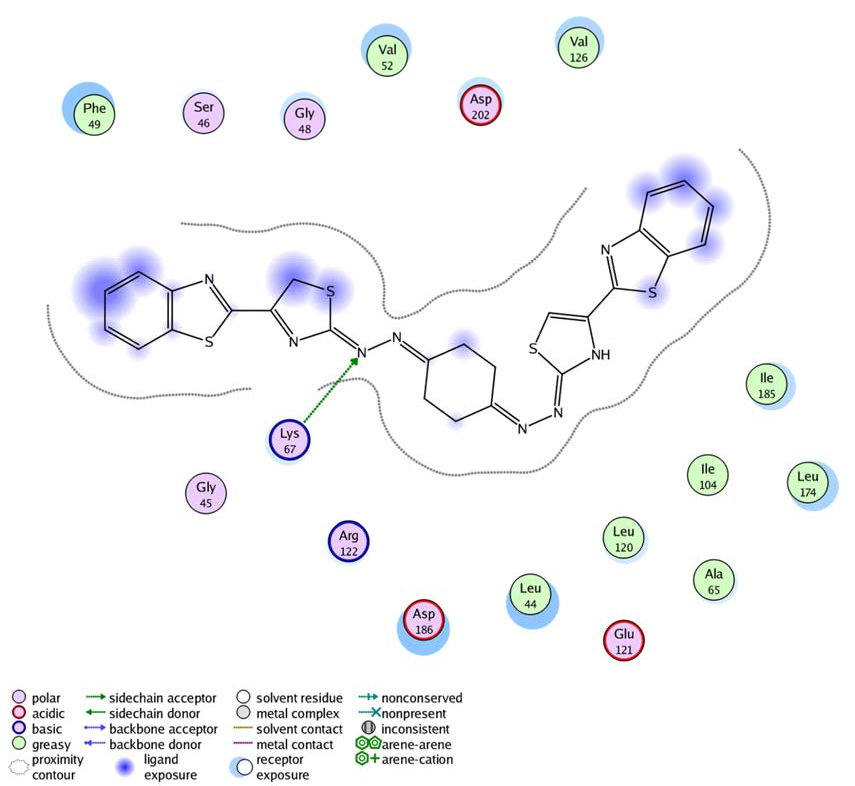 | | | | | |
| --- | --- | --- | --- | --- | --- |
| **13c** | -25.59 | 1 | **-C=O** HBA | 1.87 | One HB as HBD with Asp 186  Lipophilic interactions with lipophilic (greasy) amino acids |

| 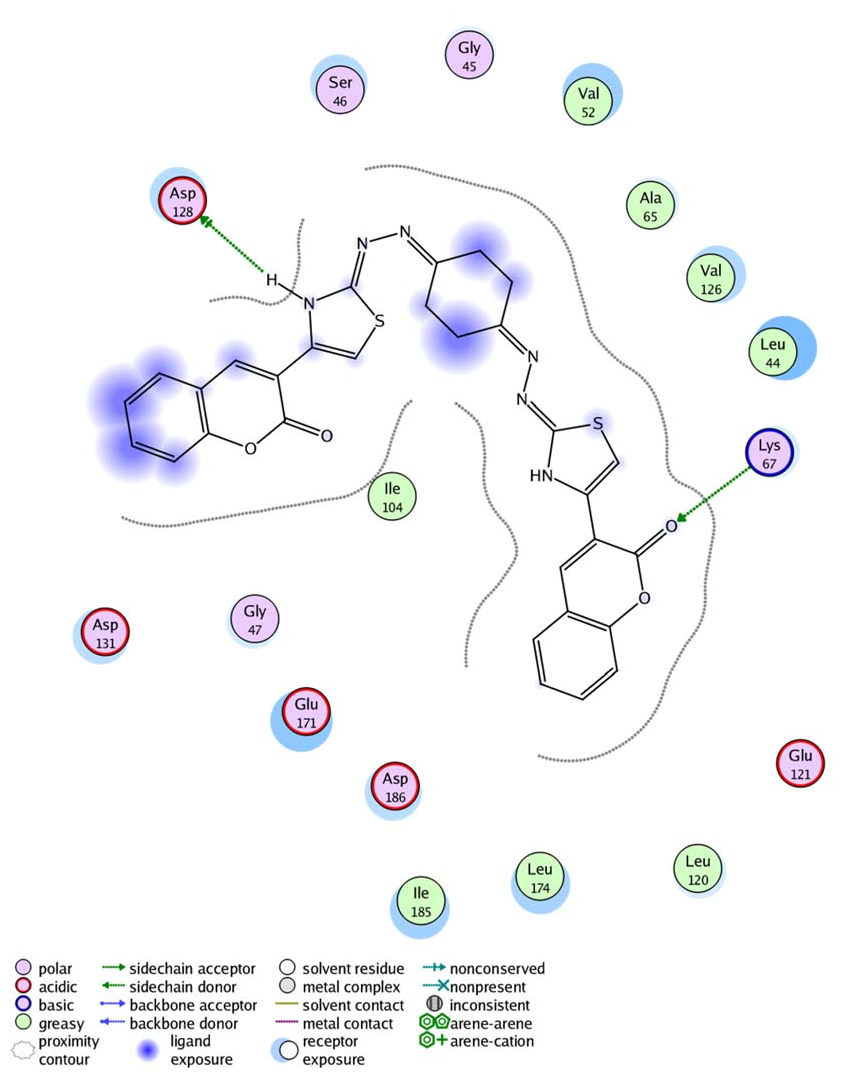 | | | | | |
| --- | --- | --- | --- | --- | --- |
| **9** | -14.42 | 1 | -**N-** HBA | 2.32 | Lipophilic interactions with lipophilic (greasy) amino acids |


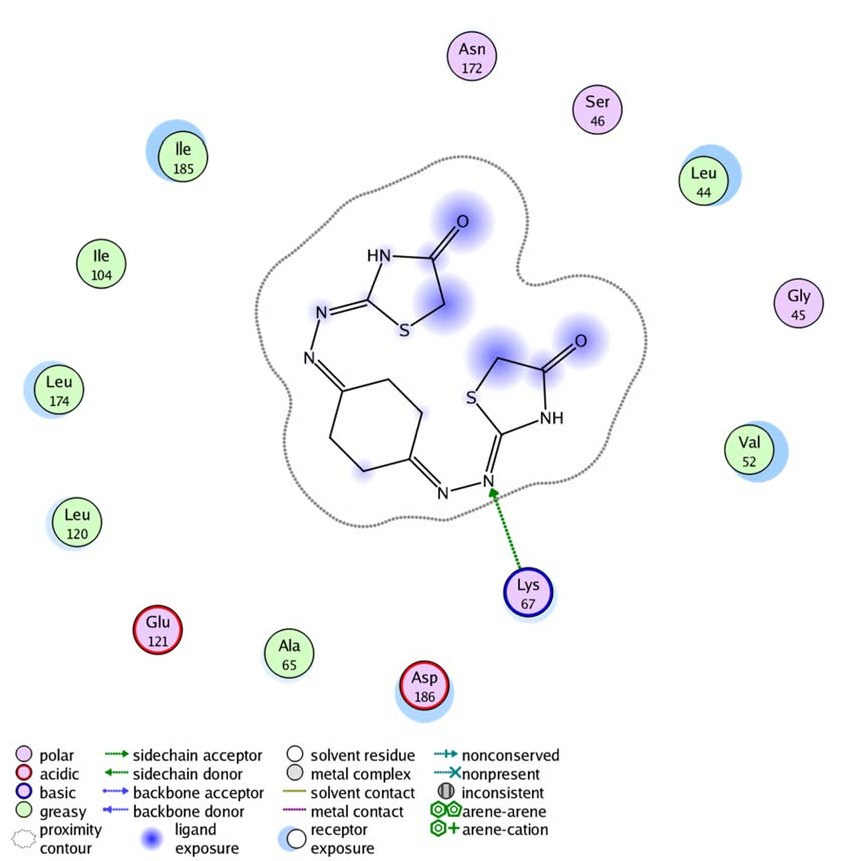


The rest of compounds were not able to be docked inside the tested protein

**Characterization charts**


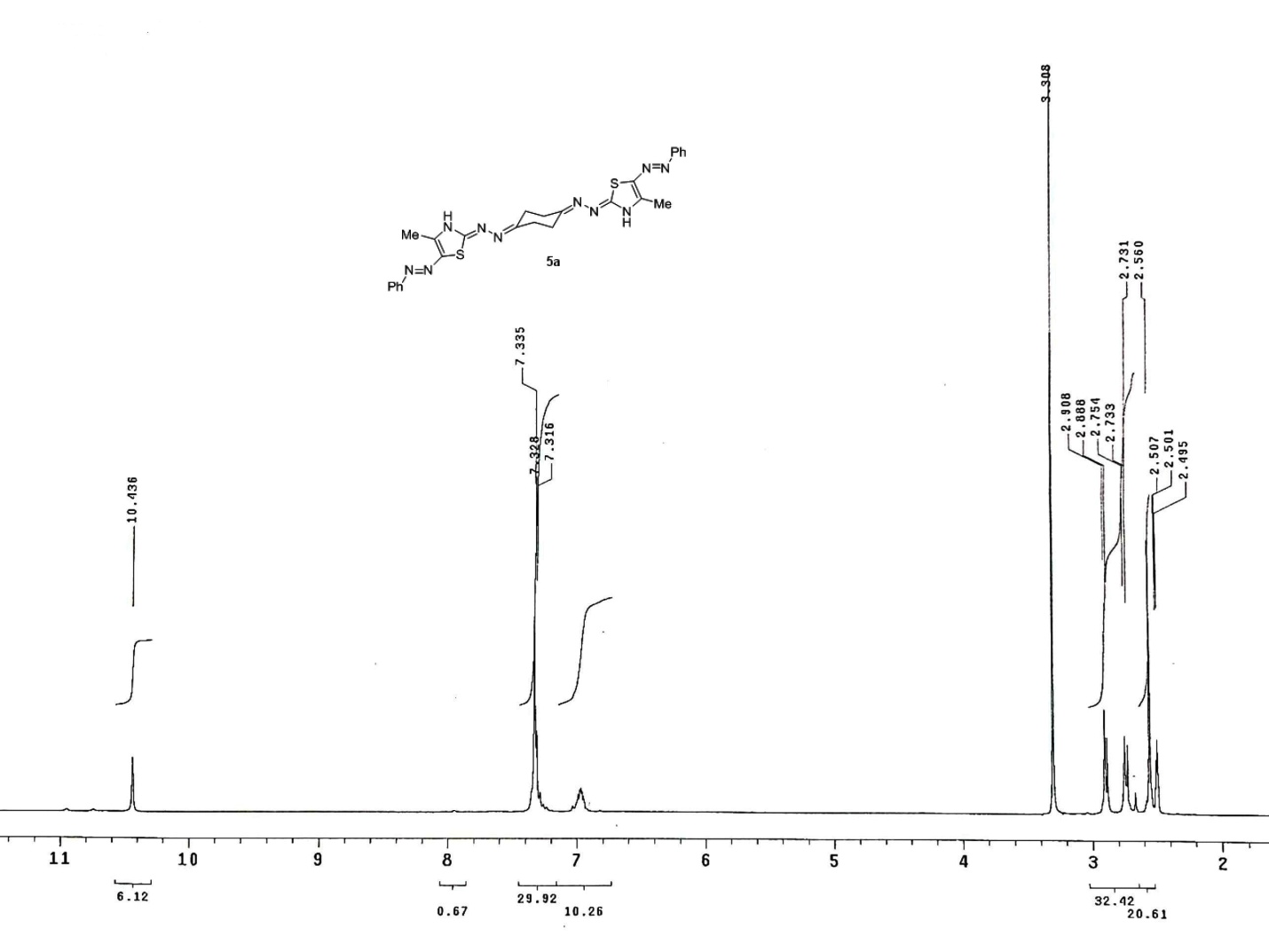


1H NMR spectrum of compound 5a


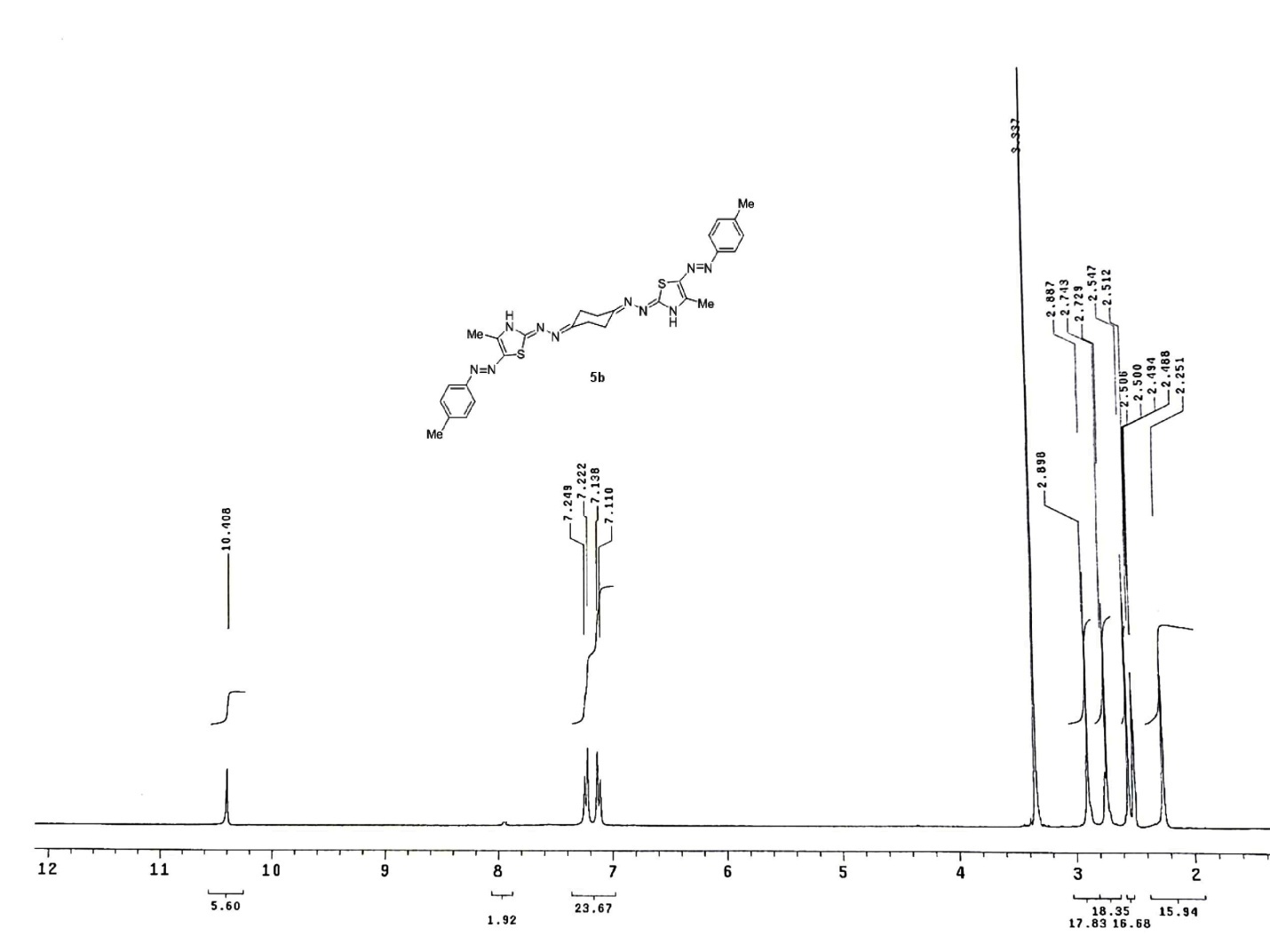


1H NMR spectrum of compound 5b


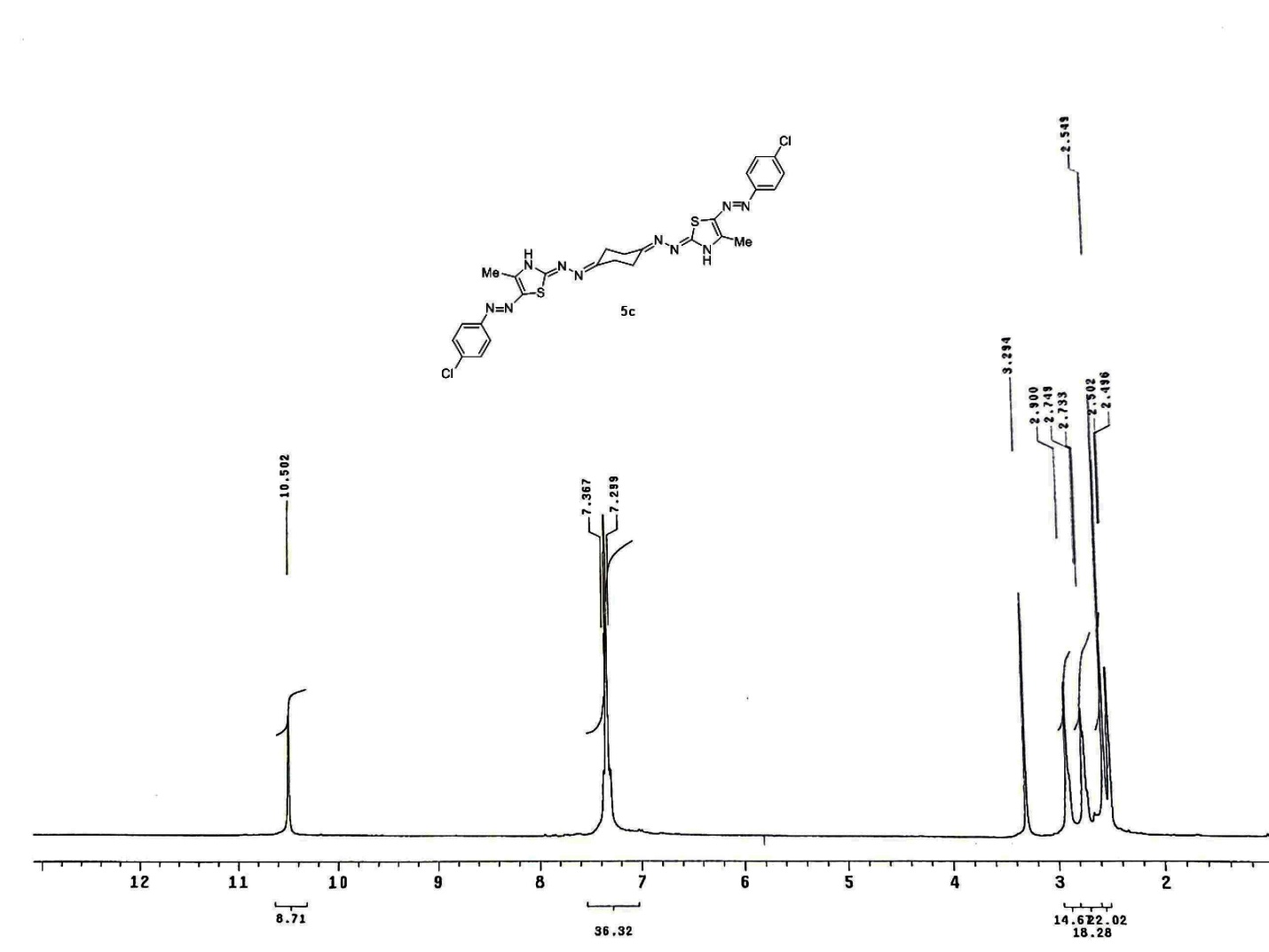


1H NMR spectrum of compound 5c


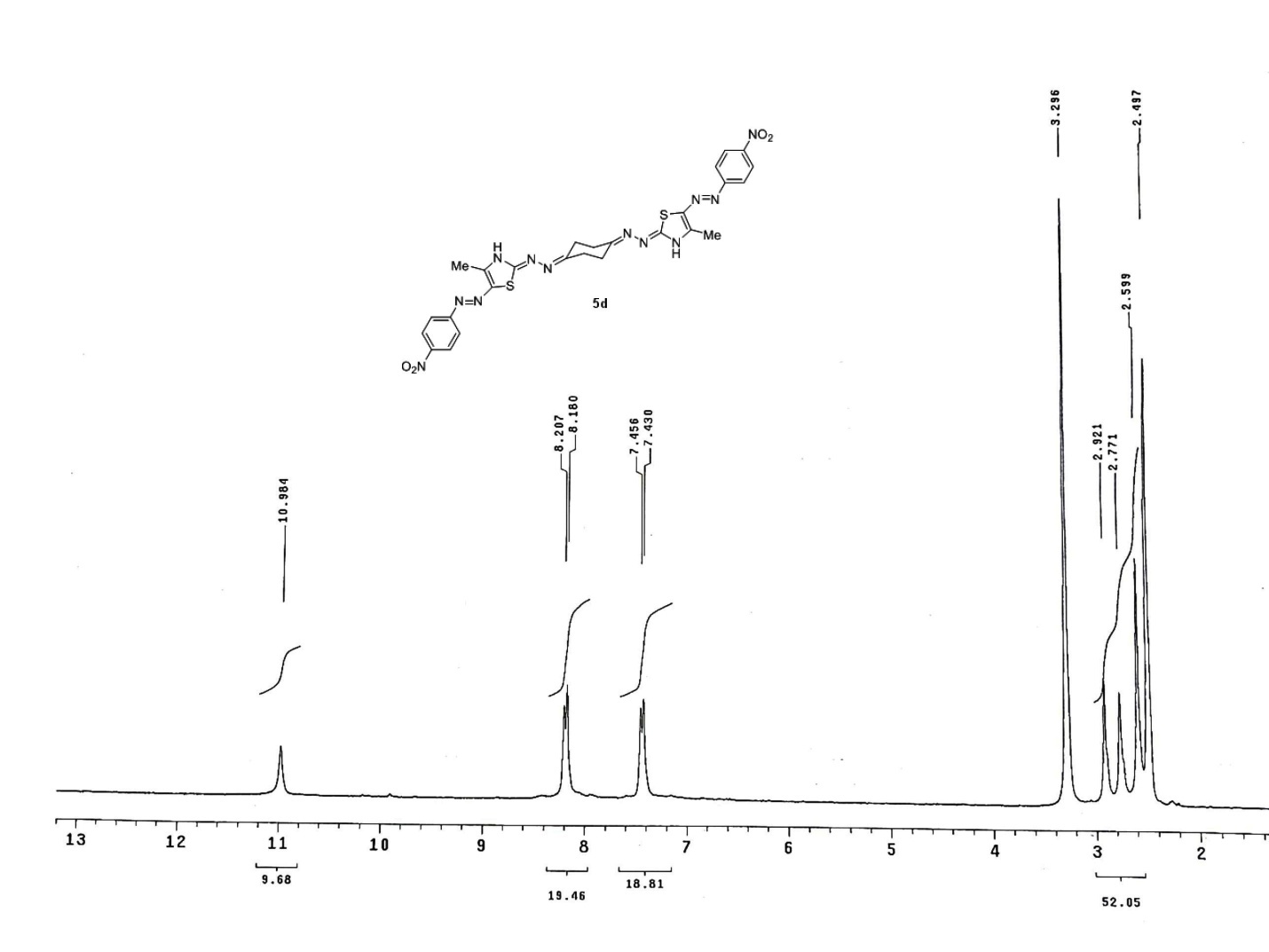


1H NMR spectrum of compound 5d


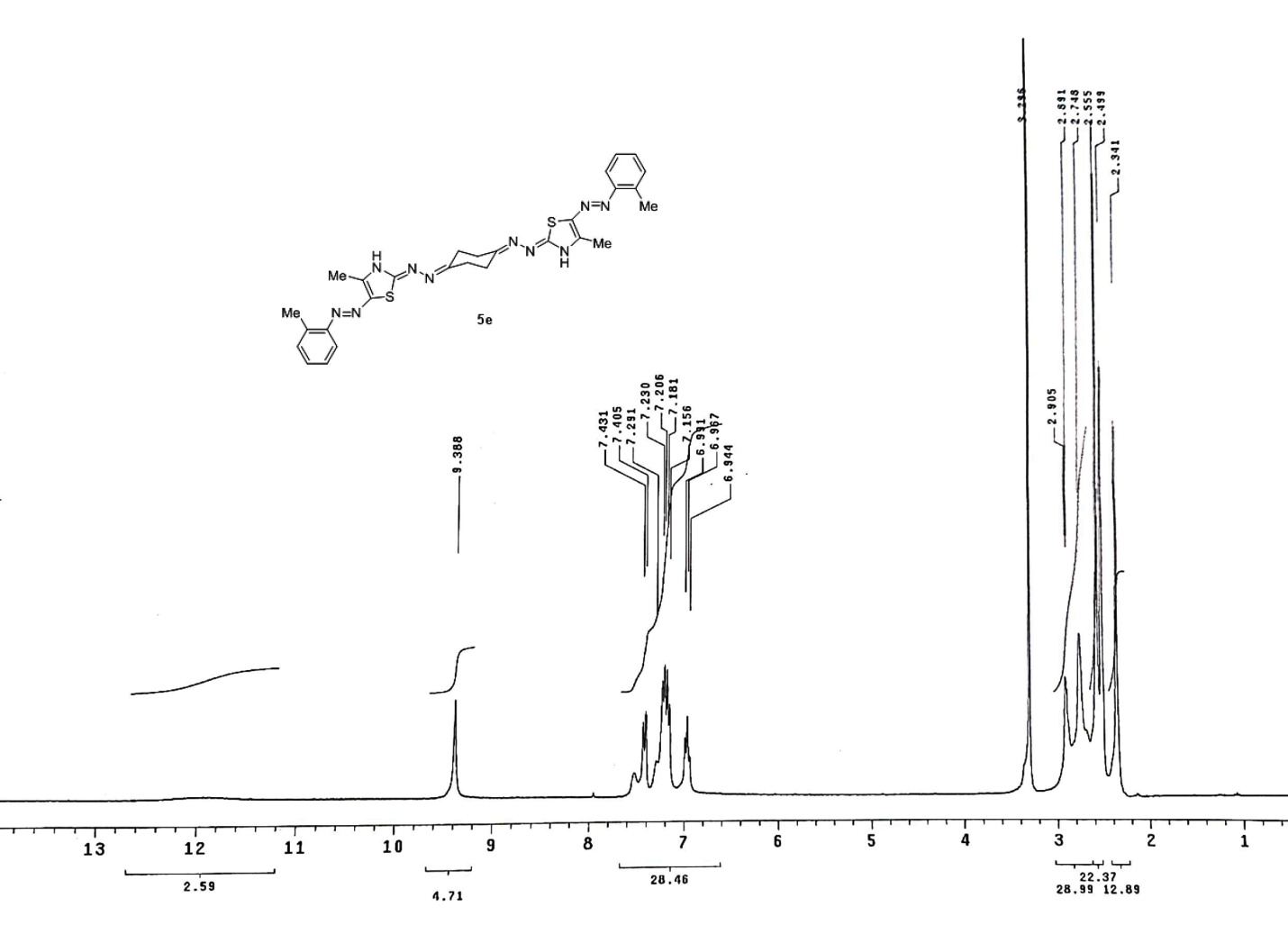


1H NMR spectrum of compound 5e


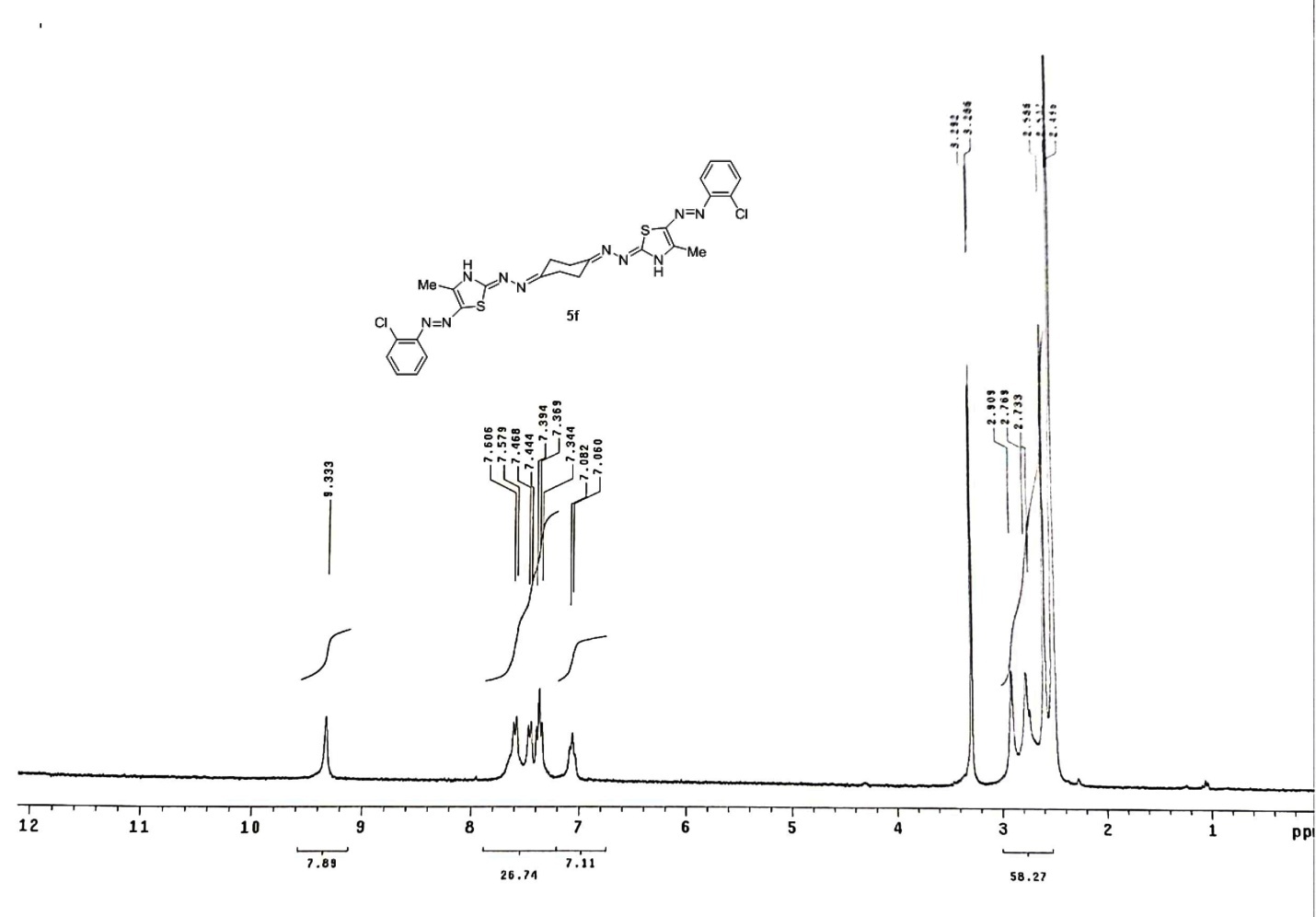


1H NMR spectrum of compound 5f


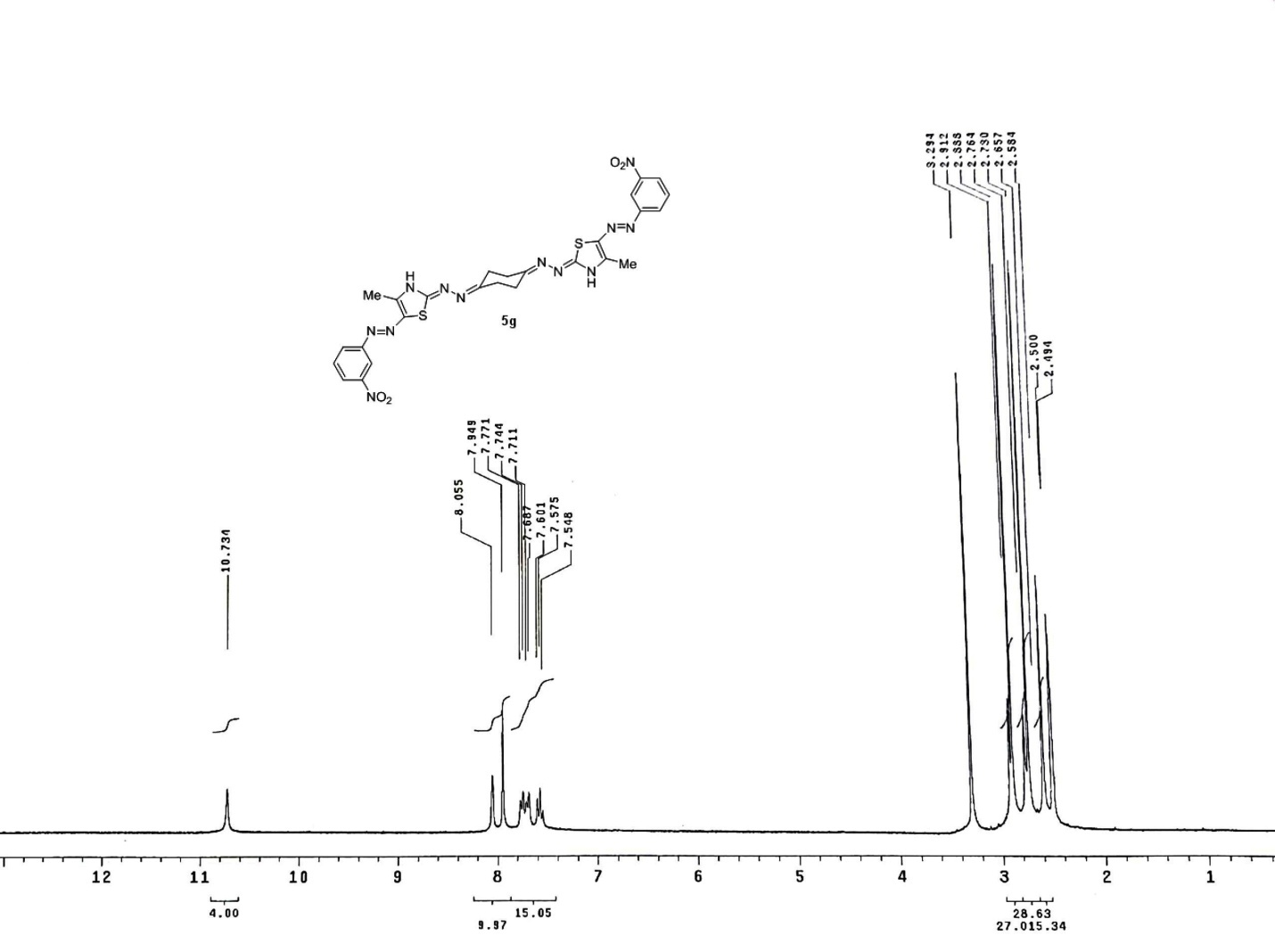


1H NMR spectrum of compound 5g


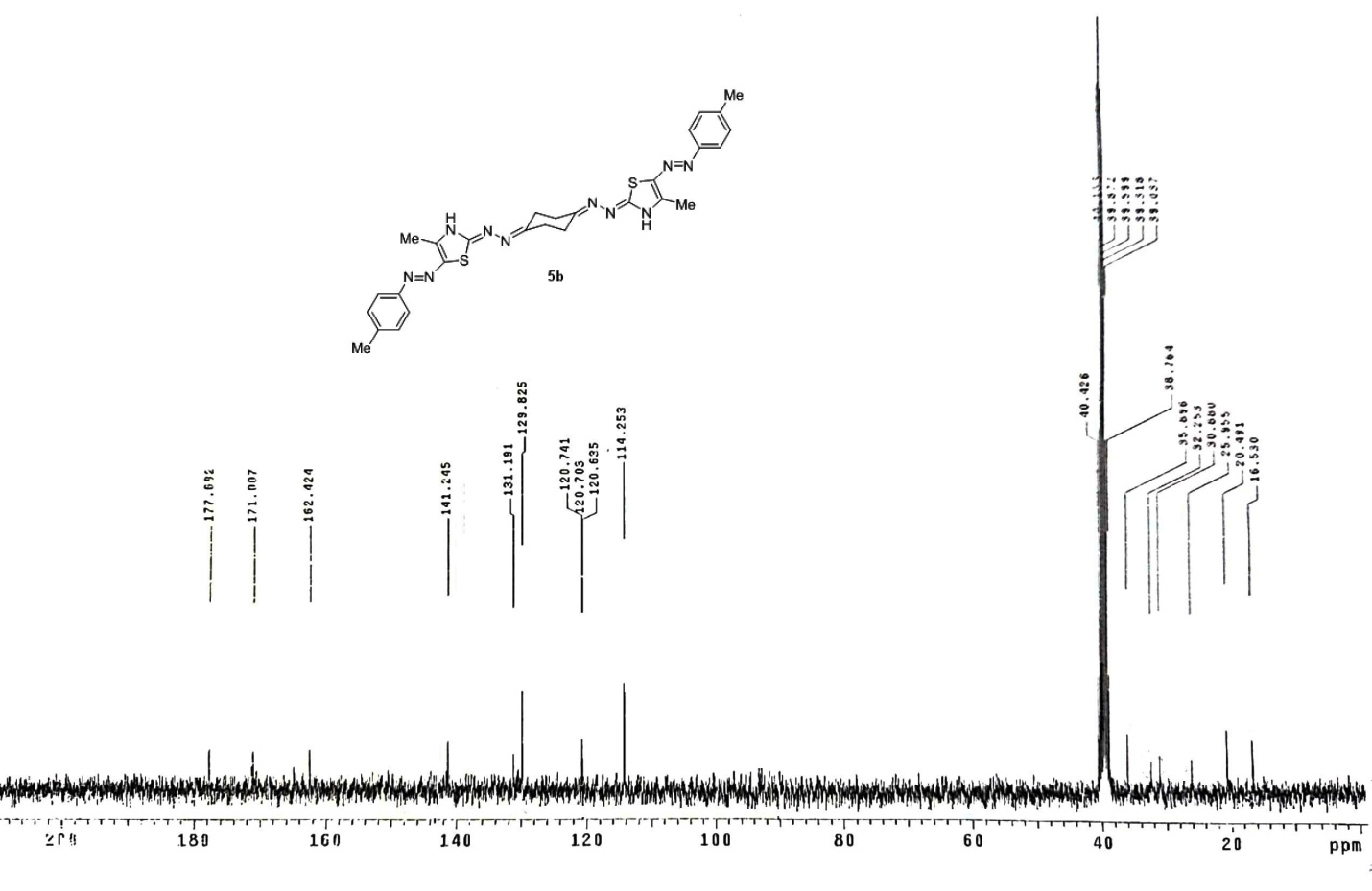


13C NMR spectrum of compound 5b


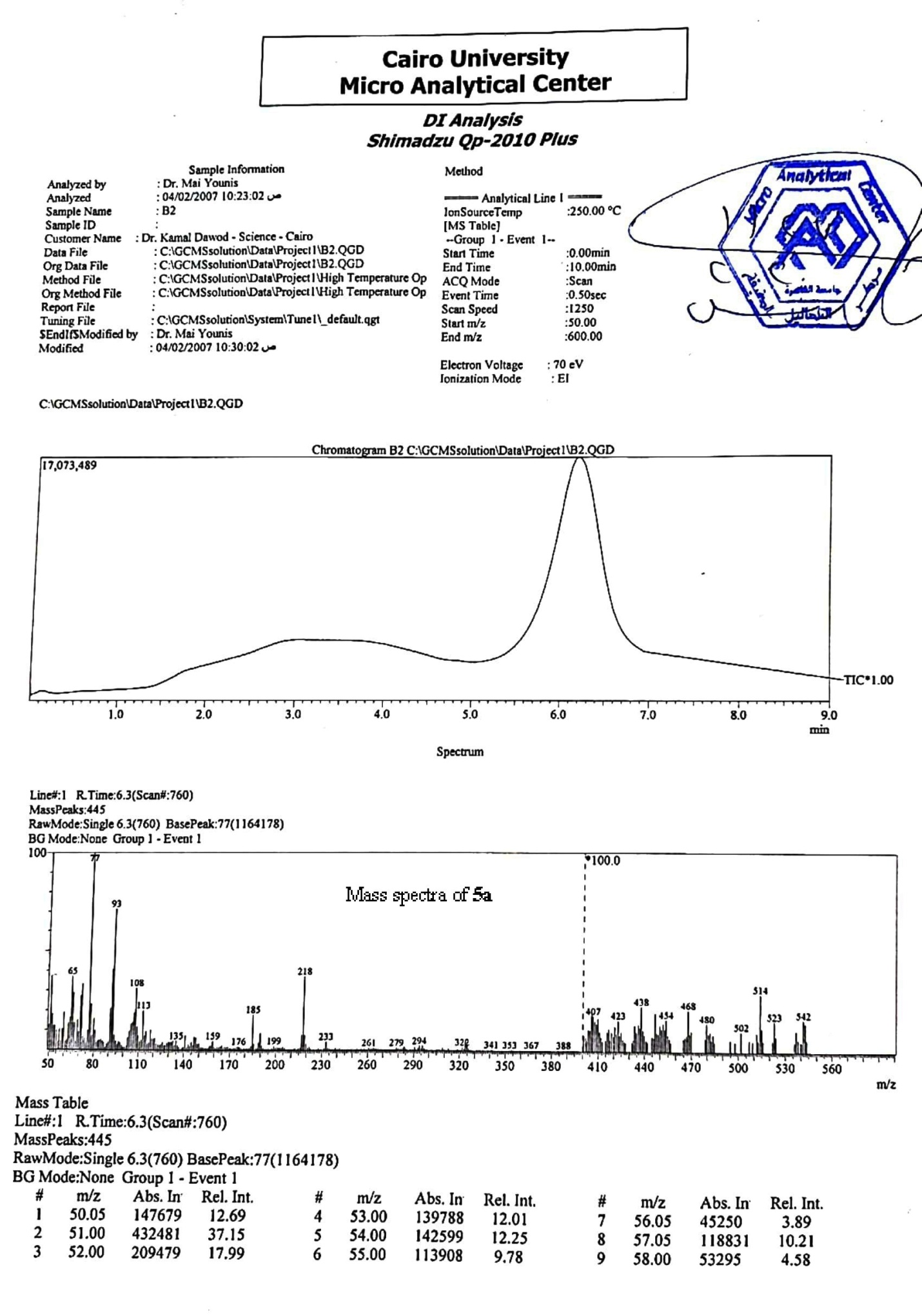


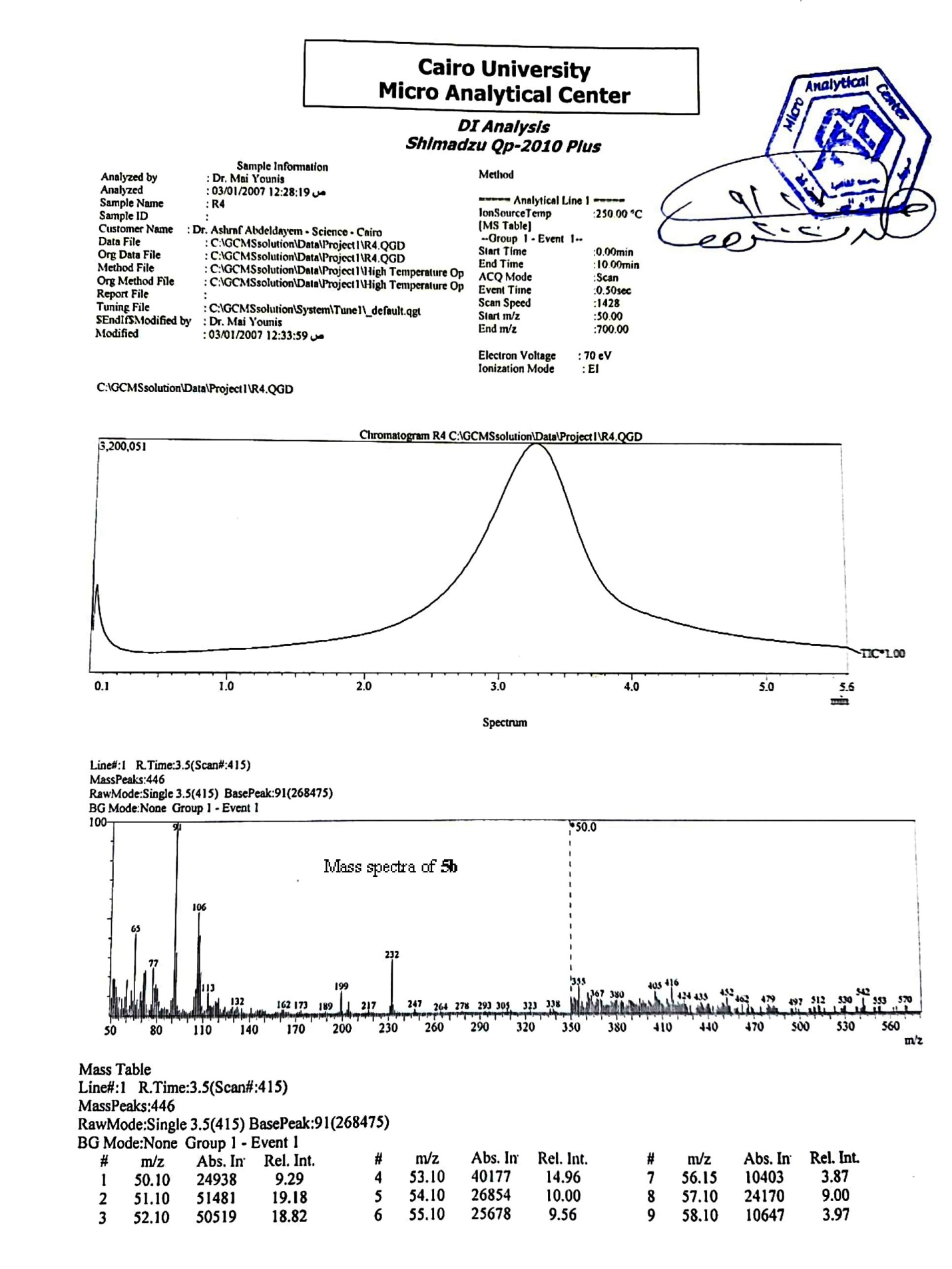


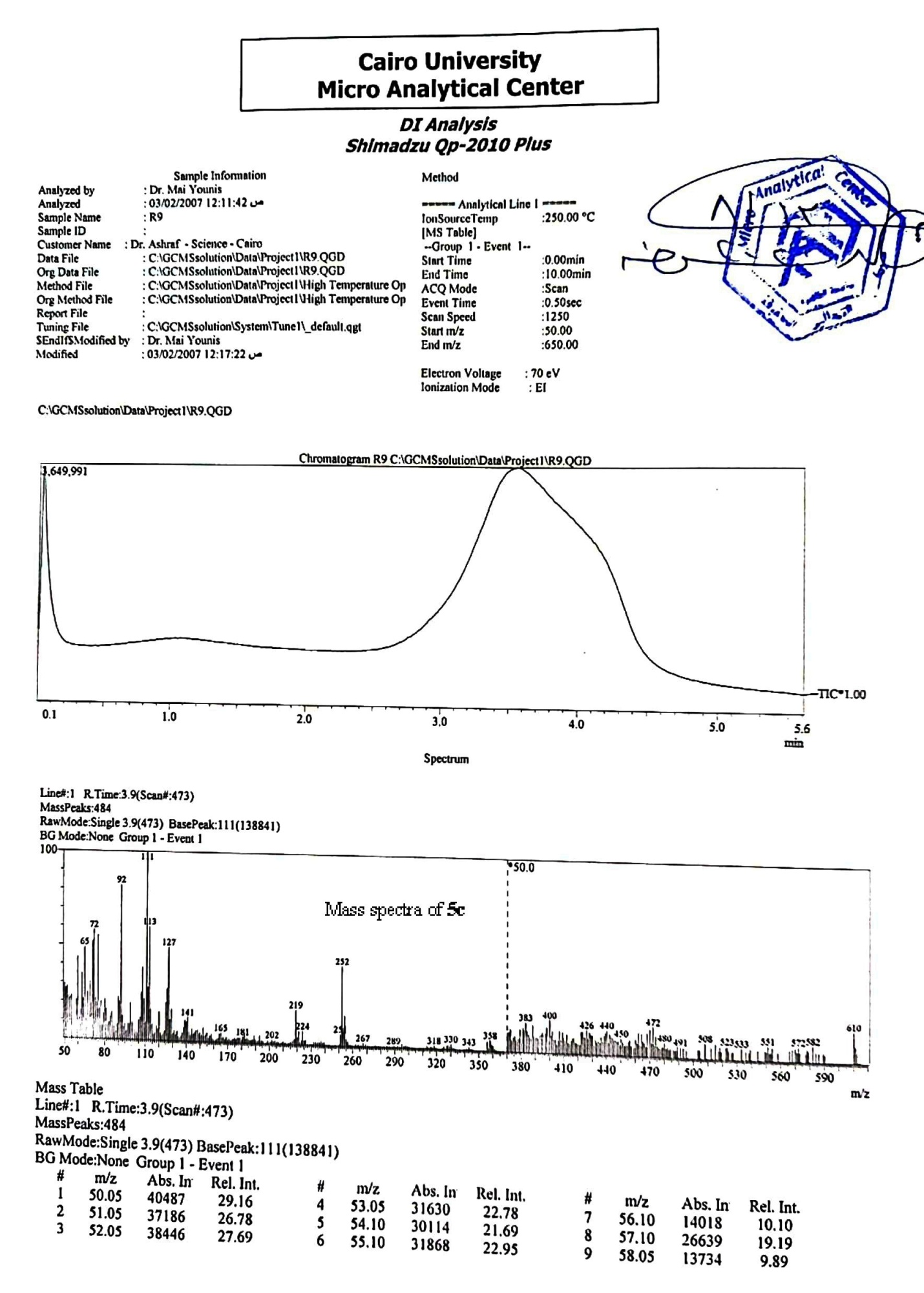


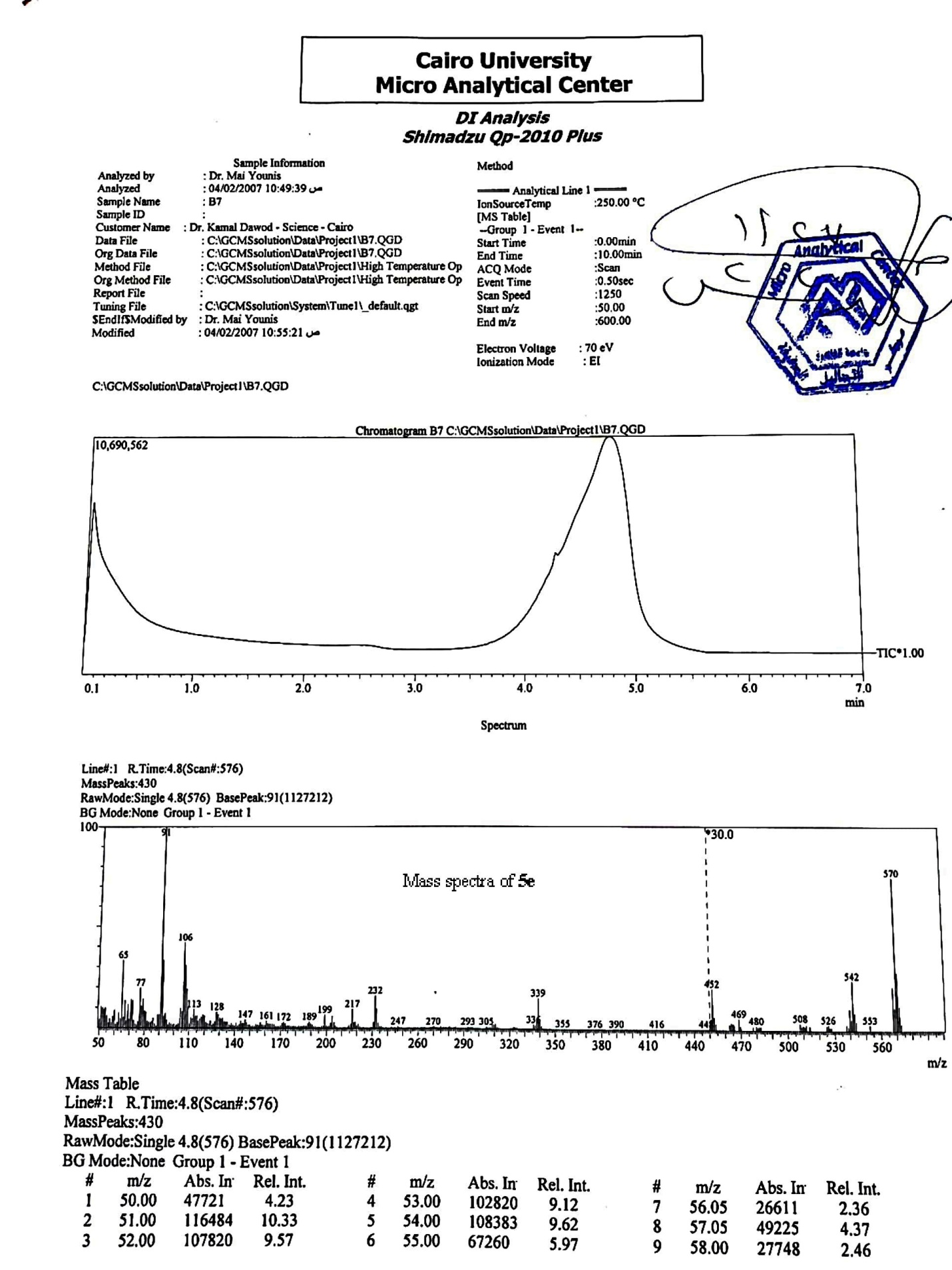


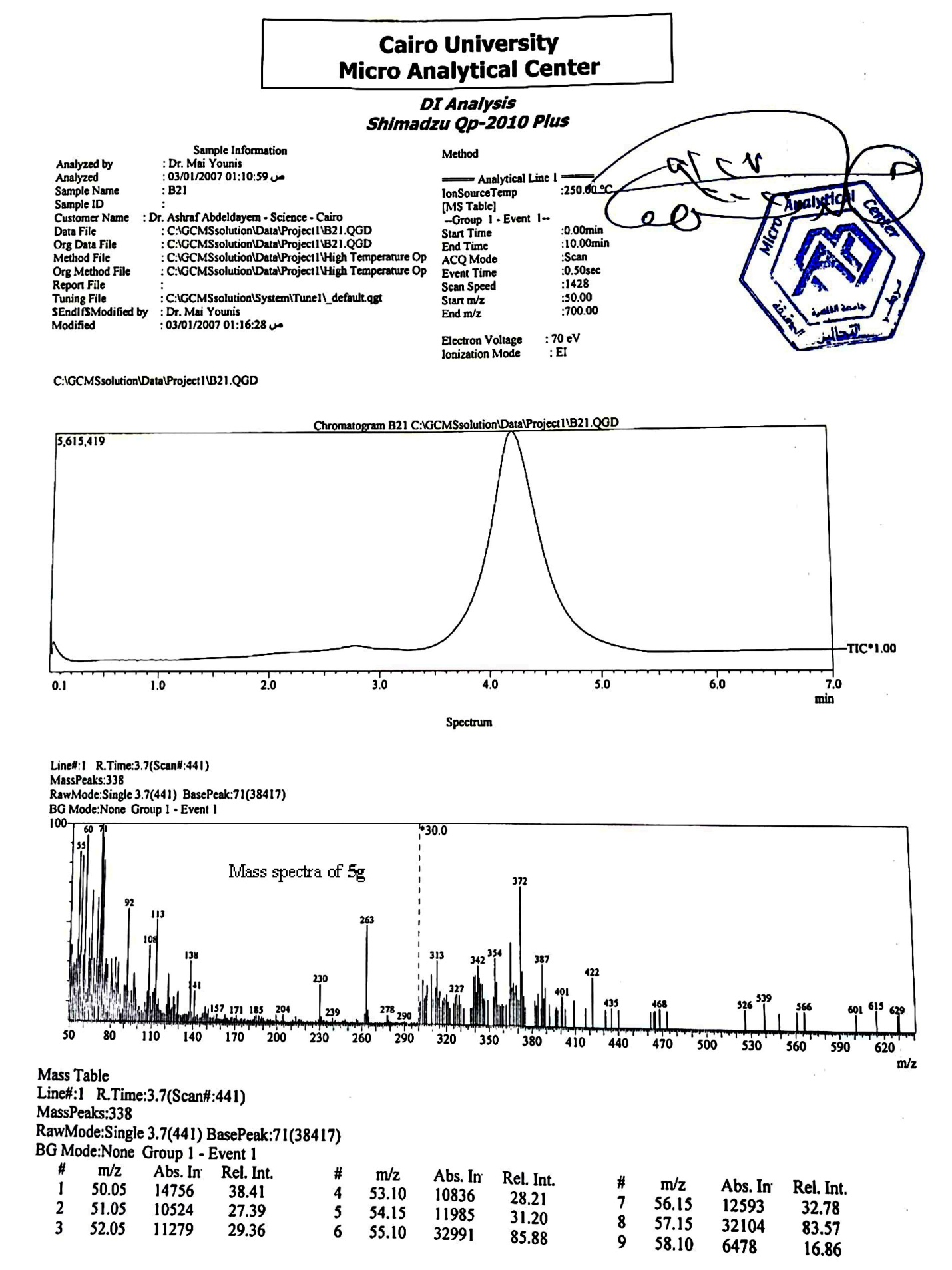


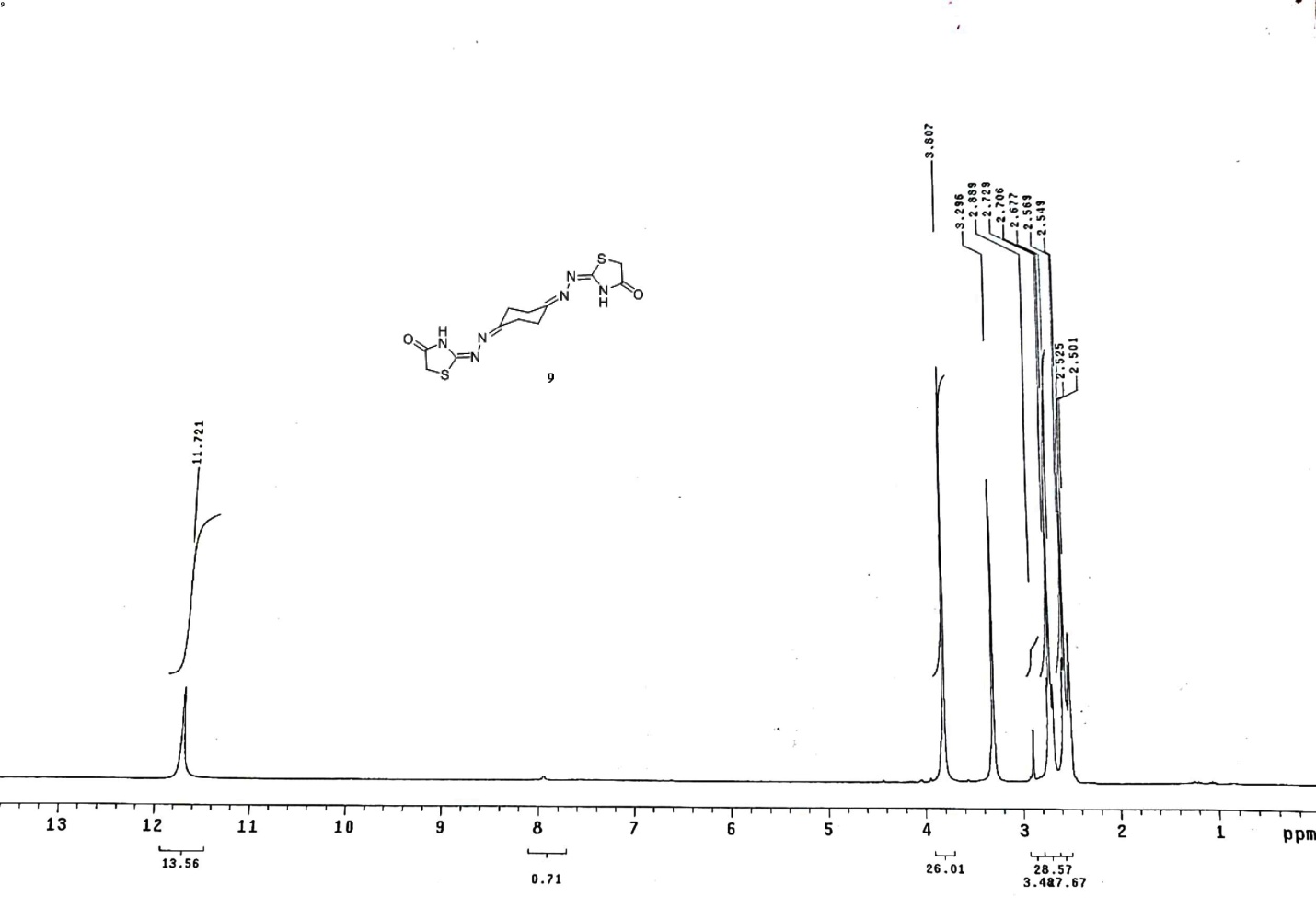


1H NMR spectrum of compound 9


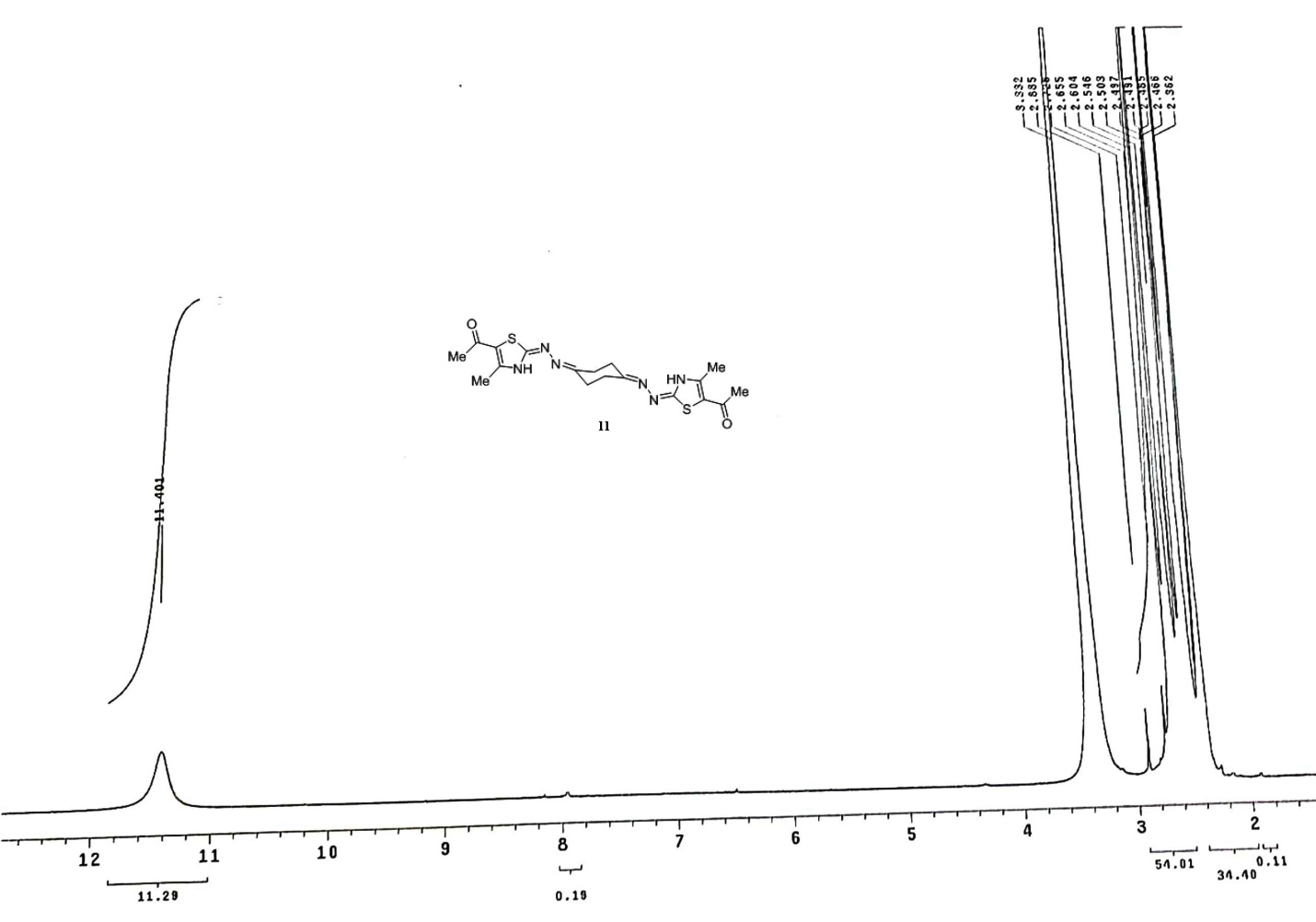


1H NMR spectrum of compound 11


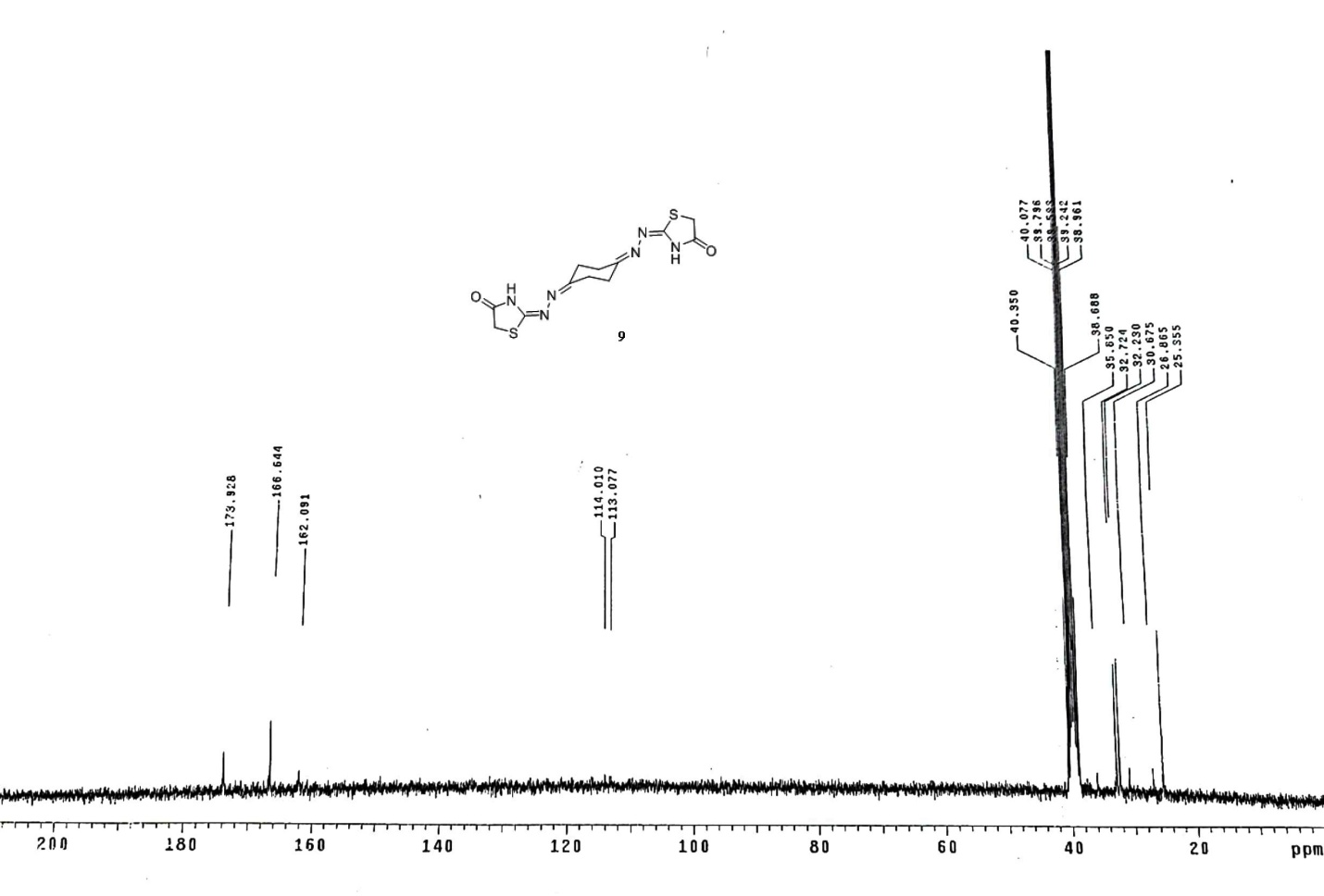


13C NMR spectrum of compound 9


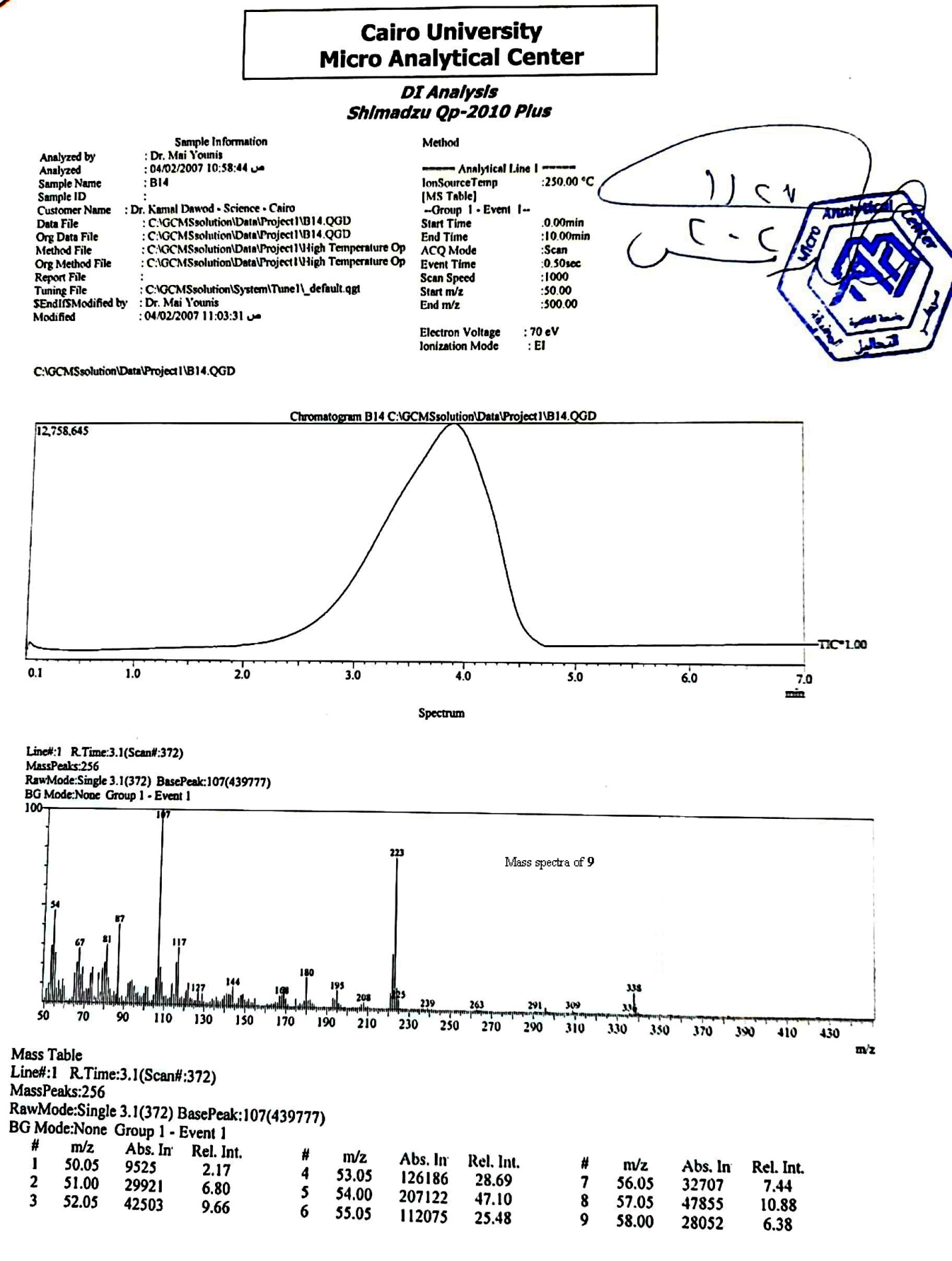


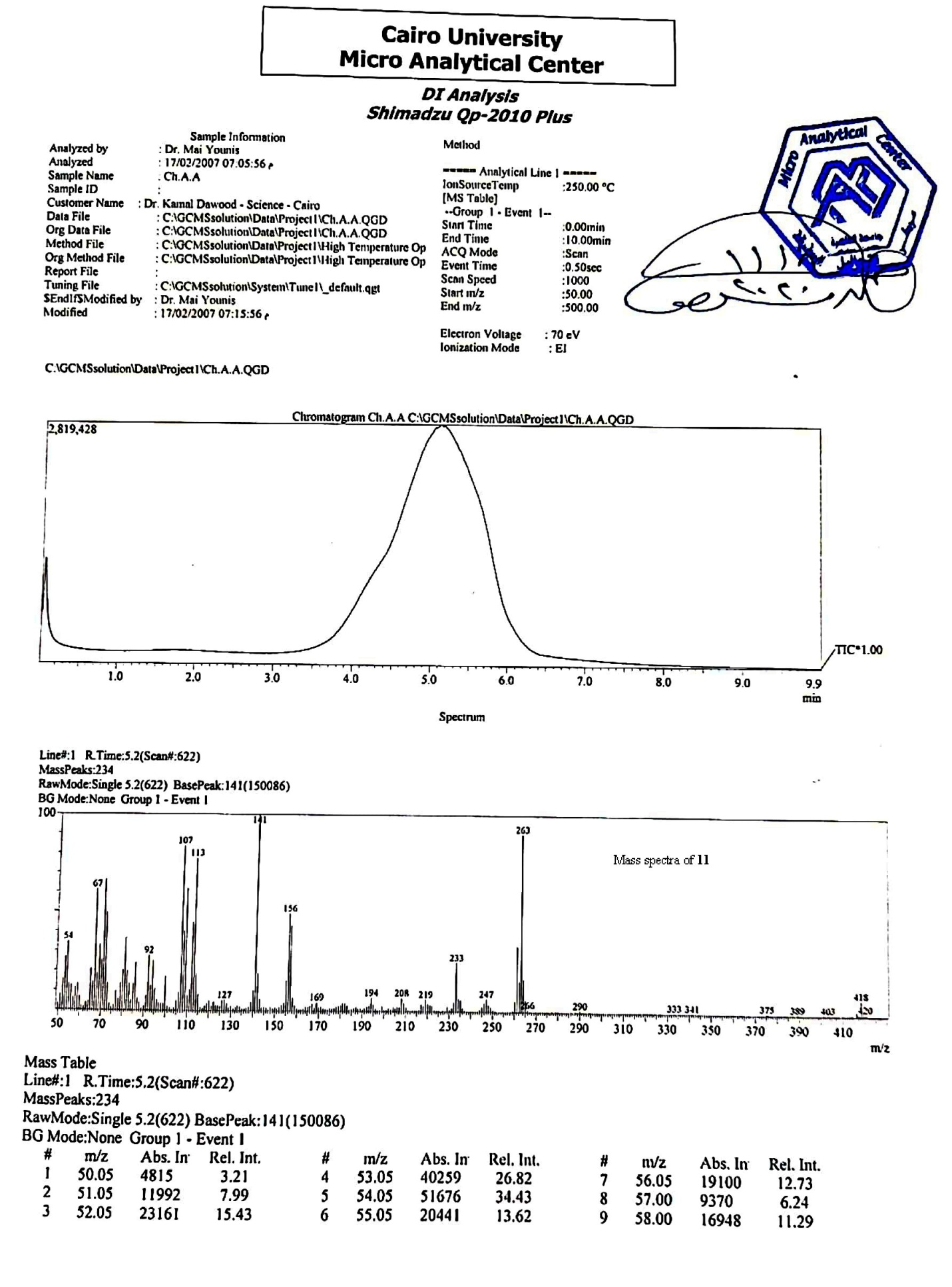


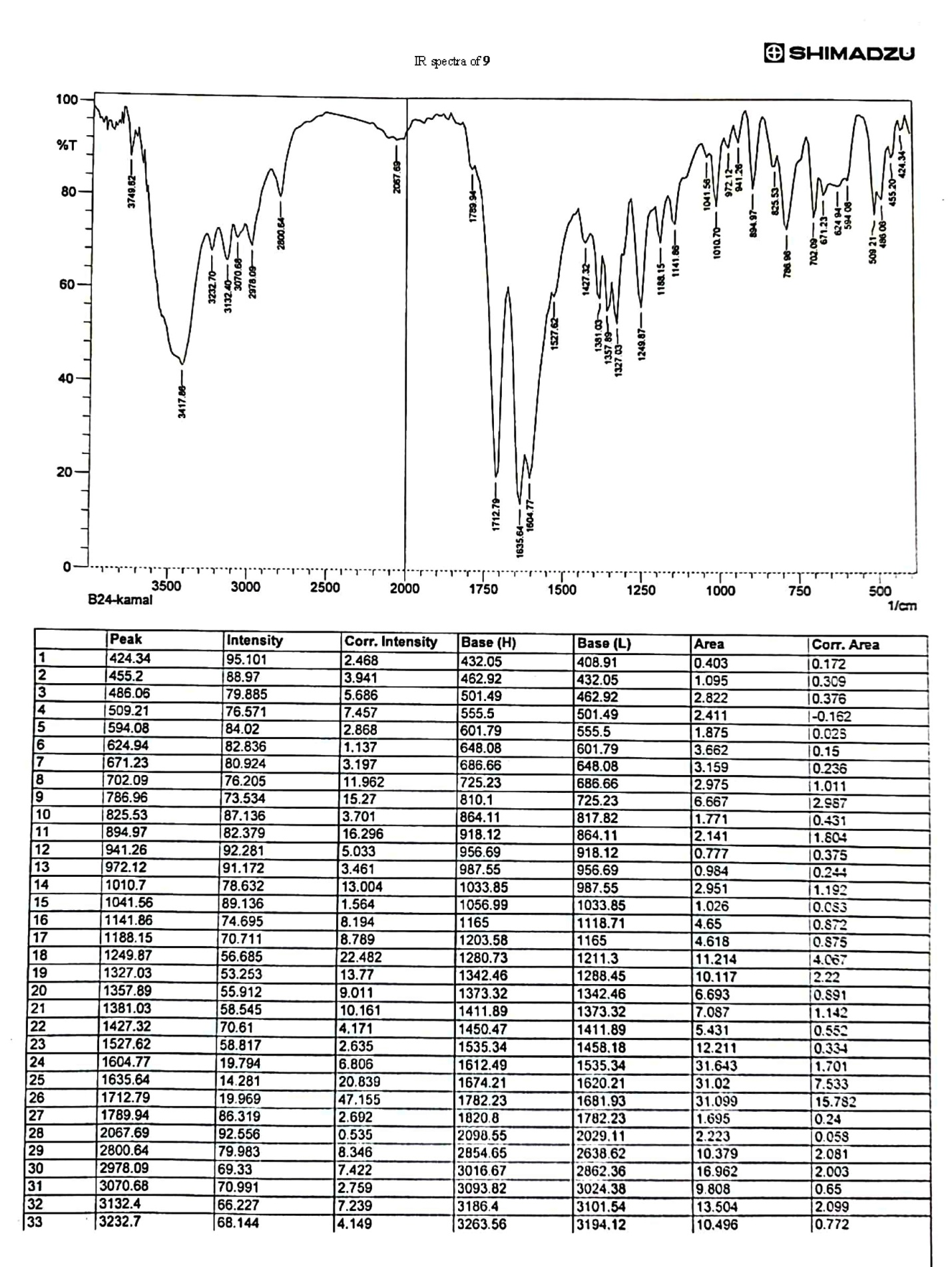


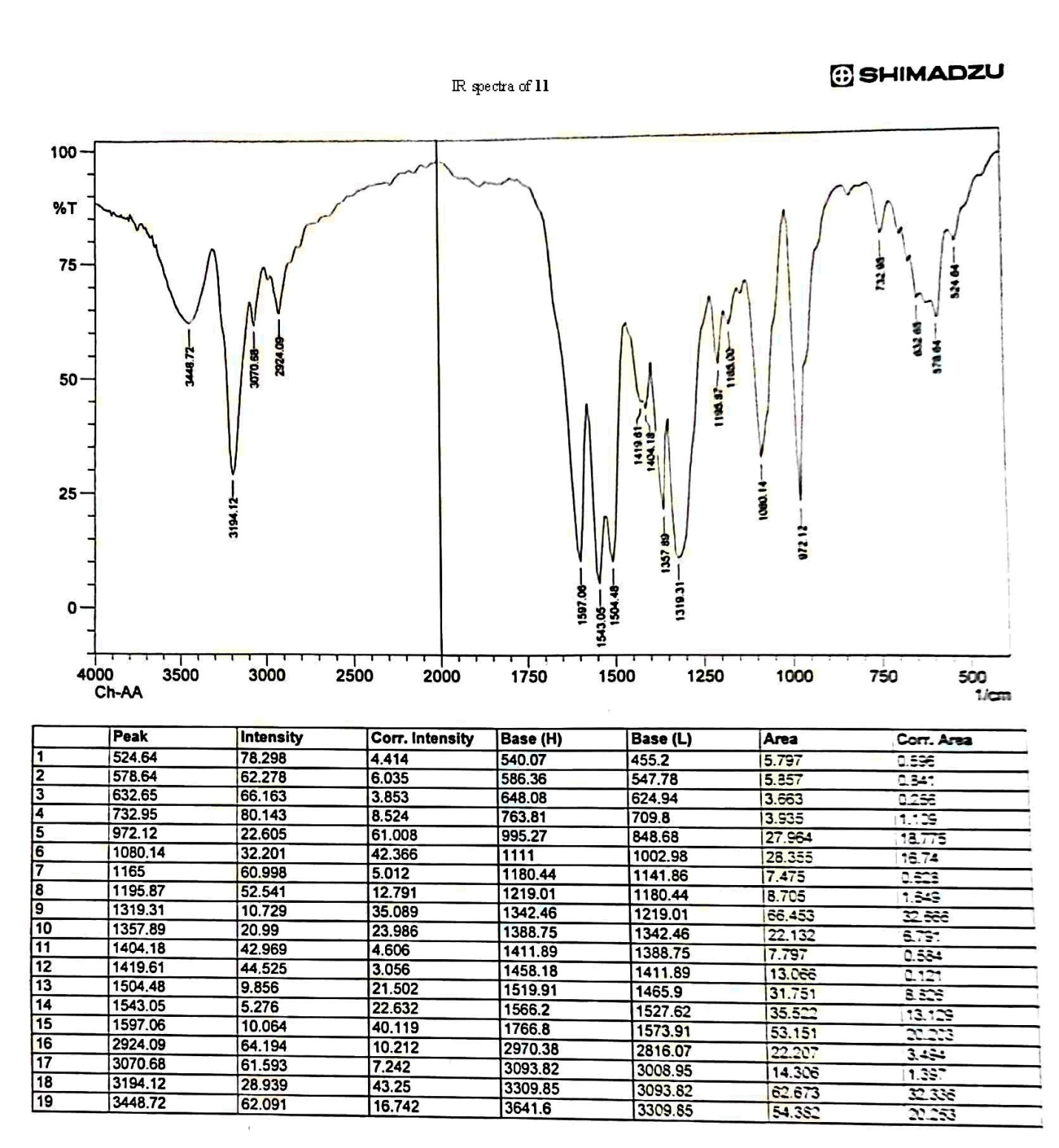


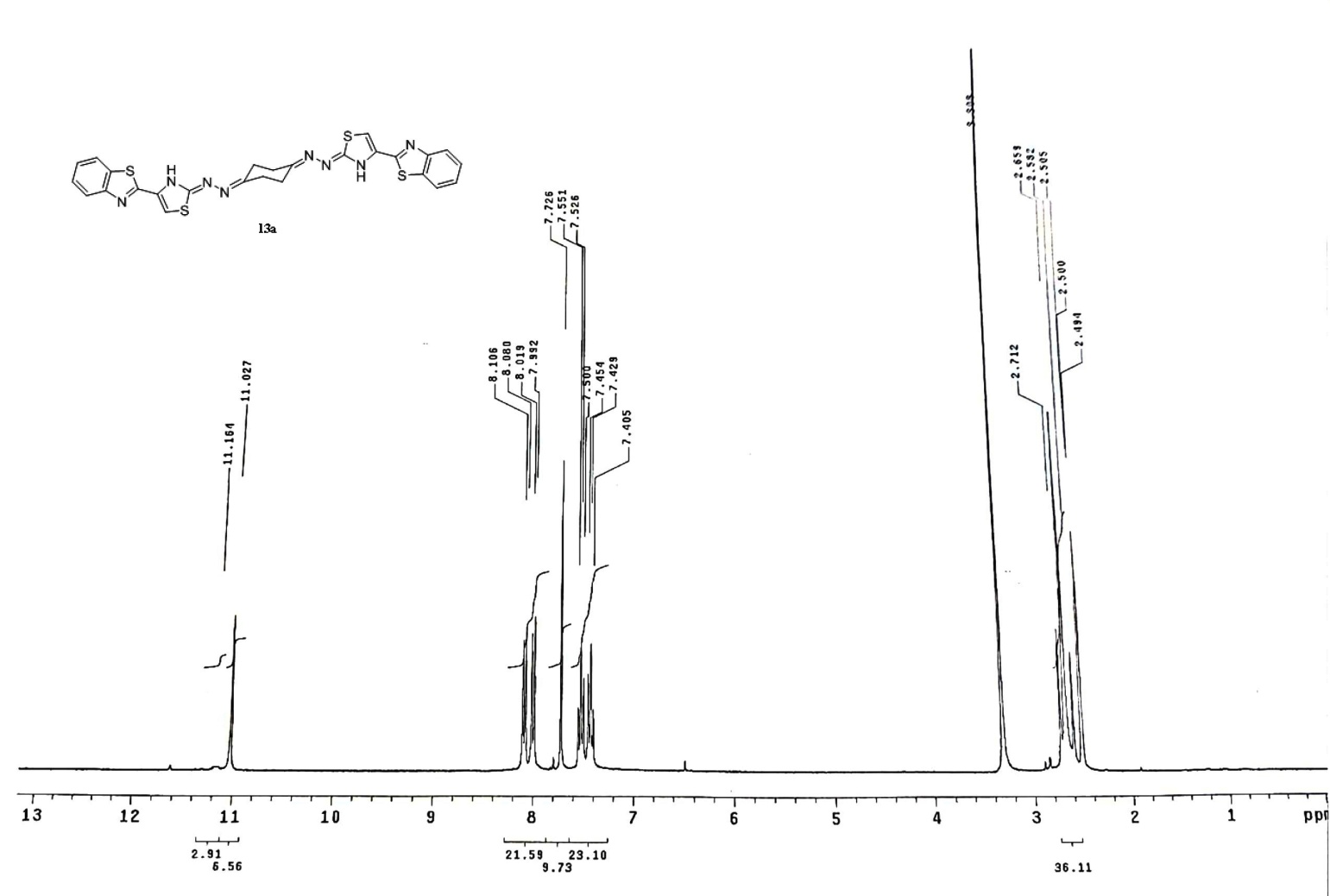


1H NMR spectrum of compound 13a


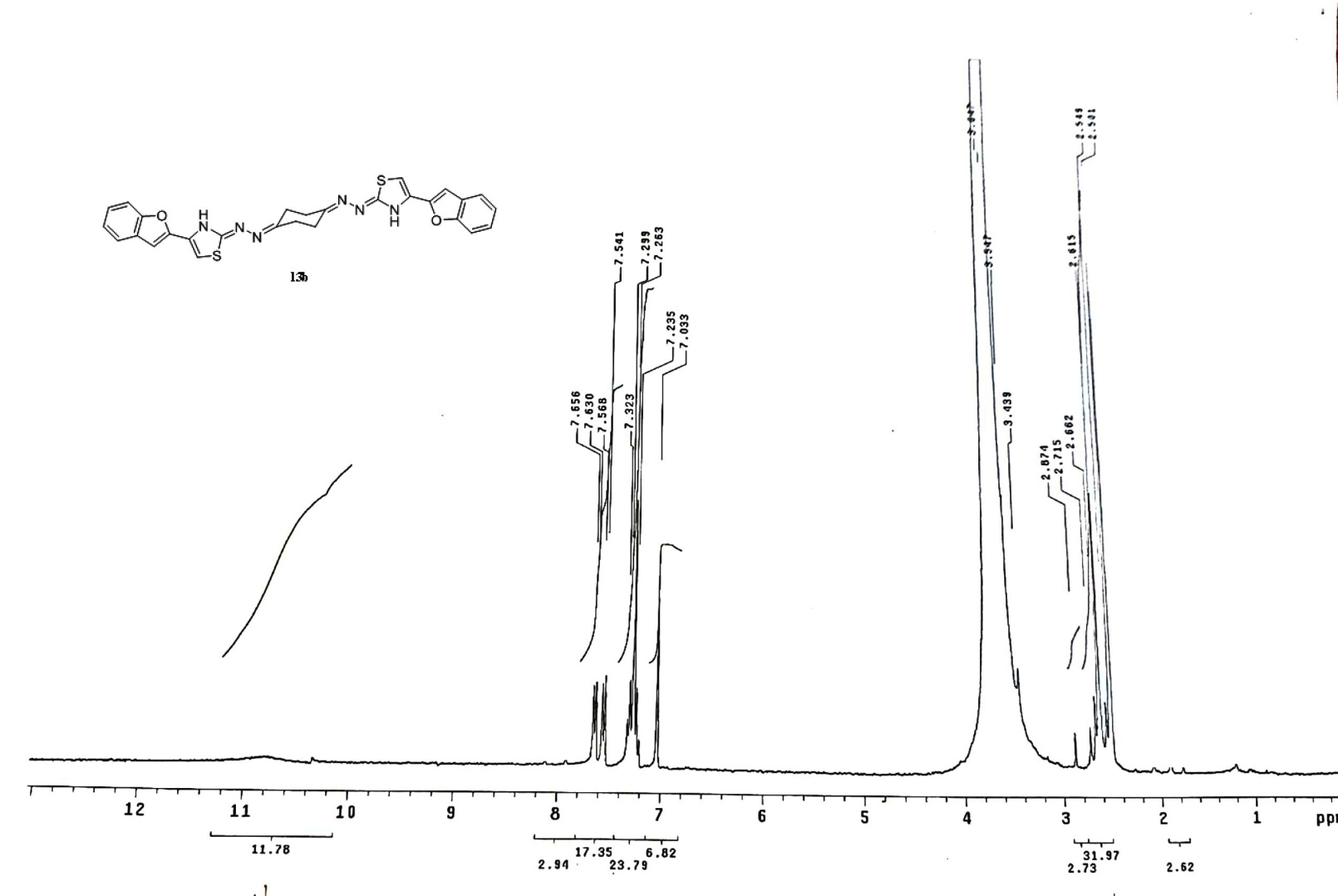


1H NMR spectrum of compound 13b


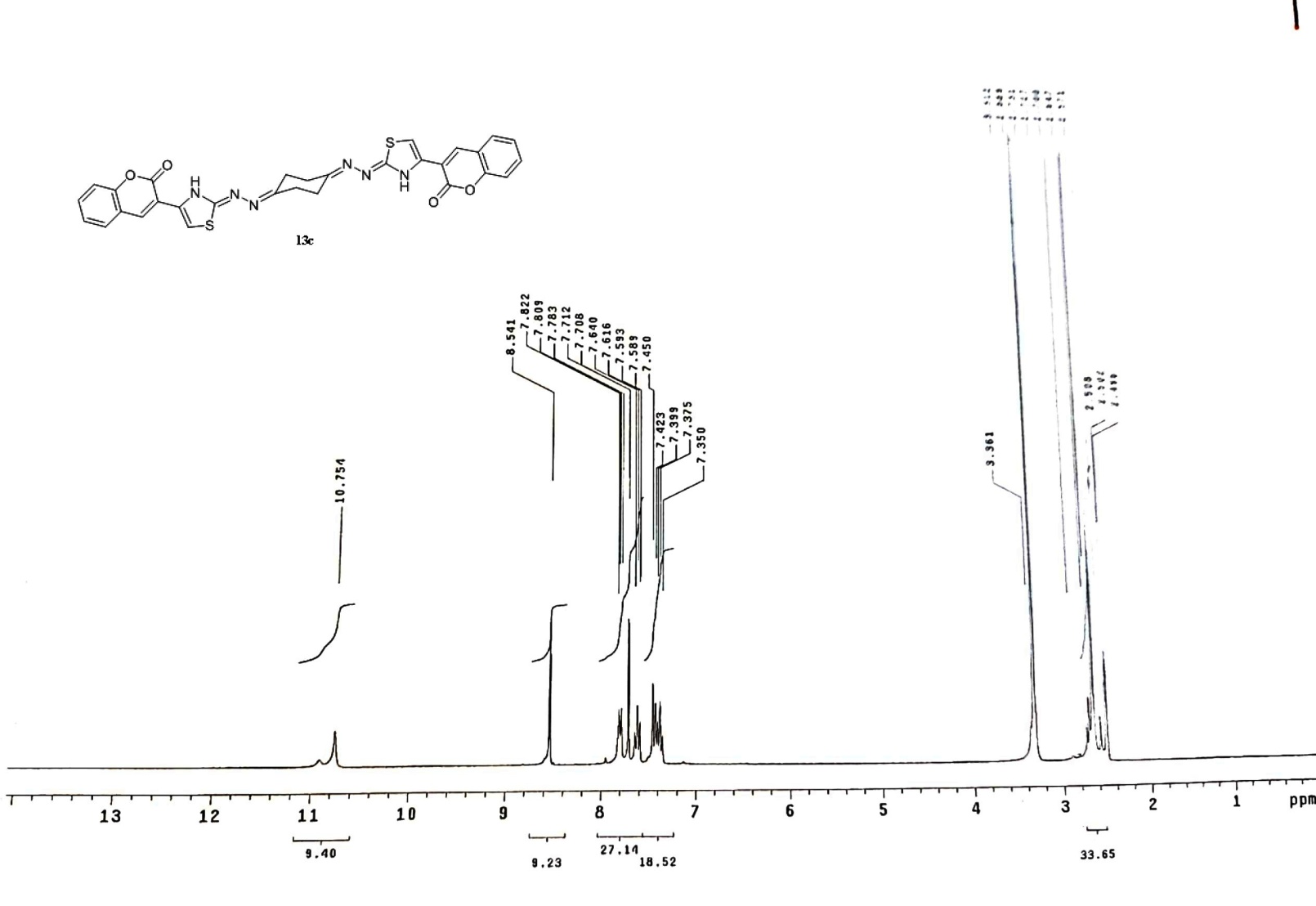


1H NMR spectrum of compound 13c


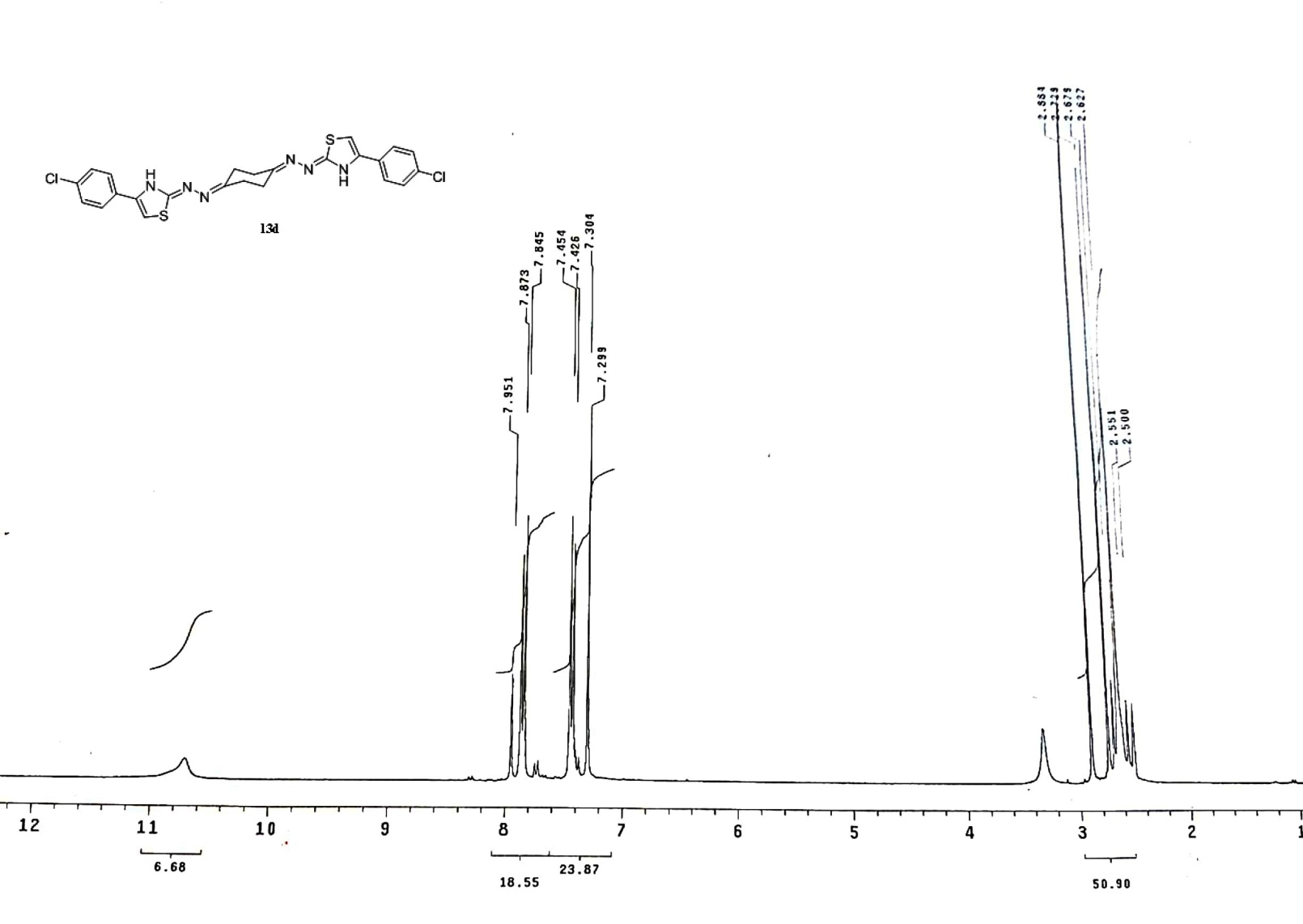


1H NMR spectrum of compound 13d


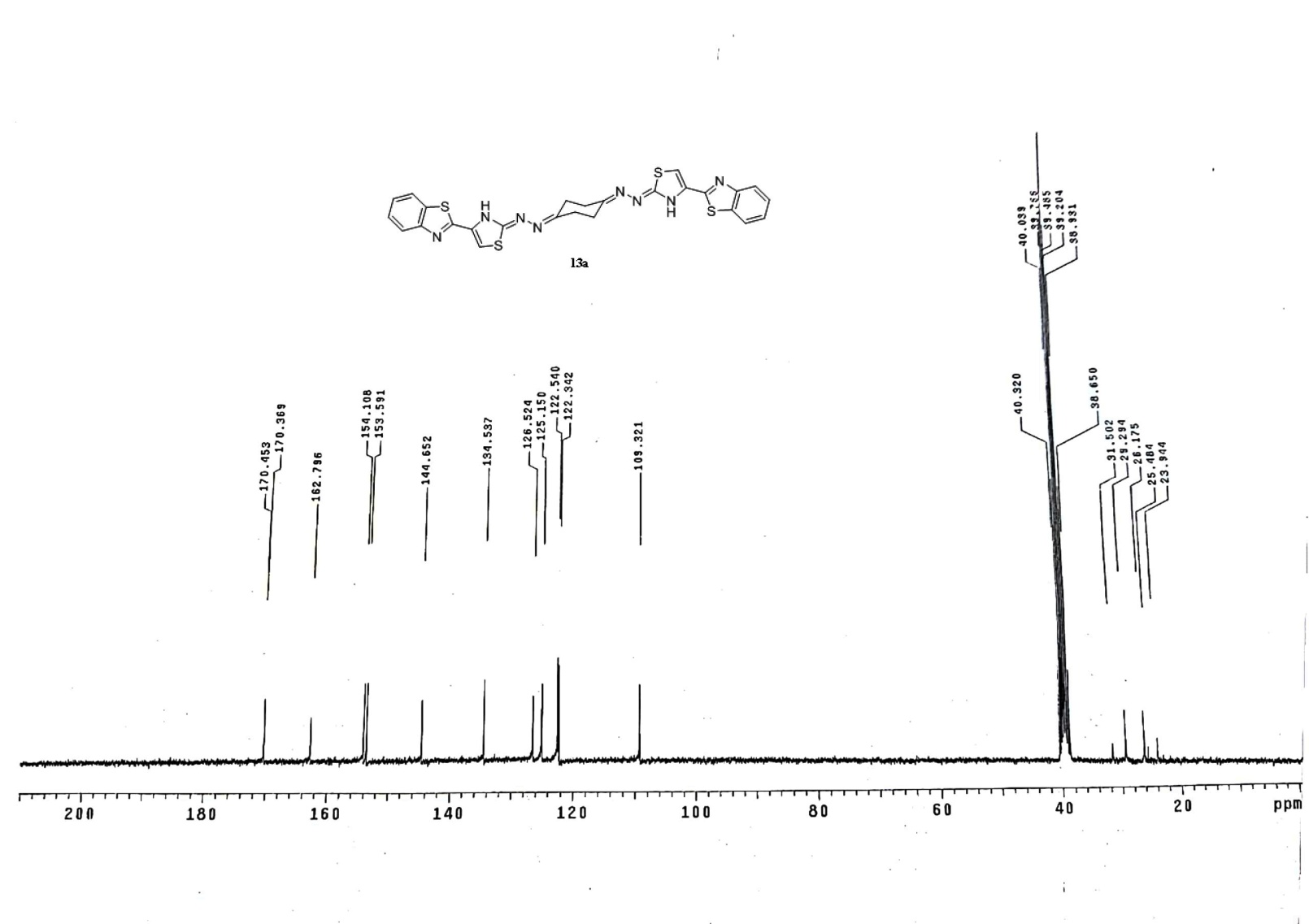


13C NMR spectrum of compound 13a


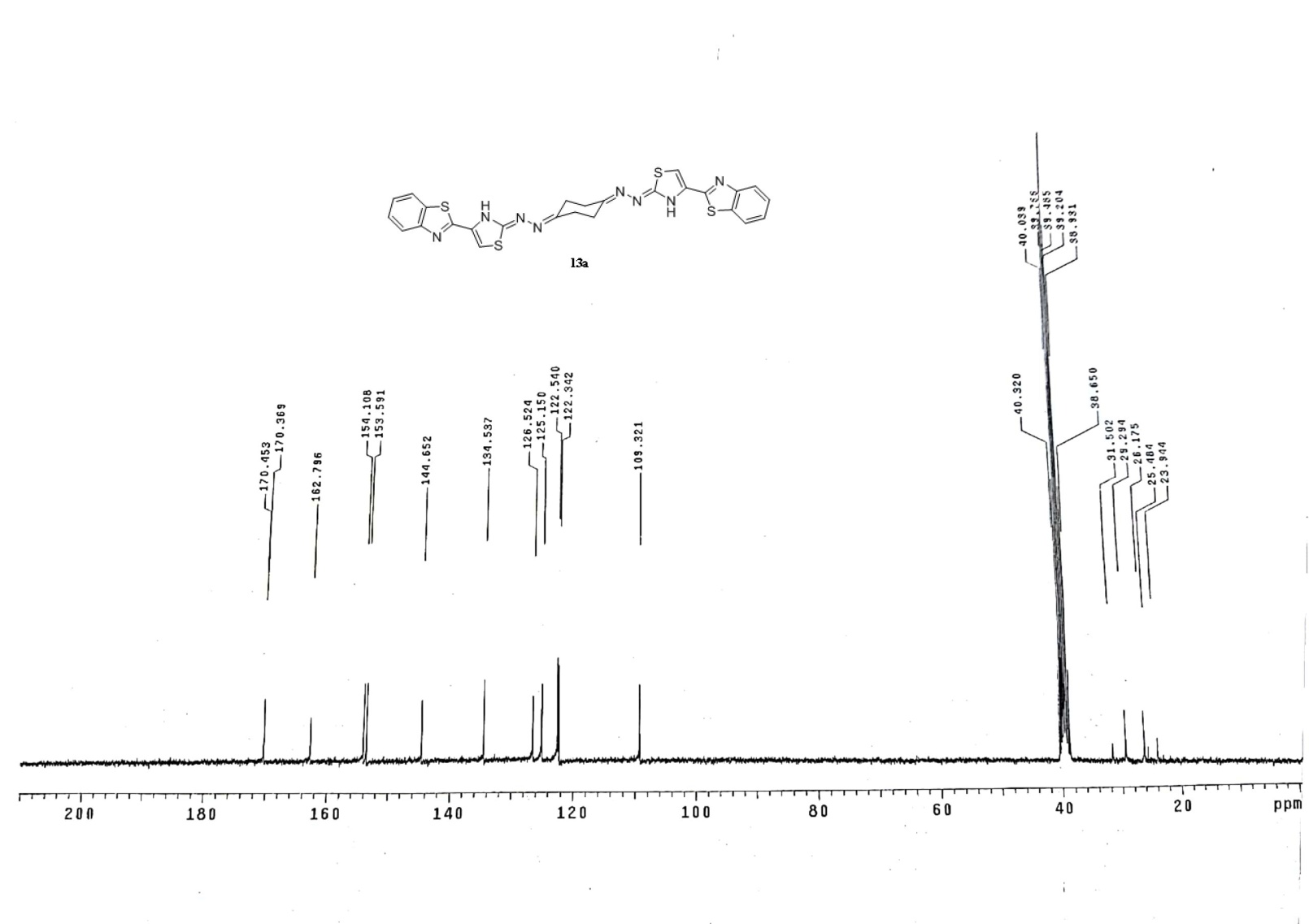


13C NMR spectrum of compound 13b


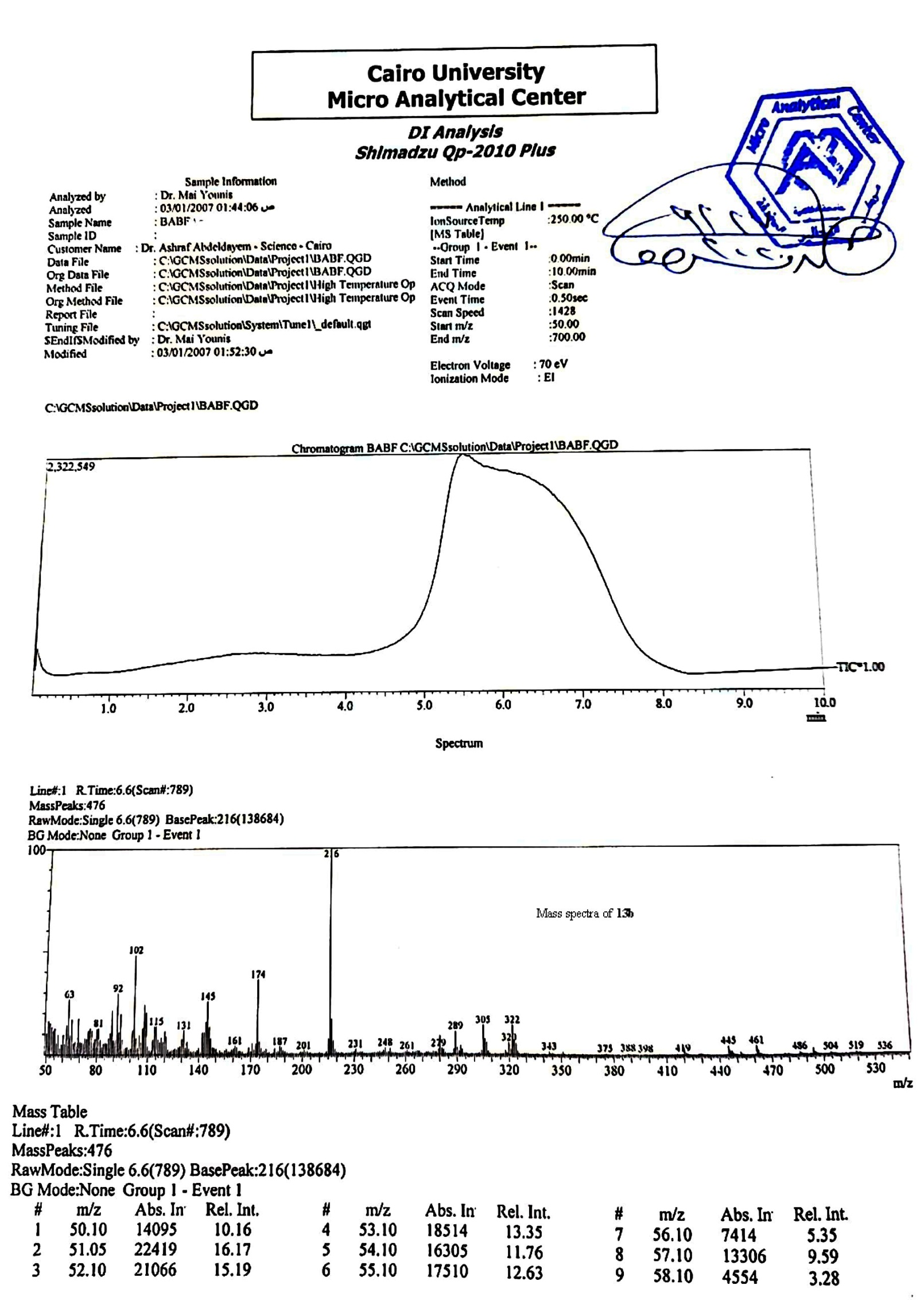


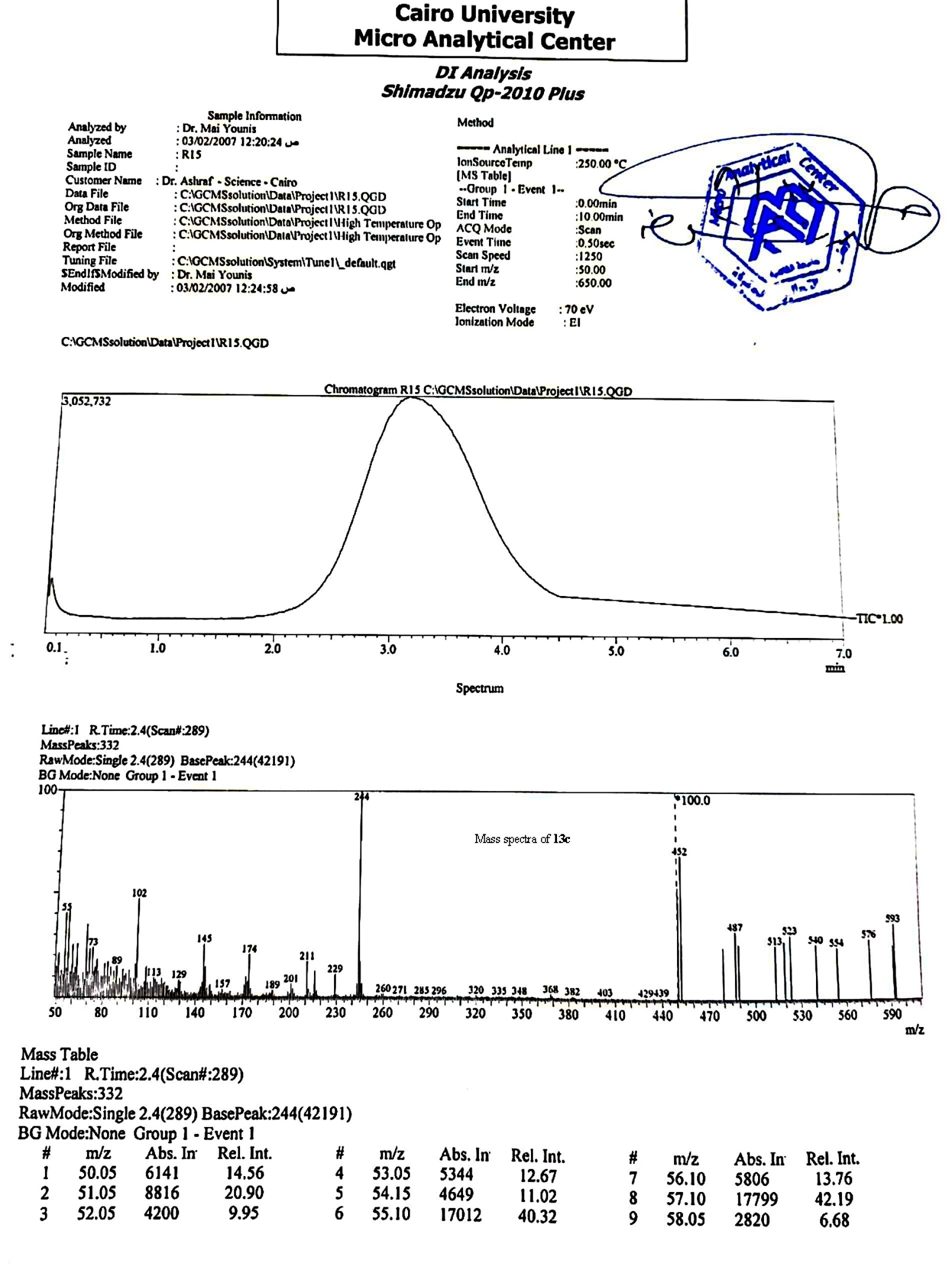

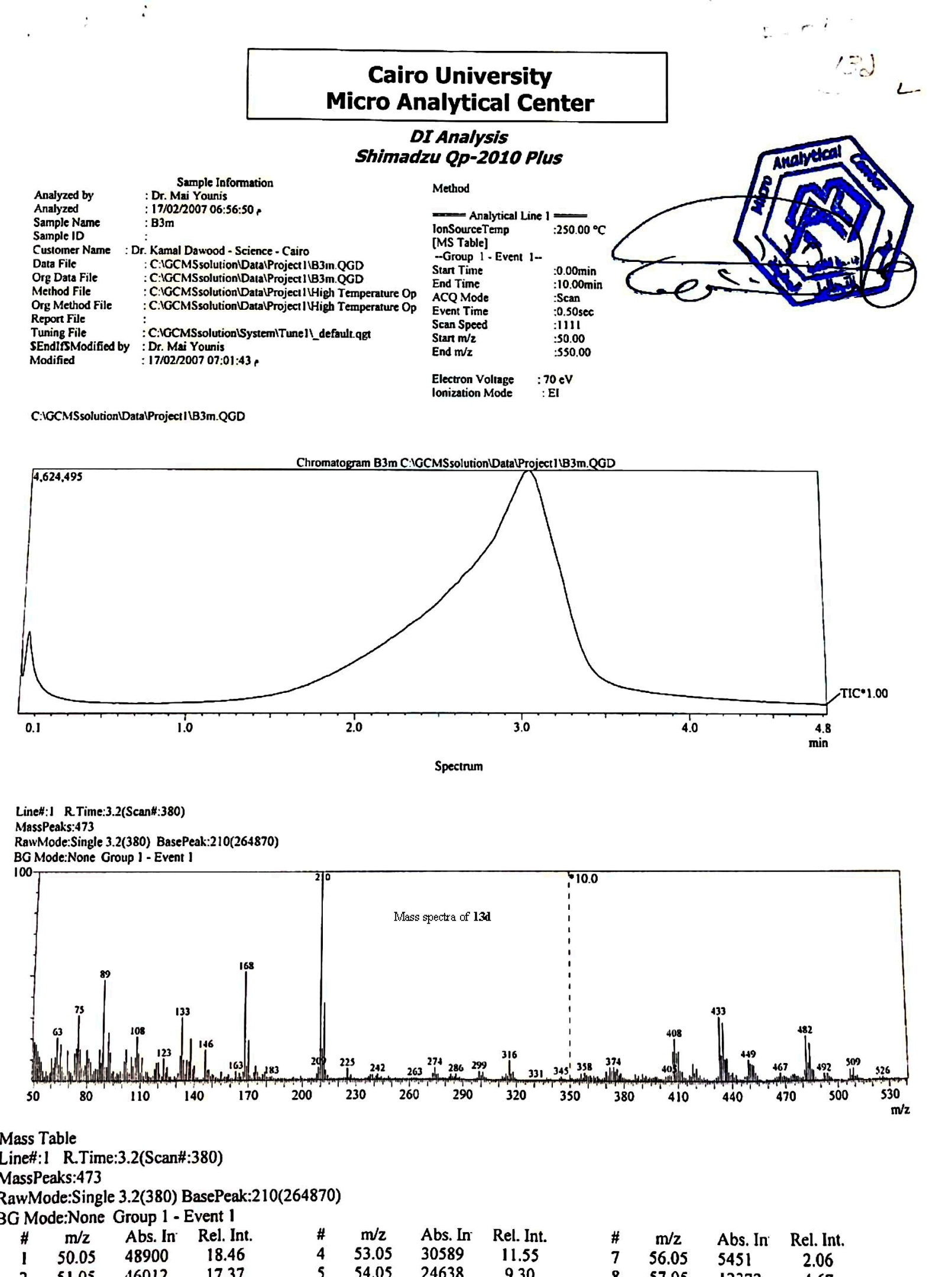

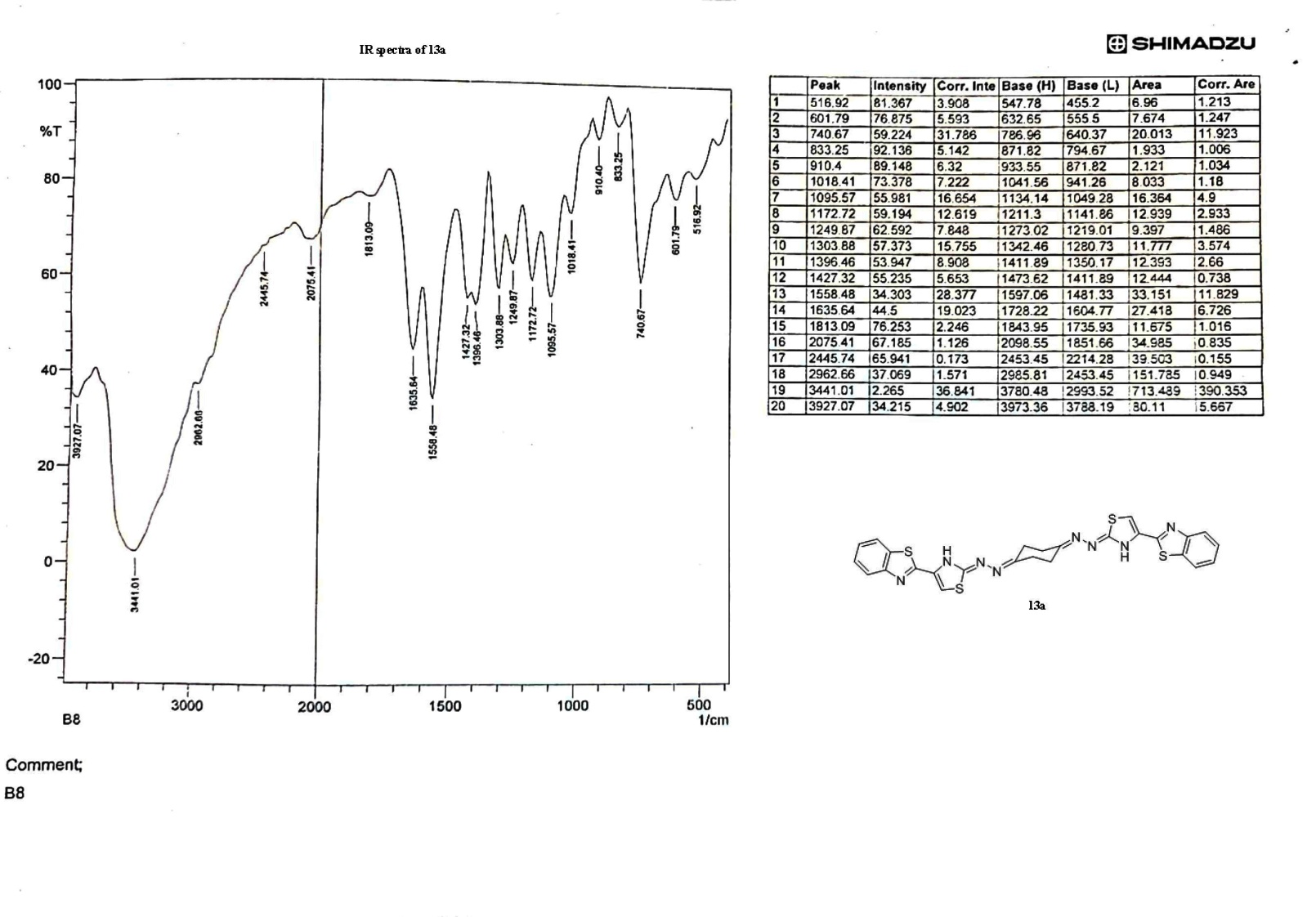

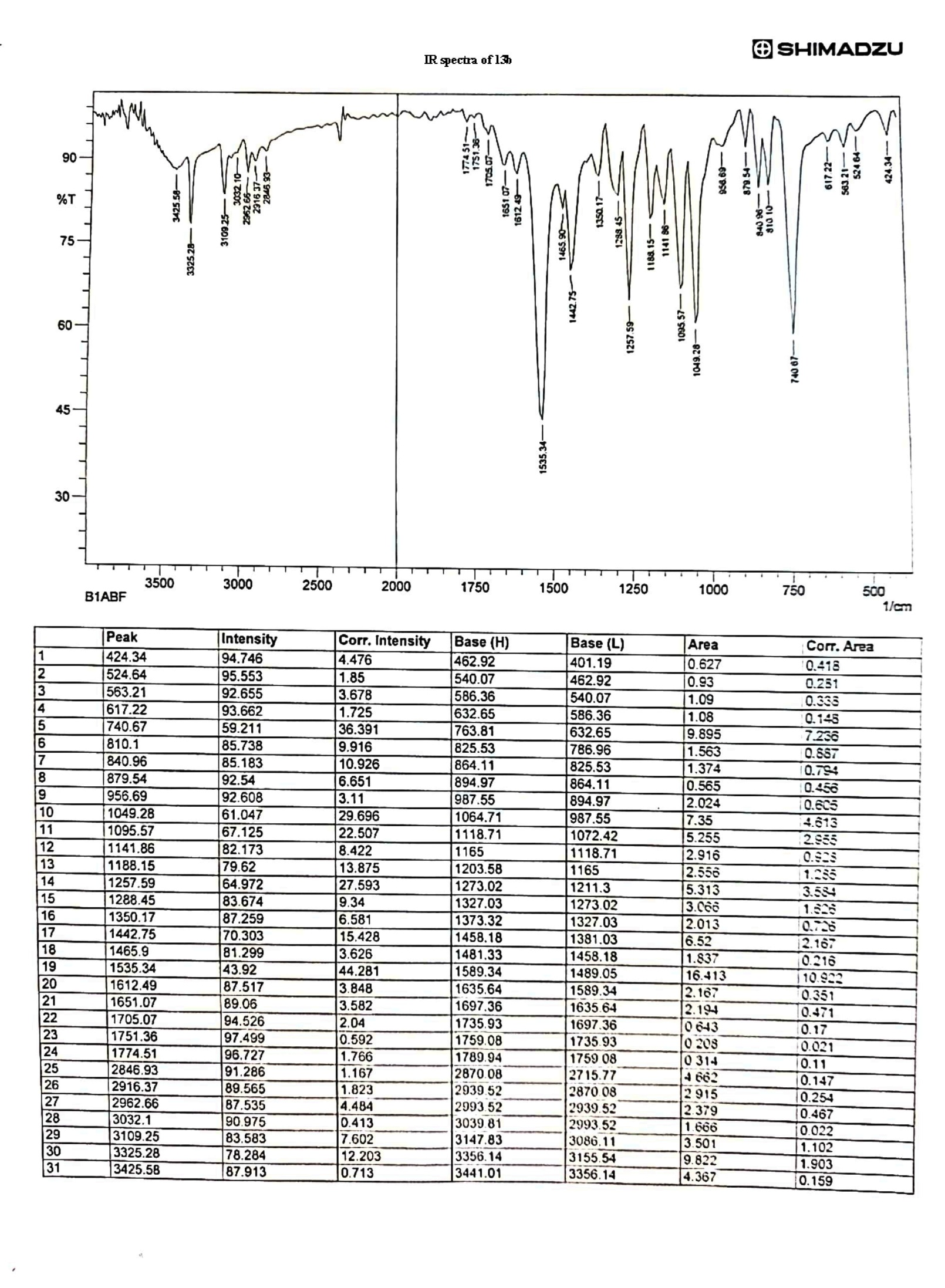

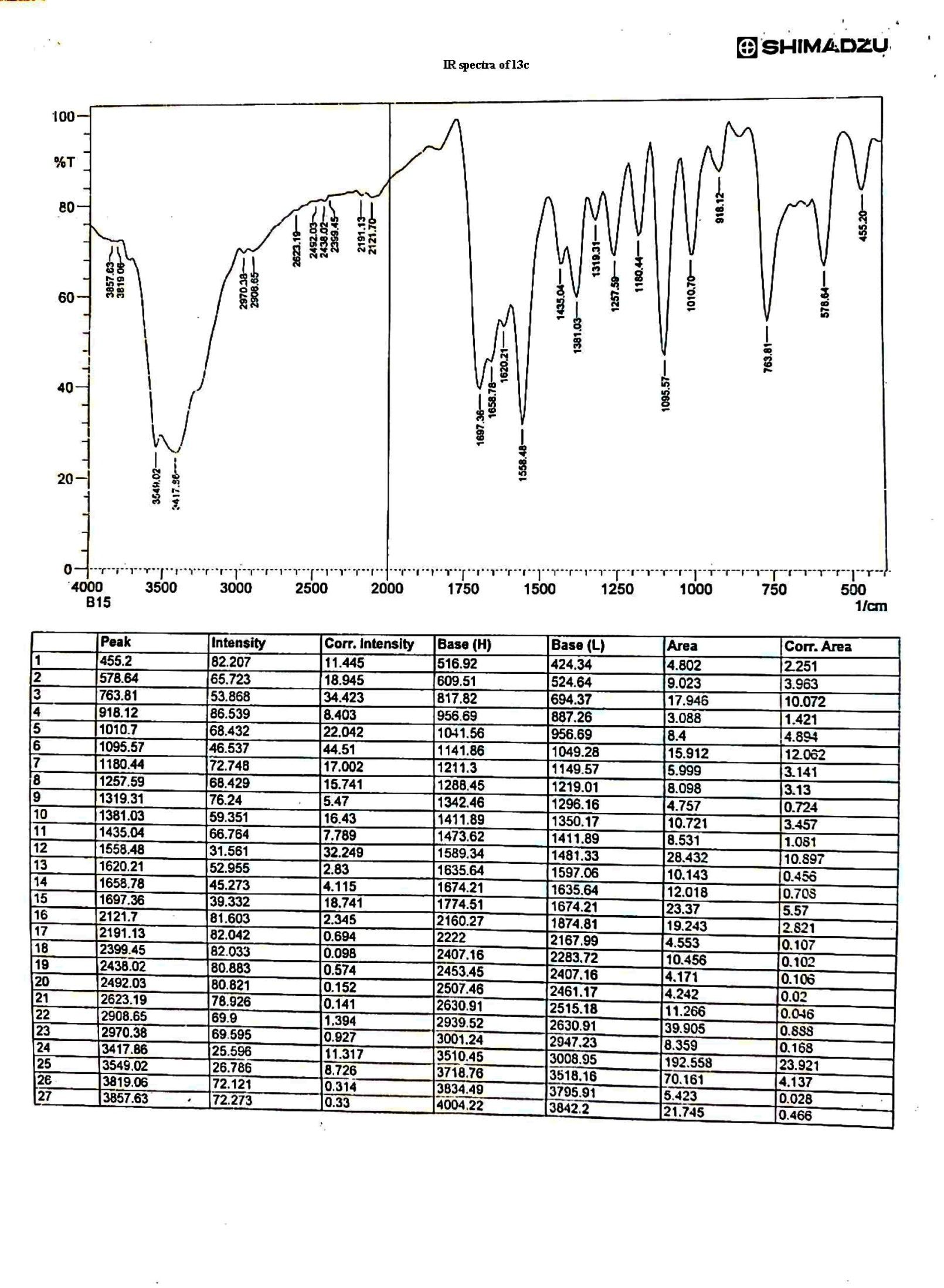


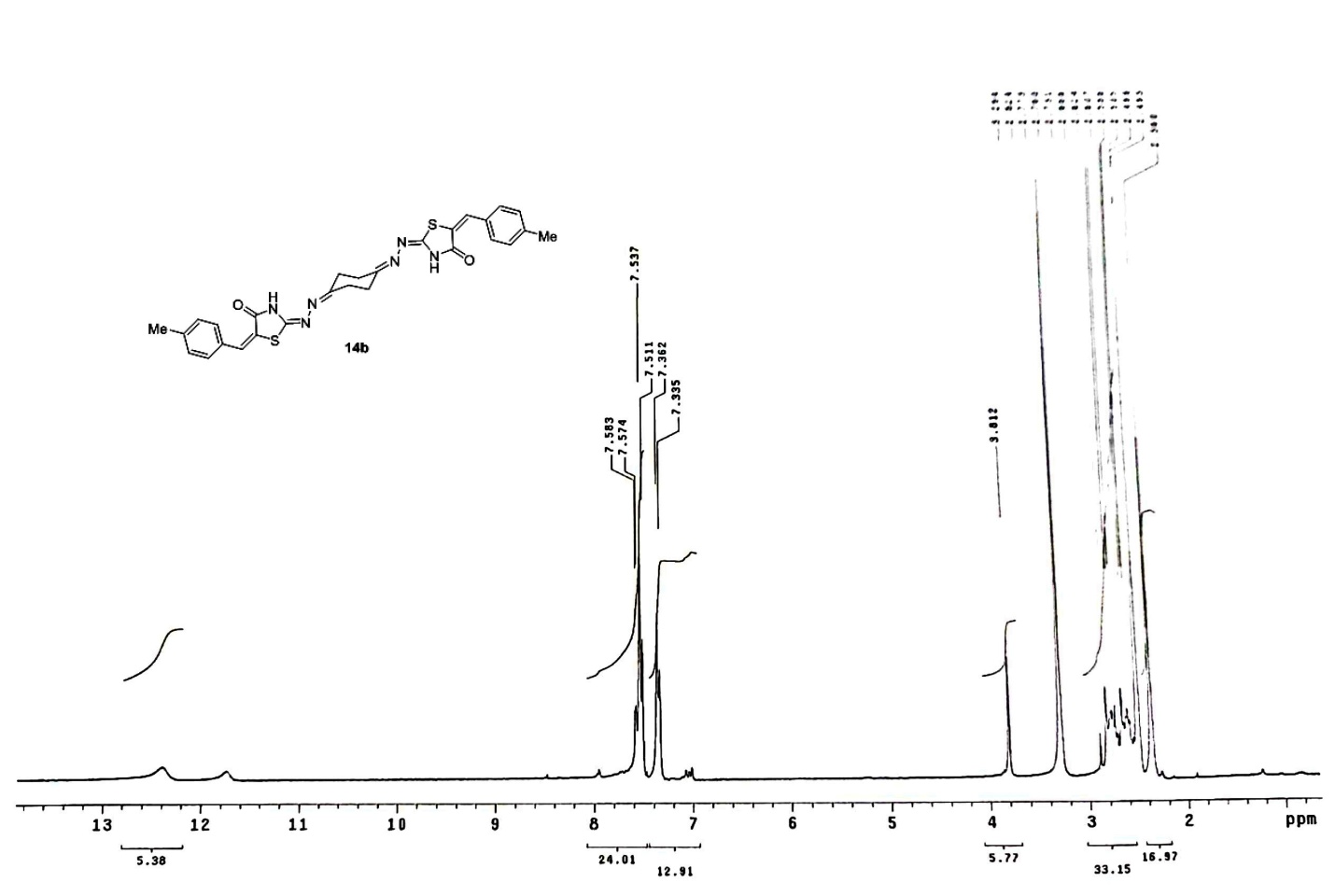


1H NMR spectrum of compound 14b


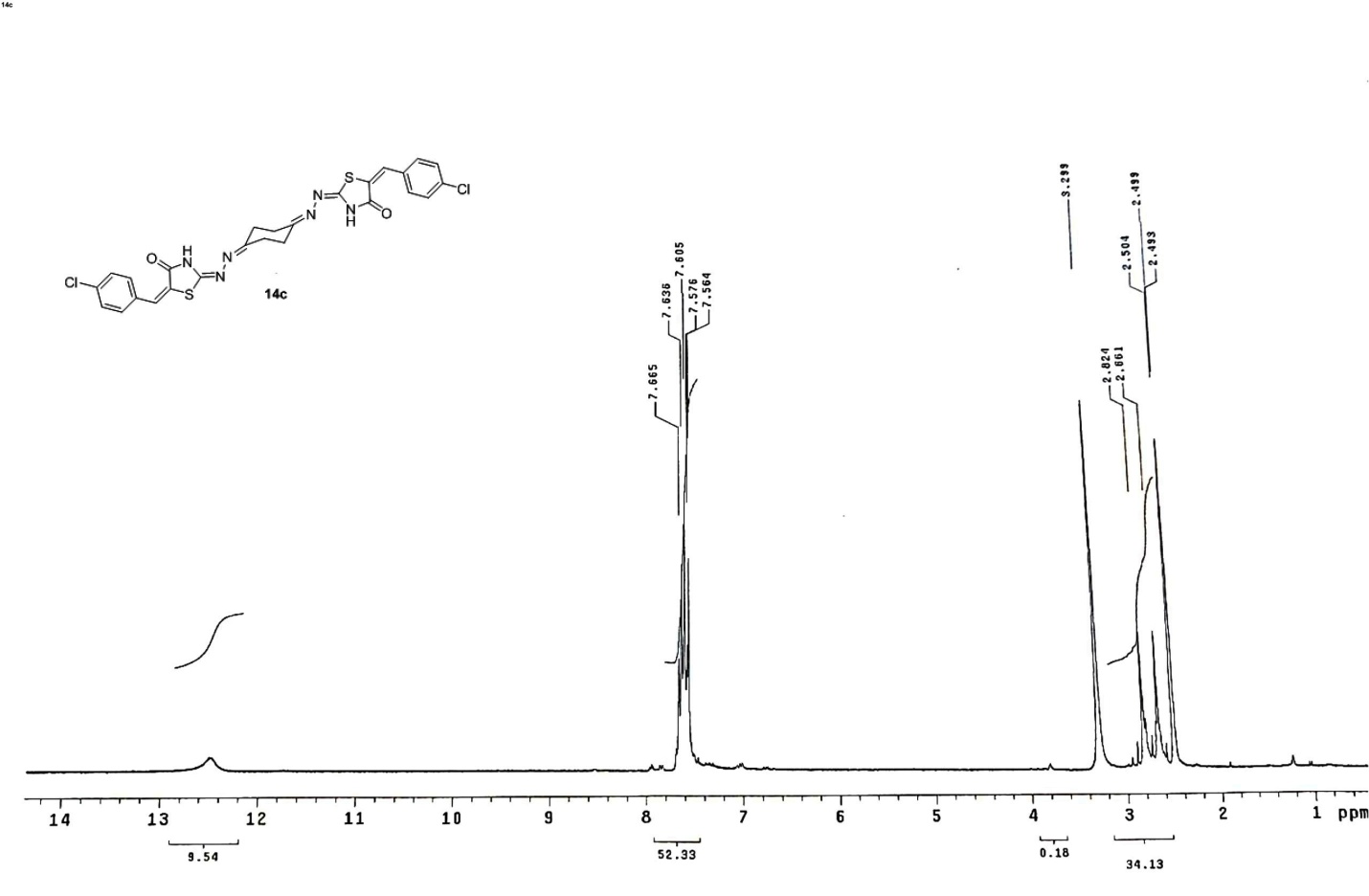


1H NMR spectrum of compound 14c


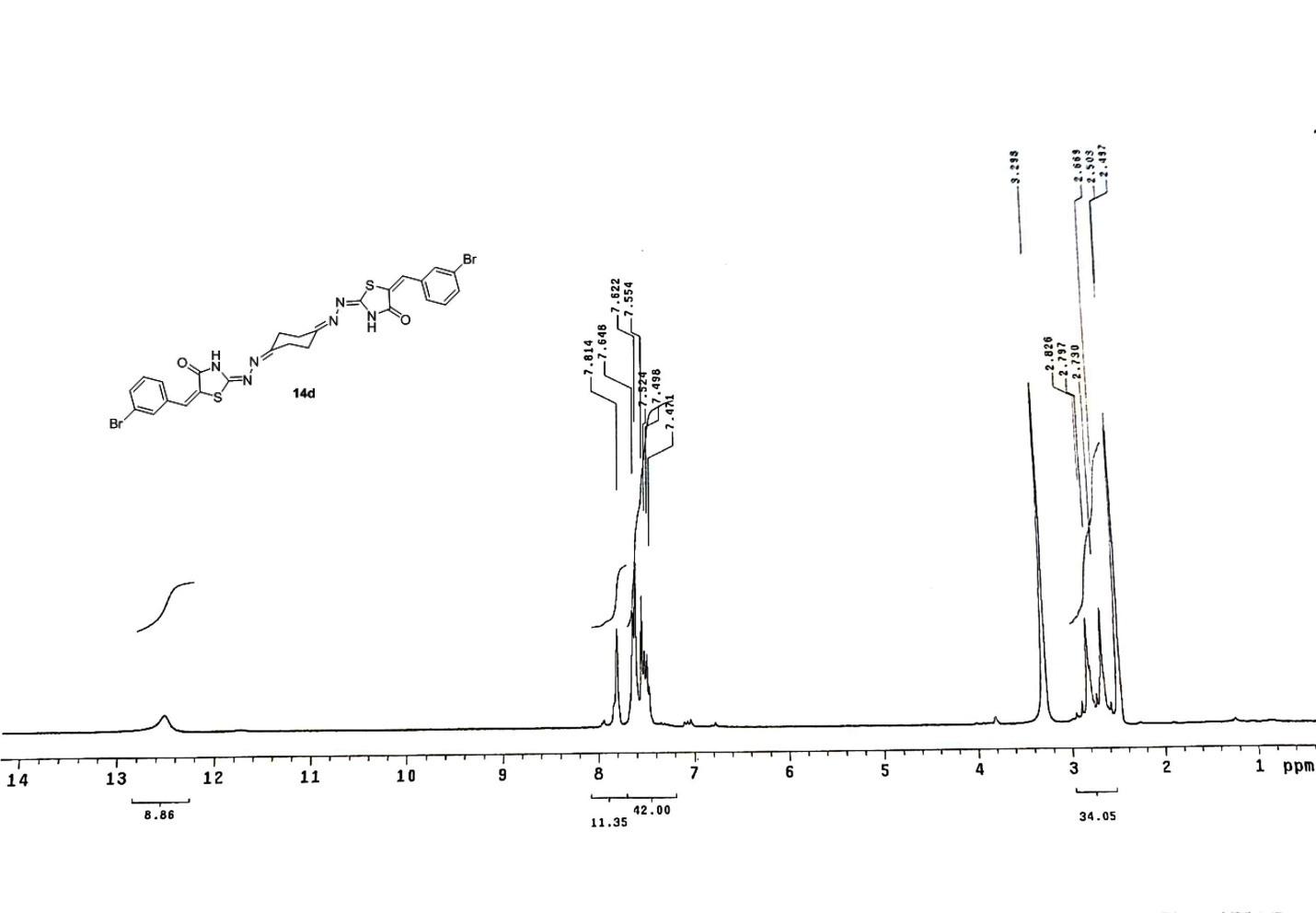


1H NMR spectrum of compound 14d


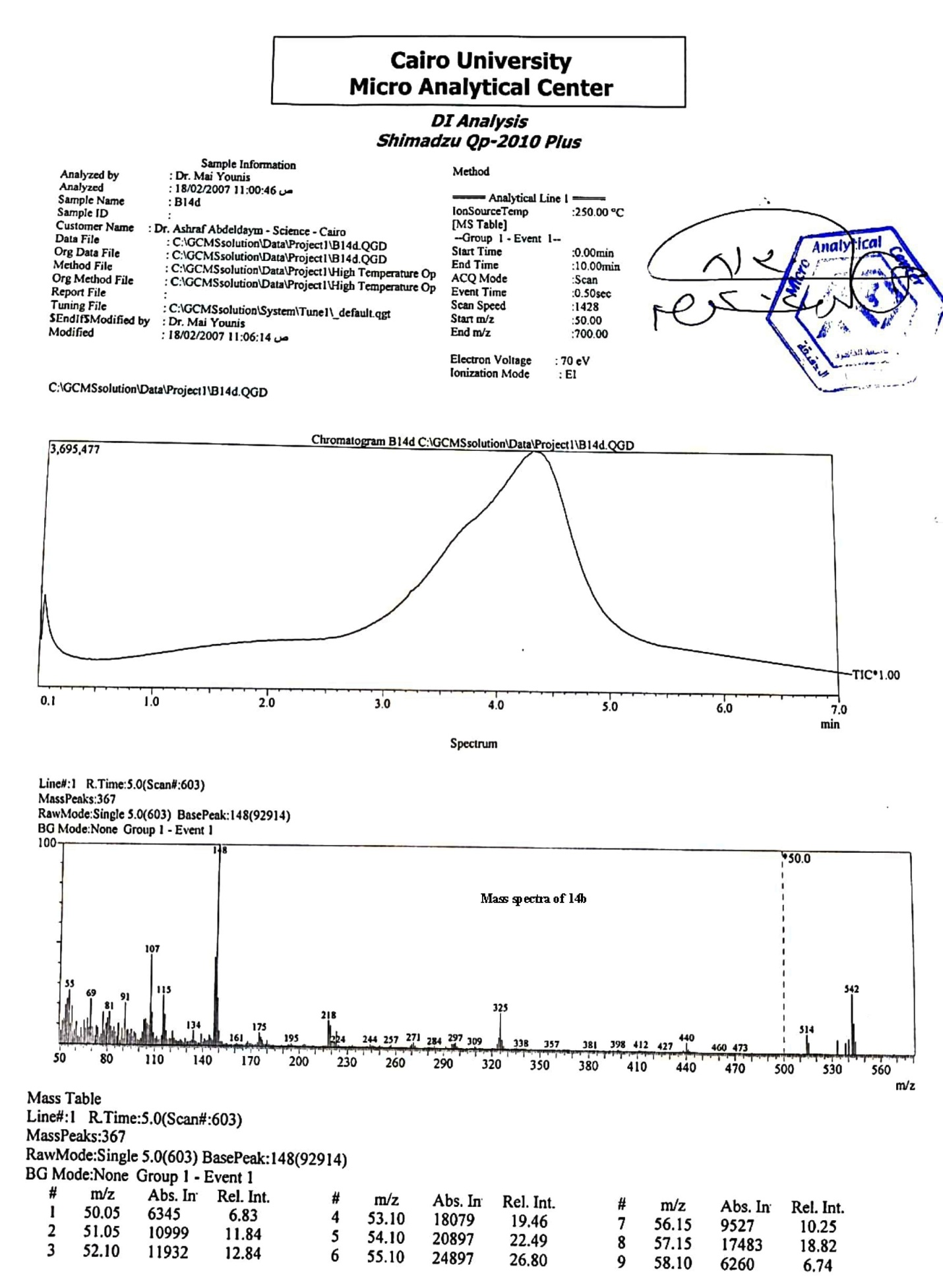


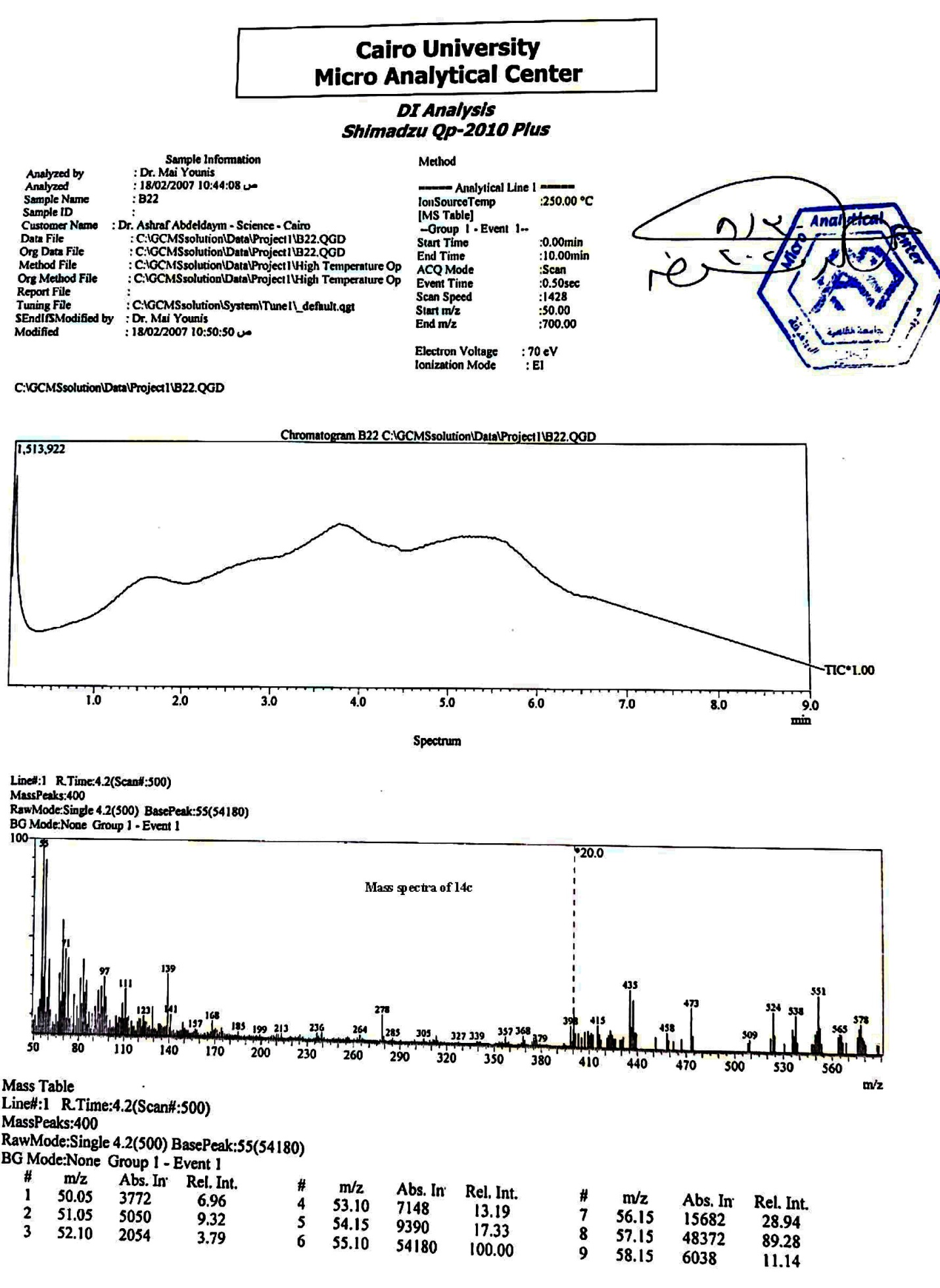


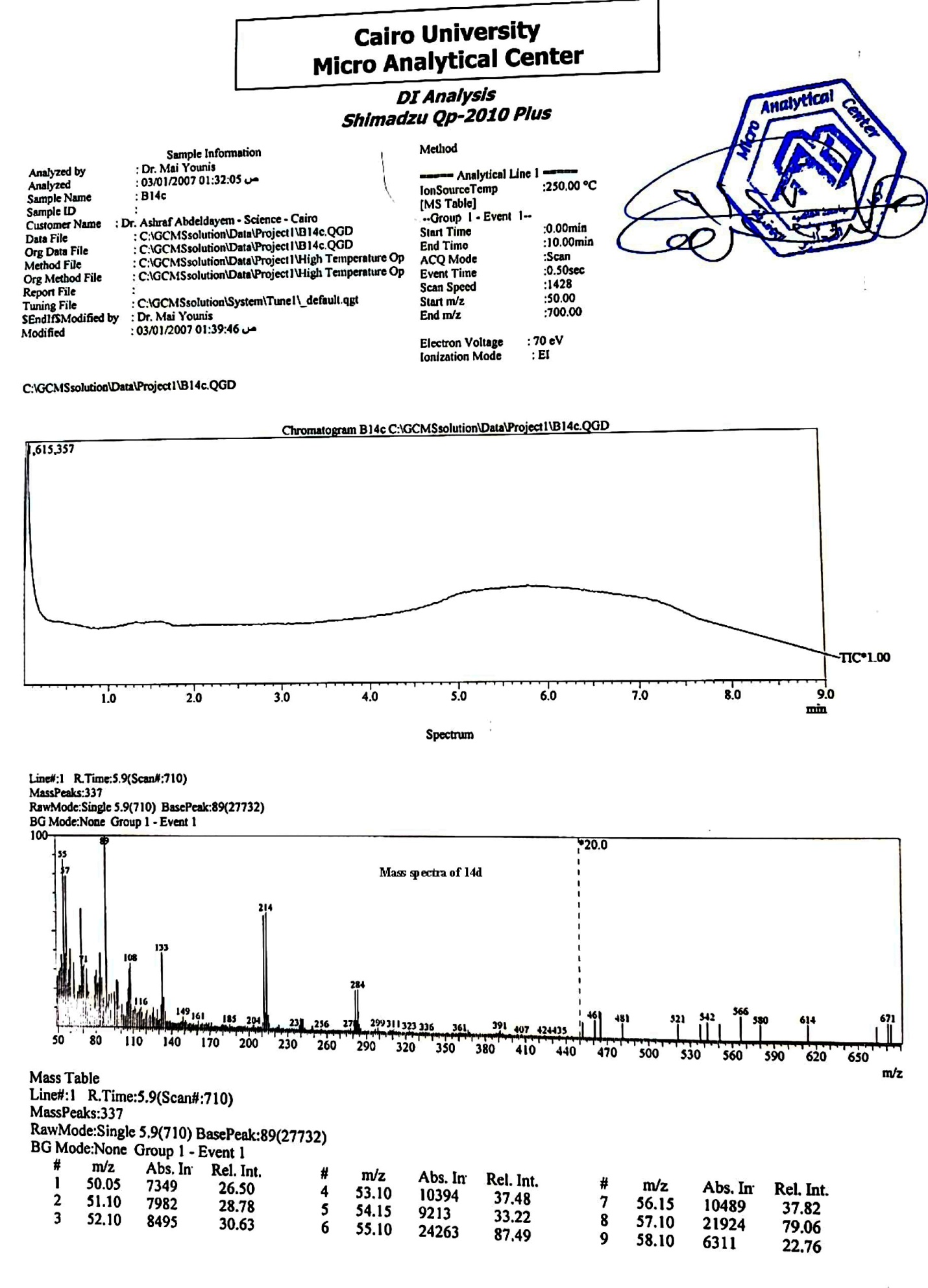


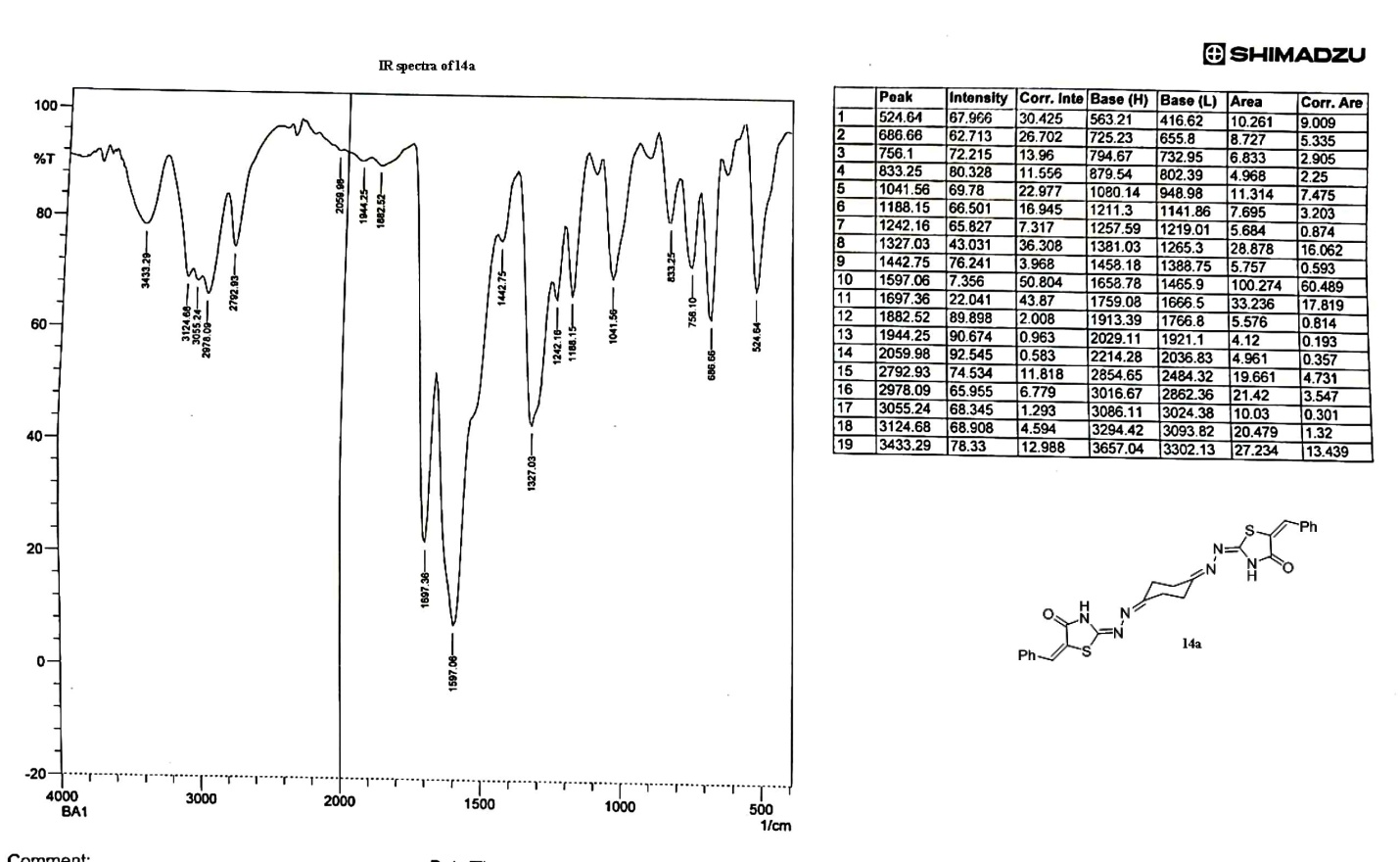


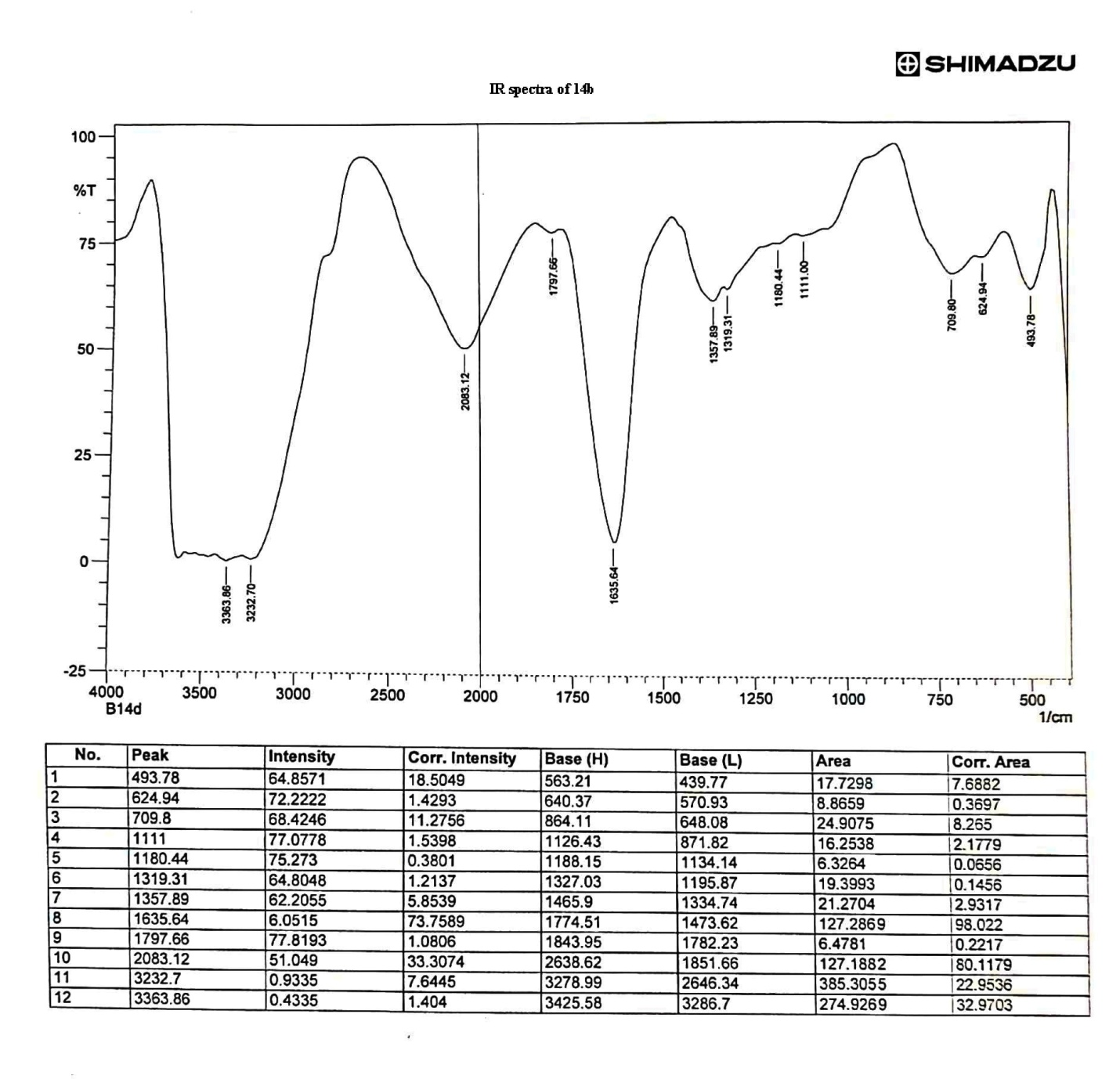


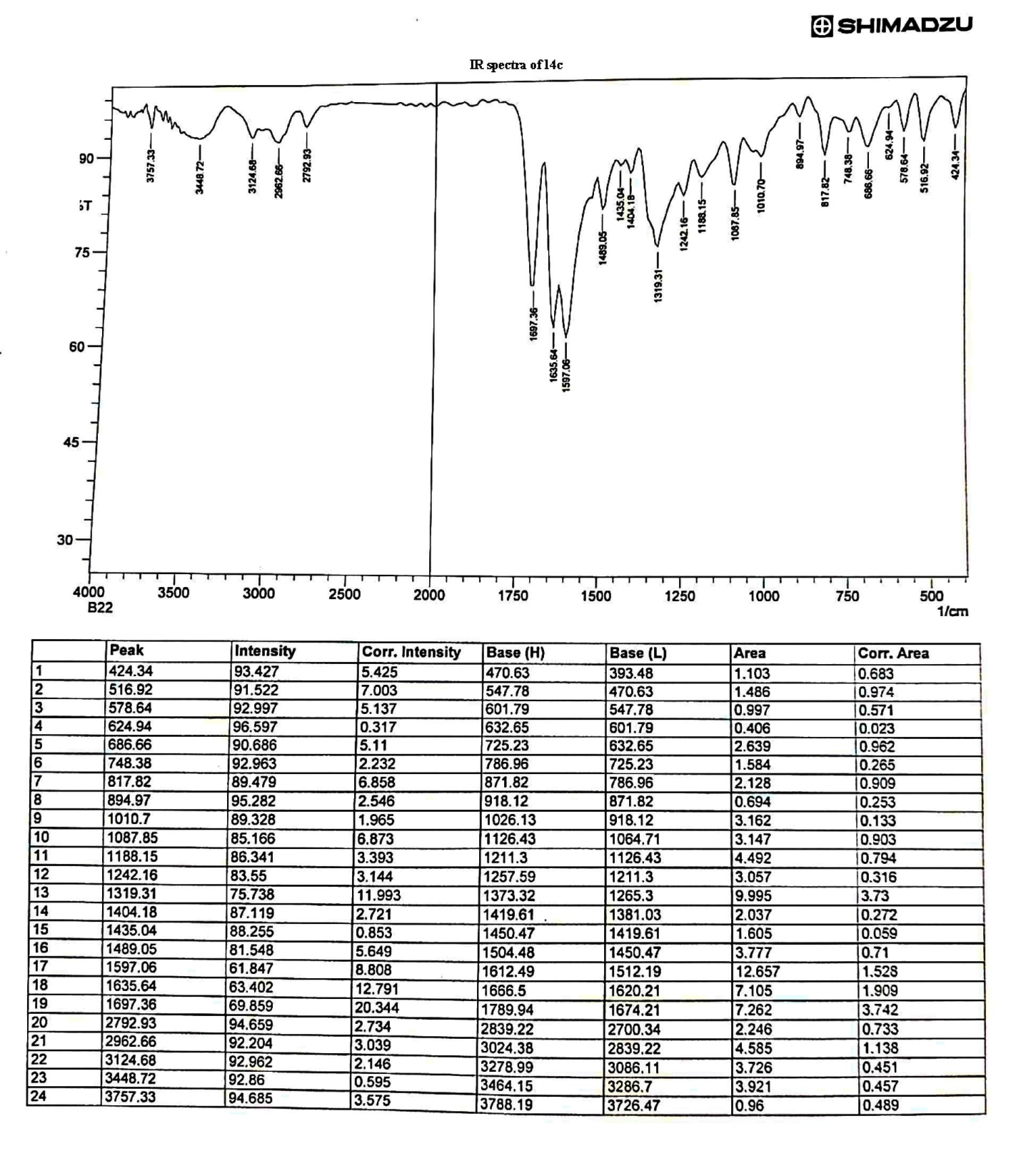

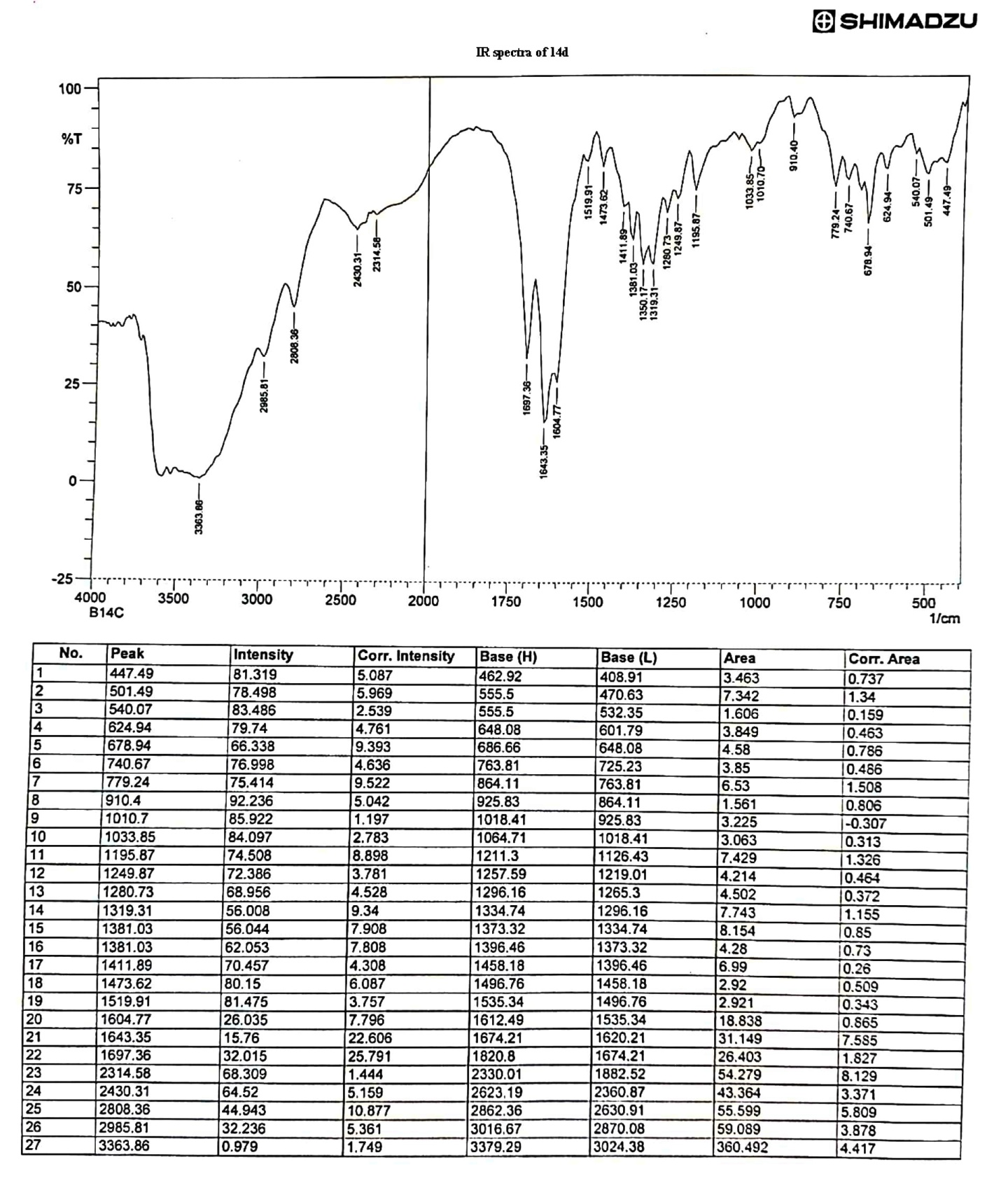

Supplement: Supplementary file 1 [file DataSheet1.docx]
